# Assessment of the Oral Health Perceptions and Behaviours of Adolescents in Bosnia and Herzegovina: A Cross Sectional Study

**DOI:** 10.3390/healthcare13111347

**Published:** 2025-06-05

**Authors:** Jasmin Habibovic, Kenan Demirovic, Edina Habibovic, Jasmina Mlaco Durek, Alisa Tiro

**Affiliations:** 1 Public Health Institution Živinice, 75270 Živinice, Bosnia and Herzegovina; habibi_ja@yahoo.com; 2 Private Practice for Dentofacial Orthopedics and Orthodontics “Demirović”, Bjelave 70, 71000 Sarajevo, Bosnia and Herzegovina; 3 Public Health Institution Gračanica, 75276 Gračanica, Bosnia and Herzegovina; edina.habibovic1@gmail.com; 4 Public Health Institution Bugojno, 70230 Bugojno, Bosnia and Herzegovina; jasmina.mlaco@yahoo.com; 5 Department of Orthodontics, School of Dental Medicine, University of Sarajevo, 71000 Sarajevo, Bosnia and Herzegovina; alisatiro@gmail.com

**Keywords:** WHO questionnaire, oral health, oral health behaviour, self-perceived oral health, dietary habits, adolescents

## Abstract

Background: The objective of this investigation was to assess the oral health (OH) of Bosnia and Herzegovinian adolescents in relation to differences and socioeconomic status (SES). Methods: This cross-sectional study included 306 school children from high schools located in the Tuzla Canton (Bosnia and Herzegovina). The sample consisted of 183 females and 123 males between 15 and 18 years old (mean of 16.82 years old). The study was conducted between December 2019 and March 2020, via an Annex 8 questionnaire from the World Health Organization (WHO), which collected information on OH behaviours, self-perceptions of oral health and dietary factors responsible for the OH of each subject. SES was categorized using five variables (occupation, education, income, place of residence and number of family members). Cross-tabulations were evaluated according to sex and socioeconomic status (SES) via the chi-square test. Results: Over 40% of the participants consumed sweets, cakes and biscuits on daily basis, whereas 41.5% of the participants visited a dentist only in the case of pain. The consumption of sweets (*p* = 0.024) and cakes and biscuits (*p* = 0.011) on a daily basis was significantly greater in female adolescents than in male adolescents. Compared with male adolescents, female adolescents reported occasional toothaches more frequently (*p* = 0.001) and were more dissatisfied with their dental appearance (*p* = 0.008) but presented a greater frequency of flossing (*p* = 0.001) and toothbrushing (3–5 times a day) (*p* = 0.0001). There was no association between the different levels (below average, average, above average) of SES and factors affecting OH status of adolescents. Conclusions: The study revealed significant sex differences in several factors affecting OH status and revealed no relationship between SES and OH behaviours or between perceptions and risk factors affecting OH in 15–18-year-old adolescents. Data obtained from this study might help in the creation of new OH prevention programs aimed at improving the OH status of adolescents in Bosnia and Herzegovina.

## 1. Introduction

Good oral health (OH) status represents a major part of overall health and quality of life [1]. The status of OH in one population is related to several factors, such as the frequency of regular visits to dental offices for check-ups, the frequency of toothbrushing, the use of proximal cleaning devices, sugar intake, the consumption of fluoride products and the consumption of fluoridated water [2,3,4,5]. OH status is also influenced by social and economic status, level of education, environmental factors and health-care services [6,7]. Another factor includes effect of soft diet consistency that leads to anterior teeth crowding and mouth breathing due to constricted airway, thus aggravating maintenance of oral hygiene [8]. On the other hand, the latest review found no connection between the third molars and anterior teeth crowding and diminished their adverse impact on OH status [9]. Furthermore, individuals with eating disorders more frequently develop oral diseases which generate a negative impact on their OH [10]. Opportune orthodontic treatment of adolescents with dental malocclusions could significantly improve overall OH [11]. Currently, the most common oral diseases, such as dental caries and periodontal disease, might be prevented by adequate preventive treatment [12,13].

To prevent the onset of early childhood caries, the American Academy of Pediatric Dentistry (AAPD) recommends toothbrushing twice daily, providing treatment with a fluoride varnish and avoiding the consumption of sugary foods and drinks beginning in early childhood [14]. A higher risk for caries is directly and positively correlated with irregular and improper oral hygiene maintenance. With the aim of improving overall OH status and oral health related quality of life (OHRQoL), the WHO continuous to work to further develop, improve and promote OH policy at the global level. Previous studies showed that Bosnia and Herzegovina is European country with very poor OH status and is within the five countries with the highest relative risk for caries [15,16]. Bad OH habits and behaviours adopted in childhood and adolescence may adversely affect OH status in adulthood [17]. Adolescence is a transitional period between childhood and adulthood during which children experience many biological, developmental and socioemotional transitions. Children’s perceptions and attitudes and OH behaviours evolve mostly during the middle childhood period. Factors such as geographical region, sex, socioeconomic status (SES) and organization of the health-care system has a significant effect on differences in findings related to OH perceptions and behaviours among adolescents [18,19]. A recent study of four Balkan countries, including Bosnia-Herzegovina, showed that participants did not perceive OH as a part of overall health [20]. Earlier investigations confirmed that differences in OH habits were related to different levels of SES [21,22]. Low SES was associated with a greater likelihood of oral health-risk behaviours in adolescents [23,24]. Evidence shows that oral diseases are mostly present in low-and middle-income countries with a low SES [25]. In Bosnia and Herzegovina prevention programs have been developed for elementary schools but no prevention programs have been developed for high schools. Bosnia and Herzegovina lacks national programs for the prevention of OH problems and protection of OH [15,26]. In general, the OH perceptions and behaviours of adolescents in Bosnia and Herzegovina are under-investigated. To our knowledge, no similar study including adolescents in the Tuzla Canton has been conducted until now. The significance of the study is strengthened by the opportunity for the first time to compare the OH status of adolescents from Tuzla Canton with OH of adolescents living in other parts of Bosnia and Herzegovina. Therefore, we aimed to evaluate the OH behaviours, perceptions and dietary habits of 15–18-year-old adolescents in Tuzla Canton (Bosnia and Herzegovina). Another aim was to investigate the associations between OH behaviours, perceptions and risk factors and socioeconomic status.

## 2. Materials and Methods

### 2.1. Subjects

This cross-sectional survey was conducted among adolescents attending public high schools in Tuzla Canton (Bosnia and Herzegovina). This canton has a total of 33 high schools in its thirteen municipalities. Four public high schools from several cities were randomly selected to represent the Tuzla Canton as the most populated canton among ten cantons in Bosnia and Herzegovina. Private schools were excluded from the study. Study included 306 randomly selected school-aged adolescents. The sample consisted of 183 female subjects and 123 male subjects. The period of adolescence encompassed by this study included adolescents aged between 15 and 18 years old (mean age of 16.82 years old). The inclusion criteria for the present study were as follows: students who were willing to co-operate, students without psychological disorders, students without progressive oral disease, students with no history of systemic disease and students with no previous orthodontic treatment. The exclusion criteria for the study were any type of orthodontic treatment, cognitive impairment, psychological disorders and drug addiction. From December 2019 and March 2020 OH perceptions, risk factors and behaviours of each subject were evaluated using a questionnaire of the World Health Organization (WHO) (Supplementary Material).

### 2.2. Ethical Consideration

Approval for the study was obtained from the Institutional Review Board of the School of Dental Medicine, University of Sarajevo. The study was performed in compliance with the Declaration of Helsinki Ethical Principles for Medical Research. Prior to participation, the parents were provided with detailed information about the study in Bosnian. Written consent was obtained from both the students and their parents.

### 2.3. Questionnaire

For the self-assessment of oral health, the World Health Organization (WHO) recommends the use of several questionnaires titled; “Oral health surveys: Basic methods —5th Edition”, which are provided on the WHO website [27]. In this survey, the WHO (Annex 8) questionnaire was used for the collection of data on self-assessed OH and risk factors for children or adolescents. The data obtained using this questionnaire provided information about the dental visits and oral hygiene habits, dietary habits, dental appearance related problems and self-perceived OH of each subject. The interview format of the questionnaire provided additional information about some of the questions, reducing the possibility of erroneous answers. The English version of the WHO questionnaire was translated into Bosnian by oral health specialists who were fluent in English, and the questionnaire was administered to all adolescents who agreed to participate in the study. The survey comprises 28 questions divided into three sections. The first set of questions are related to oral health behaviours, the second part includes questions related to self-perceived OH, and the third part includes questions related to the assessment of dietary risk factors. The WHO questionnaire (Annex 8) [27] was modified in the section related to SES and consisted of five variables (occupation of parents, education status, income per month, place of residence and number of family members). Examinations were performed by the same experienced investigator trained to record parameters related to OH status.

### 2.4. Statistical Analysis

In this study, all the statistical procedures were performed with the statistical package IBM Statistics SPSS v 23.0. Cross-tabulations were evaluated according to sex and SES via the chi-square test. In order to report significant sex differences, the odds ratios (OR) were calculated. The sample size was calculated based on the data of the number of adolescents in high schools in the Tuzla Canton, at ages between 15 and 18 years of 13051, according to the data of the Federal Statistical Office of Bosnia and Herzegovina. The minimum sample that will meet the criteria for confidence level of 90% and 5% margin of error was 267, and we managed to interview 306 high school students in four high schools. To evaluate sample reliability, Cronbach’s alpha reliability test was used.

## 3. Results

Table 1 presents a sociodemographic data of adolescents from randomly selected four high schools located in Tuzla Canton, Bosnia and Herzegovina. A total of 43.1% of the adolescents had a low SES, 45.8% had an average SES, and 11.1% had a high SES (Table 1).

### 3.1. Oral Health Behaviors

Analysis of the frequency of dental visits revealed that 16.0% of the participants had never visited a dentist in the last 12 months, 18.3% of the participants had visited a dentist once, 24.2% had visited a dentist twice, 15.7% had visited a dentist three times, 18.3% had visited a dentist four times and 7.5% did not know how many times they visited a dentist. There was a significantly greater number of males than females who never visited a dentist in the past 12 months (*p* = 0.005) and who visited a dentist 4 times in the past 12 months. There was no correlation between the SES of the participants and the frequency of dental visits in the past 12 months. A total of 41.5% of the participants visited a dentist due to pain or trouble with their teeth, gums or mouths, 32.4% of the participants visited a dentist for a routine check-up of their teeth, 10.5% visited a dentist for follow-up treatment and 15.7% did not know the main reason for visiting a dentist. With respect to brushing frequency, 92.8% of the participants brushed their teeth 2–6 times daily, 6.5% brushed their teeth once daily, and 0.3% never brushed their teeth or brushed their teeth 2–6 times weekly. Male adolescents brushed their teeth more frequently (twice daily) with a significant *p* value of 0.0001. No correlation was detected between SES and OH behaviours and oral hygiene habits. All of the participants (100%) used a toothbrush. The percentage of participants who used dental floss was 52.3% with females having using floss more frequently, with a significant *p* value of 0.001. Toothpicks were used by 37.6% of the participants with a significantly greater number of females using toothpicks (*p* = 0.023). Plastic toothpicks were used by 19.3% of the participants and Miswak was used by 5.2% of the participants, with no significant differences between female and male adolescents. Toothpaste was used by 99.3% of the adolescents, and toothpaste with fluoride was used by 12.4% of the participants with no statistically significant difference between the sexes (Table 2). No correlation was found between SES and factors related to OH behaviours (Table 3).

### 3.2. Perceptions of Oral Health

The participants described the health of their teeth as excellent (12.7%), very good (12.7%), good (48%), poor (4.2%), very poor (1.3%) and not known (4.2%). The participants described the health of their gums as excellent (26.1%), very good (28.1%), good (37.3%), poor (3.3%), very poor (1%) and not known (4.2%). A total of 12.1% of the subjects answered that they avoided smiling because of their teeth, while 84.0% of them did not avoid smiling, and 3.4% did not know if they avoided smiling. Only 2 (0.7%) participants reported that other children made fun of their teeth, 8 (2.6%) of them answered that they did not know if others made fun of their teeth, whereas 296 (96.7%) denied that others made fun of their teeth. Almost all of the subjects (96.1%) answered that they were not forced to miss classes at school due to toothache or discomfort caused by their teeth. A total of 91.5% of the participants reported that they did not have difficulty biting hard foods, and 94.1% reported no difficulty chewing. For the above mentioned factors, no statistically significant differences were found between the sexes. When considering the participants’ satisfaction with the appearance of their teeth, 37% of the participants reported that they were satisfied with the appearance of their teeth, 52.3% were not satisfied, and 10.3% were not sure and answered that they did not know whether they were satisfied. Female adolescents were less satisfied with the appearance of their teeth with a statistically significant difference (*p* = 0.008). A total of 22.9% of the participants had occasional pain or discomfort, 47.4%—rarely experienced pain or discomfort, 26.5%—never experienced pain or discomfort, and 3.3% did not know if they experienced pain or discomfort. Female adolescents more frequently experienced occasional pain and discomfort in comparison to male adolescents (Table 4). There was no significant correlation between SES and factors related to self-perceived OH (Table 5).

### 3.3. Dietary Habits

An analysis of dietary habits revealed that 10.8% of the participants consumed sweets several times a day, 43.1% consumed sweets every day, 30.7% consumed sweets several times a week, 6.2% consumed sweets once a week, 3.9% consumed sweets several times a month and 5.2% never consumed sweets. Evidence of the frequency of consumption of cakes and biscuits showed that 13.1% of the subjects consumed cakes and biscuits several times a day, 40.5% consumed them every day, 28.4% consumed them several times a week, 10.8% consumed them once a week, 3.6% consumed them several times a month and 3.6% never consumed them. A preponderance of females was observed for the daily consumption of sweets (*p* = 0.024) and cakes and biscuits (*p* = 0.011). Male adolescents more frequently consumed coffee with sugar when compared with female adolescents (*p* = 0.023). No sex differences were found with respect to the consumption of other dietary factors (fresh fruit, soft drinks, jam/honey, chewing gum, milk with sugar, and coffee with sugar) in this study. A correlation between SES and dietary habits was found only for the habit of drinking coffee with sugar, whereas participants with a high SES consumed significantly less coffee with sugar than did participants with an average or low SES (*p* = 0.030). The most relevant differences and similarities related to the results of the frequency of consumption of other dietary factors (fresh fruit, soft drinks, jam/honey, chewing gum, milk with sugar, coffee with sugar) affecting OH, are presented in the Table 6. No significant correlation was found between SES and dietary habits (Table 7). In the tables, the main differences and similarities are presented. The survey consisted of 21 items, and the value for Cronbach’s alpha was 0.737, indicating that the reliability of the sample was acceptable.

## 4. Discussion

Bosnia and Herzegovina lack surveys of oral health conducted at the national level, as no such a survey has been conducted in the past 30 years. This is the first study that aimed to evaluate the risk factors, behaviours and self-perceptions of OH status among adolescents in the Tuzla Canton (Bosnia and Herzegovina). Previously, the poor oral OH of adolescents of Bosnia and Herzegovina was indicated by very high DMFT and SCI scores [16,28]. This study revealed a significant disproportion between self-perceived dental and periodontal health and the frequency of dental visits due to toothache or periodontal problems. Most of the participants perceived their dental and periodontal health as good or very good (90%), although 41.5% of them had to visit dentist due to dental or periodontal problems. This study revealed that although 76.47% of the adolescents visit a dentist at least once a year, for 41.5% of them, the main reason for the visit was toothache. Compared with other national studies, our findings corroborated the findings of Davidovic et al. [29] (43.9%) and were higher than findings of Obradovic and Dolic [30] (25.5%). Additionally, our results are in accordance with the results of other nonnational studies [31,32,33]. During the last 12 months females visited a dental office more frequently than males (*p* < 0.005). The percentage of adolescents (93.16%) who brushed their teeth 2–6 times daily was almost identical to that of their peers from the capital of Sarajevo (92.85%) and substantially greater than that of their peers from the country’s second largest city of Banja Luka (56%) and from Eastern Bosnia and Herzegovina (54.9%) [29,34,35] In this study, the prevalence of adolescents who brushed their teeth 2–6 times daily was greater compared to that of their peers from Portugal (87.9%), Romania (67.3%), Sweden (89.2%), Croatia (70%), Italy (83%) Southeast China (50.9%) [19,31,36,37]. Females showed less frequent brushing behaviour twice a day in comparison to males, which does not align with previous surveys (*p* < 0.0001) [38,39]. Almost all adolescents used toothbrushes and toothpaste to clean their teeth, which was in accordance with previous findings [30,37,39,40]. Even though all the participants used toothbrushes and toothpaste to clean their teeth, only 12.4% of them used toothpaste with fluoride, similar to the results of other national studies [29,30,34] and significantly lower than those of recent international studies [19,41]. Among the 52.3% of adolescents who flossed their teeth, boys were more likely to avoid flossing teeth than girls were (*p* < 0.0001). Flossing frequency was significantly greater in this study than in other studies [19,30,37]. When dental appearance was considered, more than 50% of the subjects were dissatisfied and reported negative perceptions of their dental appearance. This value was significantly higher in comparison to reports of adolescents from China (24%) and Australia (31%) [42,43]. The percentage of adolescents (12%) who avoid smiling due to the appearance of their teeth in this study was not particularly different from the findings of other studies [43,44]. In our study, male adolescents were more satisfied than female adolescents with the appearance of their teeth (*p* = 0.008). There is evidence that juveniles are more sensitive to a variety of impacts, such as appearance, relative to older individuals. The importance of oral health-related quality of life (OHRQoL) is particularly relevant for adolescents from the aspect of the ability to contrive a social connection with other people [45]. Satisfaction with dental appearance is closely related to dental malocclusion wherein OHRQL significantly improves when dental malocclusion is corrected with orthodontic treatment [46,47]. In this study, 22.8% of the adolescents reported occasional pain related to their teeth and jaws, where 5–10% of the participants reported chewing difficulties and difficulty biting hard foods, suggesting that orthodontic or temporomandibular joint treatment might prevent further deterioration of signs and symptoms related to those complaints [48]. It was determined that adolescents in the Tuzla Canton consume more sweets on a daily basis (43.14%) compared to adolescents from the two largest cities in the country, Sarajevo (38.5) and Banja Luka (39.14%) and less frequently than their peers from the Central Bosnian Canton (56.8%) [30,49,50]. The frequency of sweet consumption once and several times a day (53.9%) was almost the same as that in Sarajevo (53.2%). and agreed with the results of previous studies [49,51]. In the present study, female adolescents had worse results in terms of certain oral health behaviours and perceptions than male adolescents did. The frequency of eating cakes and biscuits (*p* = 0.011) and sweets (*p* = 0.024) was more significant in females than in males, which could be a reason why female adolescents more frequently reported an occasional pain related to teeth or gums (*p* < 0.0001). Additionally, female adolescents less frequently brushed their teeth twice a day (*p* < 0.0001) and were more dissatisfied with the appearance of their teeth and gums (*p* < 0.008). On the other hand, they presented a relatively high frequency of flossing (*p* < 0.001) and brushing 3–5 times daily (*p* < 0.0001). In general, female adolescents had, to a certain extent, worse OH behaviours, perceptions and dietary habits than males did, and these findings were not in compliance with similar studies [52,53,54]. Adolescence is a period in which risky health behaviour, such as poor food choices, inadequate diet and inadequate maintenance of oral hygiene are often demonstrated. Additionally, adolescence represents an important period for the acceptance of positive health habits that could be prolonged into adulthood and last throughout life [21]. Adolescents can recognize the importance of OH from the aspects of their appearance, food consumption, and confidence. To maintain adequate OH throughout life, it is essential to adopt and develop good OH habits during the period of adolescence [21,55]. Oral diseases have a psychological, physical, and social consequences on adolescents’ lives. A recent systematic umbrella review reported that “dental caries, malocclusion, temporomandibular disorders, dental trauma, poor brushing, toothache, periodontal disease, and edentulism have a negative impact on OHRQoL. In addition, social determinants, such as demographic and socioeconomic factors, such as being an only child, growing up in a nuclear family, housing area and security, level of parental education, access to dental care and the performance of health promotion programs are directly proportional to OHRQoL and self-perception in adolescents” [56]. SES evaluation represents an important determinant from the aspects of adolescents’ behaviours, attitudes and conditions of OH. In the study, no statistically significant association was found between OH behaviours, perceptions, dietary habits and SES. Our findings were not in consistence with those of earlier investigations, where a high correlation between the level of SES and frequency of toothbrushing were determined [57,58]. Additionally, our findings were opposite to the findings of other national surveys regarding the association between SES and frequency of sweets consumption [49,50]. In contrast, other studies reported a high correlation between the level of SES and OH status, showing that adolescents with low SES had a significant negative impact on oral health behaviours and OH status in general later in life [23,49,56,59,60]. Disparities in caries experience, however, cannot be accounted for by SES-associated differences in brushing, flossing, sealant use, fluoride exposure, or the frequency of use of dental services [61]. School-based OH programmes might help promote OH [1]. “The techniques taught to students in schools in Western industrialized nations, such as the USA, Canada, Denmark, the UK and Sweden were related mainly to preventive dental measures and dental health guidance. In contrast, training schools in South Korea and Japan placed less emphasis on dental preventive measures and dental health guidance [62]”. The key provider of health promotion and preventive work in dental care is the dental hygienist”. The dental hygienist profession is one of the few in which the primary function of the practitioner is to prevent oral disease and to promote patients’ well-being [62,63]. In Bosnia and Herzegovina, only the School of Dental Medicine of the University of Sarajevo has a dental hygiene program that provides education and clinical training in dental hygiene. “The educators held a general respect and appreciation for the dental hygienist profession. They felt that dental hygienists’ skills ought to be more useful in orthodontics and preventive care than is customary today, including in tobacco prevention and smoking cessation as well as in dietary instruction among adults [60]. If indicated, these oral health programs should aim to change behaviours, attitudes and habits from the earliest school years.

## 5. Conclusions

- The OH behaviours, perceptions and dietary habits of adolescents in the Tuzla Canton were rather similar to those of their peers from other parts of the country.
- Over forty percent of the adolescents consumed sugary foods on a daily basis and visited the dental office only in the case of pain.
- More frequent dental visits and occasional toothaches might be correlated with significantly greater sugar intake in female adolescents than in male adolescents.
- No association between SES and oral health behaviours, perceptions and risk factors were detected. This could be attributed to equal access to public dental services for all residents in Bosnia and Herzegovina.
- In general, Bosnia and Herzegovina need an OH prevention program designed for high schools and dental hygiene programs that will be part of the regular education curriculum at all schools of dental medicine in the country. Although certain OH behaviours and dietary habits were at a satisfactory level, additional effort through continuous education should be made to improve individual OH status.

### Limitations of the Study

The decayed, missing and filled teeth (DMFT) index was not included used for the assessment of oral health status. As clinical variables were not included the results should be read with caution. Since the oral health status evaluation was based on participants’ self-reported assessments potential biases might be introduced.

## Figures and Tables

**Table 1:** Sociodemographic data.

		N	%
Sex	Male	123	40.2
	Female	183	59.8
Age	15 years	54	17.6
	16 years	76	24.8
	17 years	46	15.0
	18 years	130	42.5
	Mean	16.82	
	SD	1.16	
	Min.–Max.	15–18	
Socioeconomic status	Below average	132	43.1
	Average	140	45.8
	Above average	34	11.1
	Total	306	100.0

**Table 2:** Oral health behaviours and oral hygiene habits in relation to sex differences.

Variables	Gender		OR	χ^2^ Value	*p* Value
	Male	Female			
Dental visits within the past 12 months			3.099	16.877	0.005
Never	26 (21.1)	23 (12.6)			
Four times	15 (12.2)	41 (22.4)			
Reason for the last dental visit			1.115	6.498	0.09
Pain or problem with teeth or gums	53 (43.1)	74 (40.4)			
Frequency of toothbrushing			3.492	41.539	0.0001
Twice a day	87 (70.7)	107 (58.5)			
Three to five times a day	17 (13.8)	73 (39.9)			
Toothbrush usage			0		
Yes	123 (100)	183 (100)			
Toothpaste usage			0.223	0.08	0.643
Yes	122 (99.2)	182 (99.5)			
Fluoridated toothpaste usage			0.716	0.928	0.215
No	105 (85.4)	163 (89.1)			
Use of wooden toothpicks			1.658	4.462	0.023
No	68 (55.3)	123 (67.2)			
Use of plastic toothpicks			1.163	0.0257	0.362
No	101 (82.1)	146 (79.8)			
Use of dental floss			2.079	9.659	0.001
Yes	51 (41.5)	109 (59.6)			
Use of miswak			0.504	1.81	0.14
No	114 (92.7)	176 (96.2)			

Notes: Significant at *p* < 0.05. Chi square test was used.

**Table 3:** Oral health behaviours and oral hygiene habits according to socioeconomic status.

Variables	Socioeconomic Status, *n* (%)			χ^2^ Value	*p* Value
	Below Average	Average	Above Average		
Dental visits within the past 12 months				8.239	0.605
Never	26 (19.7)	20 (14.3)	3 (8.8)		
Four times	24 (18.2)	22 (15.7)	10 (29.4)		
Reason for the last dental visit				12.034	0.061
Pain or problem with teeth or gums	57 (43.2)	60 (42.9)	10 (29.4)		
Frequency of toothbrushing				14.906	0.0061
Twice a day	89 (67.4)	83 (59.3)	22 (64.7)		
Three to five times a day	32 (24.2)	47 (33.6)	11 (32.4)		
Toothbrush usage					
Yes	132 (100)	140 (100)	34 (100)		
Toothpaste usage				3.616	0.164
Yes	132 (100)	139 (99.3)	33 (97.1)		
Fluoridated toothpaste usage				0.594	0.743
No	114 (86.4)	123 (87.9)	31 (91.2)		
Use of wooden toothpicks				0.150	0.928
No	81 (61.4)	88 (62.9)	22 (64.7)		
Use of plastic toothpicks				0.047	0.977
No	107 (81.1)	113 (80.7)	27 (79.4)		
Dental floss usage				2.427	0.297
Yes	68 (51.5)	70 (50)	22 (64.7)		
Use of miswak				3.499	0.174
No	127 (96.2)	133 (95)	30 (88.2)		

Notes: *p* < 0.05. Chi square test was used.

**Table 4:** Perceptions of oral health in relation to sex differences.

Variables	Gender		OR	χ^2^ Value	*p* Value
	Male	Female			
Perceived state of teeth			1.051	4.942	0.423
Good	60 (48.8)	87 (47.5)			
Perceived state of gums			0.899	0.937	0.968
Good	44 (35.8)	70 (38.3)			
Toothache experience in the last 12 months			2.009	16.148	0.001
Occasionally	24 (19.5)	46 (25.1)			
Rarely	50 (40.7)	95 (51.9)			
Satisfaction with the appearance of teeth			1.909	9.742	0.008
Dissatisfied	35 (28.5)	79 (43.2)			
Avoiding smiling due to the state of teeth			0.471	1.918	0.383
Not avoiding	107 (87)	150 (82)			
Made fun of by other children about teeth			0.663	0.413	0.813
No	118 (95.9)	178 (97.3)			
Avoiding school due to toothache			0.735	0.522	0.77
No	119 (96.7)	175 (95.6)			
Difficulty biting food			0.638	1.072	0.585
No	115 (93.5)	165 (90.2)			
Difficulty chewing			1.06	0.129	0.937
No	116 (94.3)	172 (94)			

Notes: *p* < 0.05. Chi square test was used.

**Table 5:** Perceptions of oral health in relation to socioeconomic status.

Variables	Socioeconomic Status, *n* (%)			χ^2^ Value	*p* Value
	Below Average	Average	Above Average		
Perceived state of teeth				16.513	0.086
Good	69 (52.3)	63 (45)	15 (44.1)		
Perceived state of gums				14.969	0.144
Good	60 (45.5)	45 (32.1)	9 (26.5)		
Toothache experience in the last 12 months				6.574	0.362
Occasionally	35 (26.5)	30 (21.4)	5 (14.7)		
Rarely	63 (47.7)	67 (47.9)	15 (44.1)		
Satisfaction with appearance of teeth				3.038	0.552
Dissatisfied	49 (37.1)	53 (37.9)	12 (35.3)		
Avoiding smiling due to the state of teeth				8.274	0.082
Not avoiding smiling	103 (78)	122 (87.1)	32 (94.1)		
Made fun of by other children about teeth				2.505	0.644
No	129 (97.7)	134 (95.7)	33 (97.1)		
Avoiding school due to toothache				0.455	0.978
No	126 (95.5)	135 (96.4)	33 (97.1)		
Difficulty biting food				3.513	0.476
No	117 (88.6)	130 (92.9)	33 (97.1)		
Difficulty chewing				6.289	0.179
No	121 (91.7)	135 (96.4)	32 (94.1)		

Notes: *p* < 0.05. Chi square test was used.

**Table 6:** Comparison of dietary habits between male and female adolescents.

Variables	Frequency		Gender		OR	χ^2^ Value	*p* Value
			Male	Female			
Eating fruit	Every day	N	57	100	0.717	6.637	0.249
		%	46.3	54.6			
Eating cakes and biscuits	Every day	N	44	80	1.395	14.851	0.011
		%	35.8	43.7			
Drinking juices	Several times a week	N	38	55	1.04	0.721	0.982
		%	30.9	30.1			
Eating jam and honey	Several times a week	N	28	45	0.904	3.926	0.56
		%	22.8	24.6			
Consuming sugar gum	Every day	N	27	68	0.476	14.464	0.013
		%	22	37.2			
Eating sweets	Every day	N	41	91	1.978	12.946	0.024
		%	33.3	49.7			
Drinking milk with sugar	Several times a week	N	29	33	0.713	2.95	0.708
		%	23.6	18			
Drinking tea with sugar	Several times a week	N	41	48	0.711	6.273	0.281
		%	33.3	26.2			
Drinking coffee with sugar	Several times a week	N	28	20	2.402	13.077	0.023
		%	22.8	10.9			

Notes: *p* < 0.05. Chi square test was used.

**Table 7:** Dietary habits in relation to socioeconomic status.

Variables	Frequency	Socioeconomic Status, *n* (%)			χ^2^ Value	*p* Value
		Below Average	Average	Above Average		
Eats fruit	Every day	68 (51.5)	71 (50.7)	18 (52.9)	12.135	0.276
Eats biscuits and cake	Every day	52 (39.4)	64 (45.7)	8 (23.5)	16.940	0.076
Drinks juices	Several times a week	38 (28.8)	45 (32.1)	10 (29.4)	15.084	0.129
Eats jam and honey	Several times a week	31 (23.5)	35 (25)	7 (20.6)	3.953	0.949
Consumes sugar gum	Every day	43 (32.6)	44 (31.4)	8 (23.5)	12.249	0.269
Eats sweets	Every day	46 (34.8)	72 (51.4)	14 (41.2)	17.098	0.072
Drinks milk with sugar	Several times a week	19 (14.4)	35 (25)	8 (23.5)	10.317	0.413
Drinks tea with sugar	Several times a week	43 (32.6)	37 (26.4)	9 (26.5)	5.477	0.857
Drinks coffee with sugar	Several times a week	22 (16.7)	24 (17.1)	2 (5.9)	19.925	0.030

Notes: *p* < 0.05. Chi square test was used.

## Data Availability

The datasets generated and/or analysed during the current study are available from the corresponding author on reasonable request.

## Supplementary Materials

The following supporting information can be downloaded at: https://www.mdpi.com/article/10.3390/healthcare13111347/s1, WHO oral health surveys.
