# Supplementary material for: Assessment of the Oral Health Perceptions and Behaviours of Adolescents in Bosnia and Herzegovina: A Cross Sectional Study
Source: Healthcare (Basel). 2025 Jun 5;13(11):1347. doi: 10.3390/healthcare13111347 (PMC12155286; doi:10.3390/healthcare13111347)
Supplement: Supplementary file 1 [file healthcare-13-01347-s001.zip › WHO oral health surveys, basic methods.pdf]

Oral Health Surveys
Basic Methods

This manual WHO Oral Health Surveys – Basic Methods encourages countries to conduct standardized oral health surveys that are comparable internationally.

The manual provides guidelines for assessing the current oral health status of a population and the future needs for oral health intervention.

Oral diseases are among the most prevalent non-communicable diseases and major components of the global burden of disease.

The key risk factors are common to chronic and oral diseases. Guidelines are presented for risk factors assessment. The WHO approach can facilitate the incorporation of oral health into national health surveillance systems.

Surveillance of oral health over time and monitoring of programmes may provide decision-makers, public health planners, and health administrators with valuable information about the relevance and cost-effectiveness of public health intervention programmes.

World Health Organization

ISBN 978 92 4 154864 9

Created by WHO/Graphics

Oral Health Surveys Basic Methods

Oral Health Surveys
Basic Methods

Fifth Edition

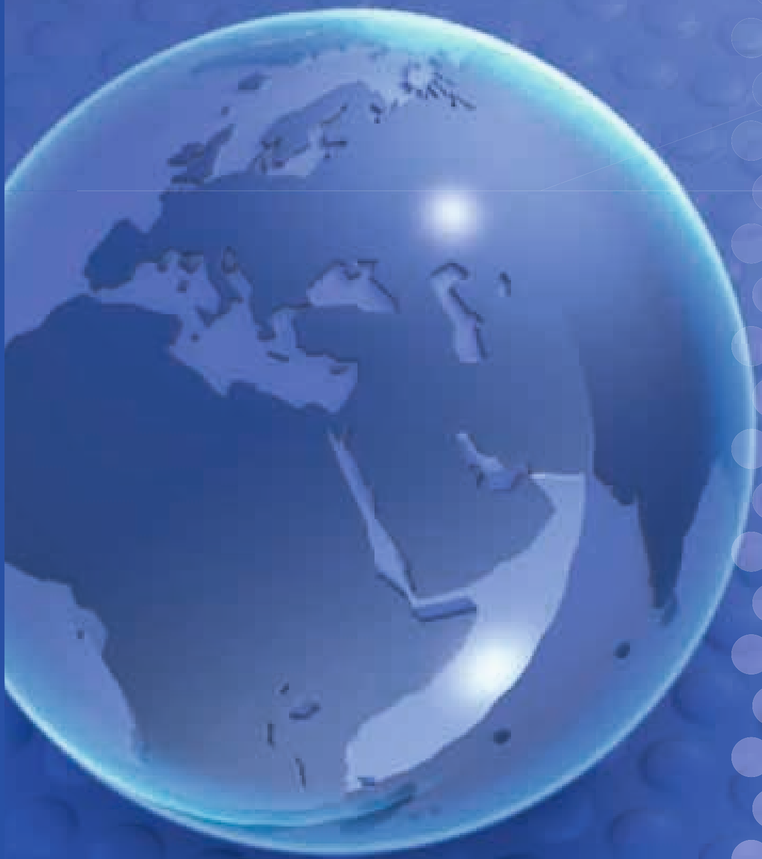

World Health Organization

Oral Health Surveys

Basic Methods

5th Edition

Oral
Health Surveys

Basic Methods

5th Edition

World Health
Organization

WHO Library Cataloguing-in-Publication Data

Oral health surveys: basic methods – 5th ed.
1. Oral health surveys. 2. Dental health. 3. Data collection. 4. Oral health surveillance. I. Petersen, Poul Erik. II. Baez, Ramon J. III. World Health Organization.

ISBN 978 92 4 154864 9 (NLM classification: WU 30)

© World Health Organization 2013

All rights reserved. Publications of the World Health Organization are available on the WHO web site (www.who.int) or can be purchased from WHO Press, World Health Organization, 20 Avenue Appia, 1211 Geneva 27, Switzerland (tel.: +41 22 791 3264; fax: +41 22 791 4857; e-mail: bookorders@who.int).

Requests for permission to reproduce or translate WHO publications – whether for sale or for non-commercial distribution – should be addressed to WHO Press through the WHO web site (www.who.int/about/licensing/copyright_form/en/index.html).

The designations employed and the presentation of the material in this publication do not imply the expression of any opinion whatsoever on the part of the World Health Organization concerning the legal status of any country, territory, city or area or of its authorities, or concerning the delimitation of its frontiers or boundaries. Dotted lines on maps represent approximate border lines for which there may not yet be full agreement.

The mention of specific companies or of certain manufacturers' products does not imply that they are endorsed or recommended by the World Health Organization in preference to others of a similar nature that are not mentioned. Errors and omissions excepted, the names of proprietary products are distinguished by initial capital letters.

All reasonable precautions have been taken by the World Health Organization to verify the information contained in this publication. However, the published material is being distributed without warranty of any kind, either expressed or implied. The responsibility for the interpretation and use of the material lies with the reader. In no event shall the World Health Organization be liable for damages arising from its use.

PRINTED IN FRANCE

Contents

Preface 1
Aims of the manual 2

Acknowledgements 3
Financial support 4

Introduction 5
Objectives 8
Structure of the manual 10

Section 1: Basic principles of clinical oral health surveys 11

1.1 Design of an oral health survey 13
1.1.1 Special characteristics of oral diseases 13
1.1.2 Index ages and age groups 14
1.1.3 Sample selection 16
1.1.4 Probability selection methods 16
1.1.4.1 Systematic sampling 17
1.1.4.2 Stratified sampling 17
1.1.4.3 Multistage cluster sampling 17
1.1.4.4 Probability proportional to size sampling 18
1.1.5 Pathfinder surveys 18
1.1.5.1 Subgroups 19
1.1.5.2 Number of subjects 20

1.2 Organizing the survey 22
1.2.1 Preparing a survey protocol 22
1.2.2 Obtaining approval from the authorities 23
1.2.3 Budgeting 23
1.2.4 Scheduling 23

1.2.5 Emergency intervention and referral 23
1.2.6 Courtesy reporting 24

1.3 Reliability and validity of data 25
1.3.1 Training and calibrating examiners 25
1.3.2 Duplicate examinations 26
1.3.3 Estimating reproducibility of recordings 27

1.4 Implementing the survey 29
1.4.1 General preparation 29
1.4.1.1 Contacts with persons in authority 29
1.4.1.2 Keeping a logbook 29
1.4.1.3 Preliminary exercise or pilot study 29
1.4.1.4 Sources of fluoride 30
1.4.2 Personnel and organization 30
1.4.2.1 Recording clerk 30
1.4.2.2 Organizing clerk 31
1.4.2.3 Daily review of assessment forms 31
1.4.3 Instruments and supplies 31
1.4.4 Infection control 32
1.4.5 Examination area 32
1.4.6 Examination position 33
1.4.7 Lighting 33
1.4.8 Table or platform 34
1.4.9 Seating of recording clerk 34
1.4.10 Supply of survey forms 34
1.4.11 Avoidance of crowding 34
1.4.12 Avoidance of noise 34

1.5 Assessment of oral health status 35
1.5.1 Standard forms 35
1.5.2 Standard codes 35
1.5.3 Oral health assessment form 36
1.5.4 Identification and general information sections of the form 38
1.5.5 Clinical examination 42
1.5.5.1 Dentition status 42
1.5.5.2 Periodontal status: Community Periodontal Index (CPI) modified 47
1.5.5.3 Loss of attachment 50
1.5.5.4 Enamel fluorosis 51
1.5.5.5 Dental erosion 52
1.5.5.6 Traumatic dental injuries 53
1.5.5.7 Oral mucosal lesions 53

1.5.5.8 Denture status 55
1.5.5.9 Intervention urgency 55

Section 2: Oral health self-assessment 57

2.1 Self-assessment of oral health and risks 59
2.1.1 Oral health information system 59
2.1.2 Self-assessment of oral health through use of questionnaires 59
2.1.2.1 Training of interviewers and supervision 61
2.1.3 Oral health risk indicators within the frame of STEPS 62

2.2 From surveys to surveillance 65

Section 3: Obtaining assistance from WHO 69

3.1 Pre-survey assistance 71

3.2 Post-survey assistance 73

3.3 Preparation of survey reports 77

References 81

Annex 1 WHO Oral Health Assessment Form for Adults, 2013 83

Annex 2 WHO Oral Health Assessment Form for Children, 2013 85

Annex 3 WHO Oral Health Assessment Form for Adults (by tooth surface), 2013 87

Annex 4 WHO Oral Health Assessment Form for Children (by tooth surface), 2013 89

Annex 5 WHO Record Form for Oral Manifestations in HIV/AIDS, 2013 91

Annex 6 Atlas of major oral diseases and conditions 93

Annex 7 WHO Oral Health Questionnaire for Adults, 2013 111

Annex 8 WHO Oral Health Questionnaire for Children, 2013 115

Annex 9 Standard tables generated from clinical survey data 119

Annex 10 Standard tables generated from STEPS questionnaire data 123

Preface

Basic oral health surveys provide a sound basis for assessing the current oral health status of a population and its future needs for oral health care. The World Health Organization (WHO) has a long tradition of epidemiological survey methodology, which includes a description of the diagnostic criteria that can be readily understood and applied in public health programmes worldwide. Guidelines have been elaborated for practical and economical sample designs suitable for recording the prevalence of oral diseases required for planning oral health programmes. In addition, WHO sets out clear principles for summarizing data and analyzing results.

The WHO manual *Oral Health Surveys – Basic Methods* has encouraged countries to conduct standardized oral health surveys that are comparable internationally. The WHO Global Oral Health Data Bank collates the data gathered through country surveys on the burden of oral disease and WHO recommended statistical analysis on key indicator age groups of children and adults. Regular conduct of oral health surveys in a number of countries has revealed important trends in oral health status, particularly among children. On one hand, in several high-income countries, the oral health of children has improved following the introduction and consolidation of oral disease prevention programmes. Rapidly increasing levels of oral disease, on the other hand, have been observed in a number of low- and middle-income countries in parallel with changes in living conditions and the increasing adoption of unhealthy lifestyles. In addition to social determinants, a number of behavioural risk factors influence oral health, such as a sugar-rich diet, use of tobacco, excessive consumption of alcohol and weak oral hygiene traditions. Limited availability and accessibility of oral health services and the lack of health promotion and effective preventive programmes are also associated with poor oral health.

Since the publication of the fourth edition, WHO has developed new tools for implementation of oral health surveys. In addition to guidelines for clinical examination of oral health status, instruments have been developed for surveillance of self-reported oral health and related risk factors. Because of the shared major risk factors of noncommunicable chronic and oral diseases, the

WHO STEPwise approach to Surveillance (STEPS) facilitates the inclusion of oral health in national surveillance systems for noncommunicable chronic diseases.

Aims of the manual

This manual aims to encourage national oral health survey planners to standardize measurements of oral diseases and conditions that are important for planning and evaluation of oral health programmes, as well as to ensure the comparability of data collected in a wide range of environments. It does this by applying the WHO global approach to chronic disease surveillance to an operational model for integration of oral health into chronic disease surveillance systems. Adoption of this approach will:

- encourage systematic reporting of data on oral diseases and conditions;
- ensure that the data collected are reliable and comparable within and between countries;
- encourage collection of data on self-reported oral health and risk factors consistent with STEPS framework; and
- provide an option to countries that have a special interest in collecting data on major oral conditions associated with the human immunodeficiency virus (HIV AIDS).

This manual is divided into three sections. **Section 1** provides background information for survey planners on the methods and approaches relevant to collection of clinical data on oral health status. **Section 2** covers self-reporting of oral health and the risk factors assessed by means of questionnaires. **Section 3** describes survey activity such as data analysis and survey reporting.

Acknowledgements

This manual was prepared by Dr P.E. Petersen, World Health Organization (WHO), and Dr R.J. Baez, University of Texas Health Science Center at San Antonio, Texas, USA.

WHO is grateful to Dr C. Scully, WHO Collaborating Centre for Oral Health-General Health, University of Bristol, Bristol, UK, and Dr M. Valderrama, Bogota, Colombia, for providing clinical photographs used in this manual to facilitate the identification of oral diseases.

Special thanks for technical advice are due to Dr E. D. Beltrán-Aguilar, Division of Oral Health, Centers for Disease Control and Prevention, Atlanta, Georgia, USA; Dr N. Jürgensen, WHO Collaborating Centre for Community Oral Health Programmes and Research, University of Copenhagen, Copenhagen, Denmark; Dr H. Ogawa, WHO Collaborating Centre for Translation of Oral Health Science, University of Niigata, Niigata, Japan; Dr AJ Rugg-Gunn, Newcastle University UK; Dr J. Stjernswärd, WHO Collaborating Centre for Education, Training and Research, Malmö University, Malmö, Sweden, and Dr D.M. O'Mullane and Dr H.P. Whelton, WHO Collaborating Centre for Oral Health Services Research, University of Cork, Cork, Ireland.

The survey tools have been field tested in several countries around the world. In particular, WHO thanks Dr U. Nyandindi, Ministry of Health, National School Health Programme, Dar es Salaam, the United Republic of Tanzania; Dr F. Kahabuka, WHO Collaborating Centre for Primary Oral Health Care, Planning and Research, Muhimbili University of Health and Allied Sciences, Dar es Salaam, the United Republic of Tanzania; Dr N. Razanamihaja, University of Majunga, Majunga, the Republic of Madagascar; Dr L. Rajab, University of Jordan, Amman, the Hashemite Kingdom of Jordan; Dr A. Naseeb, Ministry of Health, the Kingdom of Bahrain; Dr L. Zhu, WHO Collaborating Centre for Research and Training in Preventive Dentistry, Peking University, Beijing, the People's Republic of China; and Dr M.H. Khoshnevisan, Ministry of Health, Teheran, the Islamic Republic of Iran.

WHO acknowledges the support of the Academy of Dentistry International in preparing survey summary tables.

Financial support

The publication of this manual received kind support from the Philip Dear Foundation, the European section of the International College of Dentists. WHO is most grateful to this valuable contribution. The manual will facilitate education within dental public health and easy access to the website version of the manual will be of great help for survey implementation in developing countries.

The aim of this manual is to provide practical and robust tools for planning and surveillance of oral health intervention; in addition, the standardized approach makes the examination procedures useful in research. WHO is grateful for the funds kindly made available for publishing of this manual by the WHO Collaborating Centre for Translation of Oral Science, Niigata University, Niigata, Japan.

On-line version

The web-based version of the WHO *Oral health surveys – basic methods* is available from www.who.int/oral_health.

Introduction

The public health problems associated with oral disease are a serious burden on countries around the globe. The World Health Organization (WHO) has a long tradition of epidemiological survey methodology and surveillance in oral health. Since 1971, when the first edition of the WHO *Oral Health Surveys – Basic Methods* was published (*1*), many countries around the world have used the procedures described in the manual to produce information on current levels of disease and trends over time, as well as changes in prevalence and severity resulting from community programmes for prevention of oral disease (*1–4*). WHO regularly assesses the burden of oral disease from country data stored in the WHO Global Oral Health Data Bank (available through www.who.int/oral_health), and the public health importance of social determinants and behavioural risk factors has been recognized (*5–7*).

In order to undertake international comparisons, WHO encourages Member States to report information on disease levels using standardized methods. Past editions of this manual (*1–4*) presented a range of basic instruments and record forms for use in the collection of clinical data: standard criteria for recording clinical conditions were recommended, focusing on oral mucosal lesions and oral precancer/cancer, dentition status, enamel fluorosis, dental caries and dental treatment needs, developmental anomalies of the teeth, periodontal disease and treatment needs, as well as prosthetic status and needs. Special recommendations were made to ensure collection of high-quality data through clearly defined examination procedures, training and calibration of examiners for assessment of intra-/ inter-examiner consistency. The WHO basic oral health surveys have proved extremely useful in the evaluation of oral health status of populations, and for the development or adjustment of health systems and assessment of population-level programmes.

Surveys carried out over the past decade or so based on the fourth edition of *WHO Oral Health Surveys* (*4*) confirm that data on oral health status are important for surveillance of disease patterns. In addition to epidemiological information, however, oral health planners and decision-makers may require information about the risk factors associated with oral health, oral health-related quality of life, service coverage and utilization of oral health services,

intervention and care, administrative procedures, and quality of care and interventions (*8*).

For effective surveillance, WHO suggests that clinical oral health surveys should be conducted regularly every five to six years in the same community or setting. Surveillance underpins public health action by linking health policies and programmes to data. In other words, surveillance provides a means of ongoing (i.e. continuous or periodic) collection, analysis and interpretation of population data, and the timely dissemination of such data to health authorities or planners of public health programmes. Properly conducted surveillance programmes ensure that countries have the information they require for immediate action on disease control or to plan strategies to prevent disease and adverse health events in the future. In sum, the goal of health surveillance is to assist governments, health authorities and health professionals in formulating policies and programmes for prevention of disease and measurement of progress, impact and efficacy of efforts to control prevalent diseases and restore quality of life.

Information on the major common risk factors for chronic diseases is the key entry point for health authorities in planning health promotion and primary prevention programmes. Based on standardized survey instruments and previously agreed indicators, definitions, methods and sampling principles, WHO has developed important new tools for assessment of chronic disease and risk factors that are also highly relevant to oral health. These surveillance tools can be used to collect essential data on self-reported oral health, oral health behaviour, use of available oral health services, oral health-related quality of life, and the socio-environmental risk factors for oral health. The WHO tools have been developed for comprehensive surveillance systems that can be adapted to local needs and resources.

The fifth edition of WHO *Oral Health Surveys – Basic Methods* makes the following recommendations for oral health surveys.

- Dentition status should be recorded in compliance with the recommendations given in previous editions of this manual. This will ensure that the prevalence and incidence of dental caries continues to be recorded using robust criteria relevant to public health. It is noted that detailed classifications of dental caries have been suggested for application in clinical dental practice, where specialized equipment may be available for more detailed assessment of the degree of dental tissue involvement. Recording of specific dental treatment needs for individual teeth is no longer recommended.
- Recording of periodontal status by sextants or index teeth has been modified to include assessment of gingival bleeding and recording of pocket scores for all teeth present. The rationale for this recommendation is that since the dentition status is assessed in all teeth present, the

same meticulous examination should be conducted to assess the periodontal status of the same teeth. Presence of calculus is not recorded because it is not a disease per se.

- The recommendation not to probe pocket depth in children under 15 years of age remains unchanged. Loss of attachment should be recorded using index teeth except in children under 15 years of age, who are again excluded for this measure.
- The manual continues to recommend recording presence of enamel fluorosis because although it is not considered a disease it assists countries in establishing baseline data prior to or when monitoring community prevention programmes that include fluoride for prevention of dental caries. Calculation of the community fluorosis index (CFI) is recommended to determine whether fluorosis is a public health concern. The CFI allows identification of objectionable fluorosis in community groups. Clinical data on fluorosis must be complemented with fluoride exposure studies.
- Based on recent reports of the high incidence of dental erosion related to frequent consumption of highly acidic beverages, sucking or chewing of highly acidic fruit-flavoured sweets and gums, and other culturally influenced diets, a special section has been included in the data collection form for recording loss of tooth substance due to erosion as well as the number of teeth involved.
- Orodental trauma includes injury to the mouth, including the teeth, lips, gingivae, tongue and jaw bones. The most common dental trauma involves fracture or loss of a tooth. Orodental injury can result in disfigurement and dysfunction, greatly diminishing quality of life and contributing to social and economic burdens. Thus a section for recording dental trauma and the number of teeth involved has been added in the data collection form.
- Recording of presence of fixed or removable dentures has been included in the adult assessment form as an indication of access to health services. However, the quality or function of the prosthetic device is not assessed.
- The survey includes examination of the oral mucosa and recording of extraoral conditions and their location using standardized coding. In addition to the basic record forms, a special form has been developed for recording the most common oral lesions occurring in human immunodeficiency virus (HIV) infection and acquired immunodeficiency syndrome (AIDS).
- The section on intervention urgency specifies the recommended level of treatment in response to acute oral problems and the need for immediate referral to special care. This may also include preventive care or routine dental care required for large carious lesions with or without accompanying pain or evident infection. Urgent intervention may also

be needed in cases of reported pain and obvious signs of severe infection, e.g. due to an abscess of dental or periodontal origin.

Basic questionnaires have been designed for adults and children separately, and they may be used for the collection of information on self-assessment of oral health status, oral health habits, risk behaviour, quality of life and social position. Risk factor analysis is central to the development or adjustment of oral health promotion programmes. The oral health effect of risk factors can be estimated by merging clinical and socio-behavioural data.

Objectives of the manual

The objectives of this manual are to provide essential information on the organization and planning of oral health surveys carried out for assessment of the oral health status of a population, and the collection of data on self-assessment of oral health and socio-behavioural risk factors. The objectives of the manual are as follows:

1. **Provision of information relevant to planning a survey on oral health status. This includes:**
   - design of basic oral health surveys
   - guidelines on practical sample designs suitable for assessing oral diseases
   - organizing the oral health survey
   - implementing the oral health survey
   - assessment forms and description of diagnostic criteria and codes
   - recommendations to ensure reliability of data
   - guidelines on post-survey actions, including essential data to be reported and the appropriate reporting format
   - provision of a plan for recording and reference to WHO guidelines for implementing epidemiological studies of HIV/AIDS-related oral diseases.
2. **Provision of information relevant to planning a survey on self-assessment of oral health and risk factors**

The WHO STEPwise approach to Surveillance (STEPS) is a sequential process (**Fig. 1**) (*9*): it starts with the compilation of key information on risk factors and self-reported health using a questionnaire, and then moves on to simple physical measurements, followed by more complex measurements for biochemical analysis. The instruments may include core, expanded and optional data. STEPS emphasizes that fewer good-quality data are more valuable than large quantities of poor-quality data. By using the same standardized questions and protocols, countries can use the information produced by

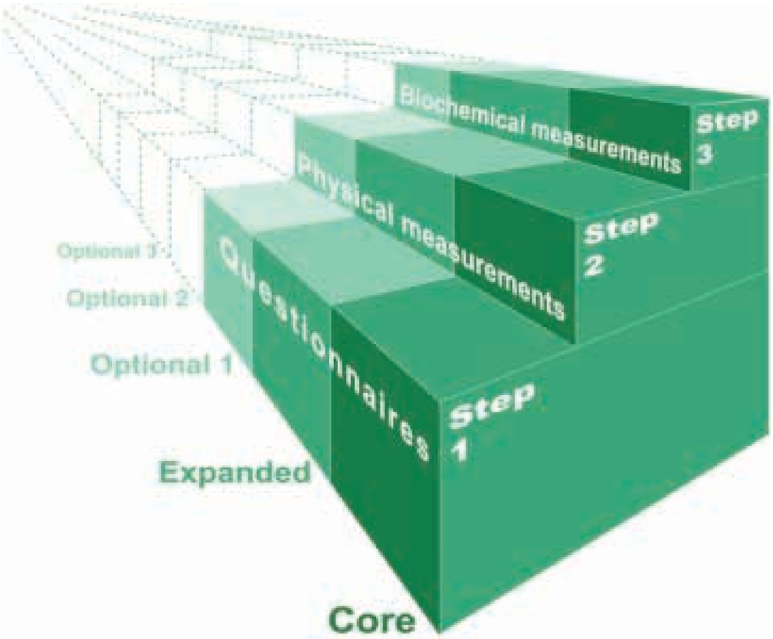

Fig. 1. Framework for the WHO STEPwise approach to chronic disease surveillance (*9*).

STEPS both for assessment of within-country trends as well for comparisons across countries. The approach encourages the collection of small amounts of useful information on a regular and continuing basis.

STEPwise application of oral health principles has three steps:

- Step 1: the acquisition of information on self-assessment of oral conditions, oral health practices, measurements of diet, tobacco use and alcohol consumption, quality of life, and social position, all of which are based on standard WHO definitions. This may include data on general health factors that are of importance to oral health conditions, e.g. height, weight and waist circumference as indicators of nutritional status, underweight or obesity, experience of diabetes and markers of HIV infection.
- Step 2: the clinical data collected in Step 2 add to those obtained in Step 1. The implementation of Steps 1 and 2 have relevance to most countries.
- Step 3: this comprises information obtained from biochemical analysis, e.g. collection of saliva to study its buffering capacity or for microbial assessment (e.g. *Streptococcus mutans)*. However, WHO does not

recommend advanced oral health measurements for countries with limited resources.

Oral health surveillance becomes possible when data on oral health status and risk factors are collected systematically and on a regular basis. Countries may choose to collect Step 1 or Step 2 data or both for planning and evaluation of oral health intervention.

Structure of this manual

Section 1 outlines the principles of clinical oral health surveys as recommended by WHO. These include guidance for conducting the survey, the diagnostic criteria, reliability of data and selection of participants. The recommended WHO oral health assessment forms for recording the clinical findings are provided as annexes to this manual. The forms give the essential elements of the survey report and examples of tables that should be prepared from statistical analysis of the data that have been collected are also provided as separate annexes. Section 2 explains the approach of WHO to development of integrated oral health information systems. The risk factor model, which provides the conceptual framework for inclusion of self-reported oral health and risk factors within STEPS, is further detailed in this section. Additional measurements such as diabetes testing, and height and weight for calculation of the body mass index (BMI) may be included. Section 3 describes analysis of data and reporting of the results; in addition, this section provides information on the assistance available from WHO.

Section 1: Basic principles of clinical oral health surveys

1.1 Design of an oral health survey

Conduct of population surveys to determine oral health status and the need for intervention in communities is an essential part of the duties of chief dental officers and other administrators responsible for oral health programmes. Where there is no national or regional dental officer specifically responsible for oral health activities, a member of a dental society or staff of training institutions for oral health personnel may undertake regular epidemiological surveys of oral health conditions.

The methods described in this manual can be used to estimate:

- the distribution and severity of major oral diseases and conditions;
- the extent to which oral health programmes match the need for care of population groups and the need for community-oriented disease prevention and health promotion; and
- the nature and urgency of the oral health intervention(s) required.

If the survey includes expanded variables, the role of social determinants of oral health and common modifiable risk factors such as personal hygiene, diet, use of tobacco, excessive alcohol consumption, use of oral health services, and finally oral health-related quality of life of the population may also be assessed.

1.1.1 Special characteristics of oral diseases

The particular epidemiology of oral diseases has permitted development of an approach to sample design and survey planning for the most common oral diseases. The special considerations concerning the two major oral diseases – dental caries and periodontal disease – are as follows.

- The diseases are strongly age-related.
- The diseases exist in all populations, varying only in prevalence and severity.
- One of the diseases, dental caries, is irreversible (at the cavitation level used in the methods described here), and thus information on current status provides data not only on the amount of disease present but also on previous disease experience.

- There is extensive documentation on variation in profiles of oral disease among population groups with different socioeconomic levels, behavioural characteristics and environmental conditions.
- Standardized measurement in each subject requires several observations to be made; in the case of caries for each tooth and for the assessment of periodontal status for each tooth present in the mouth, with the exception of loss of attachment, for which the six sextants are used.

1.1.2 Index ages and age groups

The following ages and age groups are recommended for population surveys.

5 years

Where it is practicable and feasible, children should be examined between their fifth and sixth birthdays. This age is of interest in relation to caries levels in the primary dentition, which may exhibit changes over a shorter timespan than in the permanent dentition at other index ages. In some countries, 5 years is also the age at which children begin primary school. In countries where school entry is deferred to 6 or 7 years of age, these ages can be used, although the mean age should be reported with the results. In these older age groups, missing primary incisors should not be scored as missing because of the difficulty in differentiating between normally exfoliated primary incisors and those lost because of caries or trauma.

12 years

This age is especially important as it is generally the age at which children leave primary school. Therefore in many countries, it is the last age at which a reliable sample may be obtained easily through the school system. Also, it is likely that by this age all the permanent teeth except the third molars will have erupted. For these reasons, age 12 years has been chosen as the global indicator age group for international comparisons and surveillance of disease trends.

In some countries, however, many school-age children do not attend school. In these circumstances, an attempt should be made to survey two to three groups of non-attendees from different areas to compare their oral health status with that of children still attending school.

15 years

By 15 years, the permanent teeth have been exposed to the oral environment for three to nine years. The assessment of caries prevalence in adolescents may

therefore be relevant. The age group of 15–19 years is also important in the assessment of periodontal disease in adolescents. In countries where it is difficult to obtain reliable samples from this age group, it is customary to examine individuals in two to three areas only, in the capital city or another large town and in one rural area.

35–44 years (mean = 40 years)

This age group is the standard age group for surveillance of oral health conditions in adults. By using data for this age group, planners and decision-makers can assess the full effect of dental caries, the level of severe periodontal involvement, and the general effects of oral health care provided. Sampling adult subjects is often difficult. Samples can, however, be drawn from organized groups, such as office or factory workers. Use may also be made of readily accessible groups, e.g. at a market, to obtain a reasonably representative sample in situations where truly representative sampling is not feasible. Care must be taken to avoid obvious selection bias, such as sampling patients at medical care facilities.

65–74 years (mean = 70 years)

The age group of 65–74 years has become more important with the changes in the age distribution of populations and the worldwide increase in lifespan. In this age group, it is possible to estimate the manifestation of oral disease from a life course perspective. Data for this group are needed both for planning appropriate interventions for older people and for assessment of the ultimate effect of oral health programmes in a population. Obtaining a sample of and examining representative members of this age group is often not as difficult as for the younger age groups, as older people are more likely to be in or near their homes, in senior clubs, day centres or institutions in the day time. Nevertheless, care should be taken to sample adequately both housebound and active members of this age group.

The above mentioned index ages or age groups are recommended for nationwide surveys of the general population. However, countries may have a special interest in studying conditions that may affect the oral health of children of younger ages and special adult groups or older people. Countries planning to engage in such efforts are encouraged to consider the usefulness and reliability of the data that will be produced and the feasibility of the study, as well as the validity of results and their applicability from a public health perspective. Where the study design or examination criteria do not follow the methods suggested in this manual, studies cannot be endorsed by WHO and the content of the reports produced will be the responsibility of the country undertaking this exercise.

1.1.3 Sample selection

Prior to sample selection, public health administrators and survey planners need to decide whether the survey will be carried out at local, regional or national level, the variables that will be examined in the survey and the age groups to be included. It is important to take these factors into consideration especially later in the process, i.e. when making inferences from the results of the survey to the population of the region or the country.

There are various techniques for estimating the sample size of a population for an oral health survey. The two major methods are probability and non-probability sampling, depending on whether there is a known probability of subjects to be selected for the survey. Depending on resources available and technical conditions, probability sampling can be executed in a number of ways including simple random sampling, systematic sampling, stratified cluster random sampling, probability proportional to size, as well as other variations. There are several statistical texts (*10, 11*) that describe the available sampling techniques in detail, including determination of sample size, precision and power. Investigators are encouraged to consult these texts and obtain advice from statisticians as a preliminary step in survey design. In some settings, investigators may have access to an expert in health statistics for statistical guidance on designing a basic oral health survey.

1.1.4 Probability selection methods

With a probability sample, each individual in the study population has a known, non-zero chance of being included in the sample. With this design, selection biases may be avoided provided that the response rate is high, and statistical theory can be used to derive inferences of the survey estimators. A critical factor for any form of probability selection is the existence of a sampling frame from which the sample elements can be selected. In other words, a list of the members of the population to be included in the survey must be available. Random sampling techniques require either numbering each individual in the entire group, mixing the numbers and – much like a lottery – drawing the desired number of persons to be examined or using a table of random numbers.

Questions often arise as to why the population to be studied is not selected using probability selection methods. This is most likely due to the unavailability of the requisite information for selecting the population groups to be included in the study, uncertainty as to which probability selection method to use, lack of full understanding of the various alternatives, and the difficulty in complying with such a precise method.

Simple random sampling is not widely used, a stratified mode is preferred to ensure inclusion of important subgroups. A number of techniques have

been developed that can be used for selecting survey samples representative of the target population; among these, the techniques most commonly used are systematic sampling, stratified random sampling, multistage cluster sampling and probability proportional to size sampling.

1.1.4.1 Systematic sampling

Systematic sampling involves selecting at random the first person from a list and then taking every x^{th} number or element (person) until the desired total of individuals has been selected. This number depends on the desired number of persons to be examined and the total number available as per a list pertaining to a particular age group. This information is necessary so that the sampling fraction can be calculated and used to identify the number (person) on the list and systematically choose every x^{th} person until the total number desired has been selected. If, for example, there were 1800 older people in an institution and the desired sample size was 200, the resulting sampling fraction would be 9. A random number is then chosen from between 0 and 9 to select the first person and then every ninth person listed is selected until the total needed for examination is obtained. Thus in systematic sampling, every person in the population listed has the same odds of being selected for the examination.

1.1.4.2 Stratified sampling

When conducting surveys in a population, it is important to record the type of location where the participants live. Generally, three types of community descriptors are used: urban, periurban and rural. Other information that may be available about the population to be studied can also be used to classify the population into groups and subgroups. This is the fundamental nature of stratification. Thus, the population can be classified into subgroups or strata, following which separate samples from each stratum are selected. If a simple random sample is selected from each of these strata, the total sample is called a stratified random sample. If the composition of the population is known with respect to a number of specified variables, e.g. age, sex and ethnic group, the sample can be selected to conform to this composition. Stratification is an appropriate technique for obtaining separate estimates for population parameters for each subgroup. Proper stratified sampling requires identification of every enumeration unit by stratum in advance of the sampling. If such information is not readily available, the method is not feasible.

1.1.4.3 Multistage cluster sampling

In most surveys, the population covered is composed of groups; each of these groups can be designated as strata and separate samples are selected from

each group. Groups can also be designated as clusters, and in this case a sample of clusters is first included in the survey design. When all the persons in the selected clusters will be included in the sample, the method is known as cluster sampling; but if only a sample of persons is taken from each selected cluster, the method is called two-stage sampling. The method can also be designated as three-stage sampling – or simply multistage sampling – depending on whether the sampling design requires multiple levels. A typical example is: selection of the first large cluster in a country, e.g. a state or department, then selection of samples of schools (second-level cluster), followed by samples of classrooms (third-level cluster) and finally samples of students. Multicluster sampling may lead to loss in precision, although it is relatively economical compared with other methods. However, unless a sufficiently large sample size can be ensured, the use of cluster sampling is not the best approach.

1.1.4.4 Probability proportional to size sampling

In this method of sample selection, the probability of a person being sampled is proportional to the number of persons within a particular group. This permits heavier sampling in subgroups with few members so as to provide acceptable estimates, not only for the population as a whole but also for each of its subgroups. Levels of school enrolment may differ between communities, and even within the same community school, classes may have different numbers of students. However, selection of schools can be conducted with probability proportional to size design. To be able to use this method, lists must be available of all schools or institutions enrolling individuals of the target age group. In addition, the total number of persons in each specific group needs to be known.

It is important to know the gender distribution within each age group to be studied. Based on knowledge of the total number of persons enrolled and the number of selections to be made, systematic sampling can be used to obtain a sample without replacement, i.e. by avoiding the possibility of a school or class being selected more than once. It is also important to know the number of selections to be made so that a sample interval can be calculated and the total number of units or individuals estimated.

1.1.5 Pathfinder surveys

Where there is no opportunity to conduct probability sample size estimates, a non-probability sampling method is applied. The particular factors associated with the most common oral diseases and the extensive experience gained in oral epidemiology over the past 35 years have allowed a practical, economic survey sampling method to be defined, the so-called pathfinder method.

The pathfinder survey method is a stratified cluster sampling technique that aims to include the most important population subgroups likely to have different disease levels. It also advises on appropriate numbers of subjects in specific index age groups in any one location. In this way, reliable and clinically relevant information for surveillance can be obtained at minimal expense.

The method is suitable for obtaining the following information:

- overall prevalence of the major oral diseases and conditions affecting the population;
- variations in disease levels and severity in subgroups of the population. This enables identification of groups with special intervention needs; and
- age profiles of oral diseases in the population to enable assessment of need for intervention in different age groups, to provide information about severity and progression of disease, and to assess whether the levels of a disease are increasing or decreasing.

Pathfinder surveys can be either pilot or national-level systematic surveys, depending on the number and type of sampling sites to be included and the age groups included.

A pilot survey is one that includes only the most important subgroups in the population and only one or two index ages, usually 12 years and one other age group. Such a survey provides the minimum amount of data needed to commence planning. Additional data should then be collected to provide a reliable baseline for the implementation and monitoring of oral health programmes.

A comprehensive systematic survey incorporates sufficient examination sites to cover all important subgroups of the population (i.e. groups with different disease levels or intervention needs), and at least three of the age groups or index ages recommended for examination by WHO. This survey design is suitable for collection of data for planning purposes and monitoring of oral health programmes in all countries regardless of the level of disease, availability of resources or complexity of care. In a large country with many geographical and population subdivisions and a complex health system structure, a larger number of sampling sites will be needed. The basic principle of using index ages and standard samples in each site within a stratified approach, however, remains valid. The method now described is recommended as a general guideline for basic oral health surveys for the planning, health surveillance and evaluation of oral health programmes.

1.5.1.1 Subgroups

The number and distribution of sampling sites depend upon the specific objectives of the study. Sampling sites are usually chosen to provide

information on population groups likely to have different levels of oral disease. The sampling is usually based on the administrative divisions of a country, i.e. the capital city, main urban centres, and small towns and rural areas. In countries where there are different geophysical areas, it is customary to include at least one sampling site from each area type.

If there are several distinct ethnic groups in the population with known or suspected differences in levels of oral disease, it may be necessary to include separate samples of each of these groups in the main subdivisions for the survey. Thus, maximum use should be made of available knowledge about variations between the different groups in order to limit the number of additional subsamples needed. Once the different subgroups to be included is decided, random sampling of subjects within the groups is desirable.

Assistance of local health administrators can be very useful when making the final decision about which population subgroups are significant for the study and should be represented in the final sample. For a national pathfinder survey, between 10 and 15 sampling sites are usually sufficient. If, however, there are many large urban centres in the country, it may be necessary to locate several additional sampling sites in at least two of these cities.

1.1.5.2 Number of subjects

The number of subjects to be included in the oral health survey depends on the technique used for conducting sample size estimates, scope of the survey, the precision with which inferences will be made, and resources available. In the pathfinder sampling method, the number of subjects to be examined in each index age group ranges from a minimum of 25 to 50 for each sampling site, depending on the expected prevalence and severity of oral disease. For example, a sample design for a national pathfinder survey for each index age or age group can be calculated as follows:

Urban:	4 sites in the capital city or metropolitan area	(4 × 25 = 100)
	2 sites in each of 2 large towns	(2 × 2 × 25 = 100)
Rural:	1 site in each of 4 villages in different regions	(4 × 25 = 100)
Total for one index age or age group:		12 sites × 25 subjects = 300

If this cluster distribution will be applied to four index ages in the population under study:

Total sample: 4 × 300 = 1200

Such a sample design permits identification of significant differences between urban and rural groups and, in most situations, between different socioeconomic groups in the capital city or other large towns. Areas where the

disease prevalence is either much higher or much lower than the national average may also be identified from the results of such a survey. It is to be noted that a sample of 25 subjects, with approximately equal numbers of females and males, is sufficient only in populations where dental caries and periodontal disease levels are estimated to be low or very low.

In populations where the disease level is known to be high, e.g. the percentage of caries-free 12-year-olds is 20% or lower, the standard size for each sample should be about 50 subjects and the total sample size should therefore increase to about 600 subjects for each age group. If the level of dental caries in the population is not known, it will be necessary to estimate the level of disease before starting a survey. A rapid and effective way of estimating the prevalence of dental caries in a population is by classifying a group of subjects as caries-free or not caries-free. It should be possible, for example, to examine two or three classes or year groups of 12-year-olds from different socioeconomic levels, in two to three local, easily accessible schools where the widest possible differences in disease prevalence may be expected. If more than 50% of the children in a class are caries-free, the caries prevalence is low, and if less than 20% are caries-free, the prevalence is high. This estimate of prevalence may then be used as a guide to decide the standard sample size when completing the study protocol.

1.2 Organizing the survey

1.2.1 Preparing a survey protocol

It is important to prepare a written protocol for the survey, and this should contain the following information:

1. purpose and main objectives of the survey
2. description of the type of information to be collected and of the methods to be used
3. description of design and sampling methods to be used
4. personnel and physical arrangements
5. training and calibration of examiners
6. statistical methods to be used in analysing the data
7. dissemination of findings
8. provisional budget
9. provisional timescale of the main activities and the responsible staff.

1.2.2 Obtaining approval from the authorities

Permission to examine population groups must be obtained from the relevant local, regional or national authority. If schoolchildren are to be examined, for example, the school authorities should be approached, the purpose of the survey explained and their approval obtained. In some instances, written permission from parents will need to be obtained before children can be examined. It is the responsibility of the local organizer of the survey to ascertain local practices regarding consent. The survey organizer should also notify the health authorities, since it may be necessary to time the survey to fit in with other health-related activities. This applies particularly when adult populations are to be surveyed.

It is important to provide dental professionals and oral health administrators in the area with details of the survey. Local dental society officers and dental practitioners can often help in gaining the cooperation of the community for the survey, and of any of their patients who may be included in the sample.

1.2.3 Budgeting

A budget for the survey should be prepared, listing the resources required, including personnel to carry out the survey. Basic oral health surveys can often be conducted as part of the duties of chief dental officers or dental public health workers, and they may be undertaken with only minor additions to existing budgets.

1.2.4 Scheduling

The preparation of a schedule for data collection is an important aspect of survey planning. If this is not done, examining personnel may waste time waiting for subjects to arrive, or be otherwise unnecessarily delayed.

The planner can estimate from a preliminary survey, or from previous experience, how much time, on average, each examination will take. As a guideline, a basic examination of a child usually takes about 10 minutes, while a complete examination of an adult may take between 15 and 20 minutes. Daily and weekly schedules can then be prepared. These should be made available to survey personnel, as well as to school and health authorities. The schedules should allow for some flexibility, so that unexpected delays do not cause major upsets in the survey timetable.

It is important for survey examiners to make reliable observations and consistent judgements. Since fatigue contributes significantly to inaccuracy and inconsistency, the schedule should not be too demanding. For example, if 25–30 children each in several classes/schools are to be examined by one examiner, the planned schedule should include time for:

1. introducing the examining team to the school director and class teachers concerned;
2. choosing an appropriate place to carry out the examinations in each school, and setting up equipment;
3. examining one class of 12-year-olds;
4. providing a brief oral report to the school director; and
5. travelling to the next school.

Planning an appropriate schedule is also important for surveys of adult populations to be carried out at, for example, a workplace or in a marketplace. Older people may be examined in nursing homes or in senior clubs or in their own homes.

1.2.5 Emergency intervention and referral

If a life-threatening condition or a condition that requires immediate attention is detected during the examination, it is the responsibility of the examiner or

team leader to ensure that referral to an appropriate care facility is made. A list of referral facilities and addresses should therefore be prepared before the survey. Examiners conducting the survey should not be encouraged to provide emergency care during the course of the oral examinations.

1.2.6 Courtesy reporting

It is appropriate, and often essential, to report the survey findings to local authorities. The report may be a simple summary of the number of subjects examined and the general observations of the examiner. This can usually be delivered personally, immediately after completion of the survey. Courtesy reporting should also be conducted for parents of participating children or for adults participating in the survey. Advice to parents/persons should be general so as to avoid conflicts in advice given by the survey team and local dental practitioners. Children and adults participating in the survey will benefit from knowing whether they require any dental attention; this form of courtesy reporting may help address any relevant oral health-care needs of the participants, such as improvement in oral hygiene practices, modification of habits and unhealthy lifestyles (risks), provision of routine, prompt or urgent oral intervention for an oral disease, pain or infection, or oral manifestation of a systemic disease that would require comprehensive evaluation by a dentist or physician. A full technical report will require more time to prepare, but should be sent as soon as it is complete.

1.3 Reliability and validity of data

1.3.1 Training and calibrating examiners

Although examiners may differ in their assessment of the oral health status of individuals, there should be close agreement between assessments in population groups (*12*). When an epidemiological survey is undertaken by a team, it is essential that the participating examiners are trained to make consistent clinical judgements. There are two main reasons for variability in clinical scoring:

- inconsistency in scoring different levels of an oral disease; and
- physical and psychological factors related to the examiner, such as fatigue, fluctuations in interest in the study, and differences in visual acuity and tactile sense.

All these affect the judgement of examiners from time to time and to different degrees. The objectives of standardization and calibration therefore are to ensure uniform interpretation, understanding and application by all examiners of the criteria and codes for the various diseases and conditions to be observed and recorded, and that each examiner can examine consistently.

When the survey will be conducted by a group of examiners, it is recommended that an experienced epidemiologist who is trained in accordance with the recommended methods for basic oral health surveys should be appointed to act as a validator for the survey team. The calibrator should examine at least 25 subjects who will also be examined by each member of the survey team.

Assistance in training and calibration may be available from WHO. Training in the use of the criteria generally takes two days with a further two to three days required for calibration. Additional time may be needed depending on the number of examiners requiring training and the number of indices that will be used in a survey. An interval of at least a few days is desirable between training and calibration to allow the examiners time to assimilate their knowledge of the indices and to practise the procedures.

It is necessary to assess the consistency of each individual examiner (intra-examiner reproducibility) and also the variations between examiners

(inter-examiner reproducibility). Each examiner should first practise the examination on a group of 10 subjects. Every examiner should then independently examine the same group of 20 or more subjects and compare his or her findings with those of the other examiners in the team. Where there are major discrepancies, the subjects should be re-examined so that inter-examiner differences can be reviewed and resolved by group discussion. It is essential that a group of examiners should be able to examine with reasonable consistency, using a common standard. In general, the consistency level for most assessments should be in the range of 85–95% (*12*). If the assessments made by certain examiners are consistently significantly different from the majority despite attempts to correct their performance, they should be excluded from the survey team. It should be made clear to all potential examiners before the examinations begin that the ability to make standardized assessments is not a measure of their clinical skill.

If all members of the survey team cannot make assessments in a consistent manner, regional or group variations in disease prevalence or severity may be missed or wrongly interpreted. Since there will always be some variation between examiners, it is advisable that, in the actual survey, they should all examine similar proportions of each major subgroup of the sample population.

When only one examiner is involved and an experienced trainer is not available, the examiner should first practise the examination on a group of 10 subjects with a wide range of levels of disease conditions. The examiner should then determine how consistently he or she can apply the diagnostic criteria by examining a group of about 25 subjects twice, ideally on successive days, or with a time interval of at least 30 minutes between examinations. These subjects should be pre-selected so that they collectively represent the full range of conditions expected to be assessed in the actual survey. By comparing the results of the two examinations, the examiner can obtain an estimate of the extent and nature of their diagnostic variability. If the variability is large, the examiner should review the interpretation of the criteria and conduct additional examinations until acceptable consistency is achieved.

1.3.2 Duplicate examinations

Examiners may change the way they apply diagnostic criteria during the course of a series of assessments. To allow detection and correction of this tendency, it is advisable for each examiner to perform duplicate examinations on 5–10% of the sample (no fewer than 25 subjects) in the actual survey. The most convenient age groups for duplicate examinations are likely to be the 12 and 15 years age groups because of ease of access. If the survey includes adult age groups, duplicate examinations can be coordinated to be performed in these individuals. As far as possible, the examiner should not be able to

identify the subjects who are re-examined, or know that a subject has been examined previously, since this information may affect the thoroughness or quality of the duplicate examination. The recorder, or perhaps a local school-teacher, should be requested to arrange for the re-examination of the subjects during the course of the survey. Duplicate examinations should be conducted at the start of the survey (i.e. immediately after calibration), about half-way through and at the end of the survey.

1.3.3 Estimating reproducibility of recordings

Inter-and intra-examiner consistency can be assessed in a number of ways, the simplest being the percentage of agreement between scores, i.e. the percentage of subjects allocated the same score by two examiners. In the case of dental caries, however, if the prevalence of disease is low, this method does not provide an accurate assessment of reproducibility. A more reliable way of assessing overall agreement between examiners is the kappa statistic (*12–14*). The kappa statistic relates the actual measure of agreement with the degree of agreement which would have occurred by chance. The kappa score can be calculated using a 2 × 2 table (**Table 1**).

When applied to recording dental caries at the tooth level:

a = proportion of teeth both examiners consider to be sound
b = proportion of teeth examiner 1 considers to be sound and examiner 2 considers to be carious
c = proportion of teeth examiner 1 considers to be carious and examiner 2 considers to be sound
d = proportion of teeth both examiners consider to be carious.

The Kappa statistic formula is:

$$\varkappa = \frac{P_o - P_e}{1 - P_e} \quad (1)$$

Table 1. Calculation of the kappa score in examining for dental caries

**Examiner 2**	**Examiner 1**		
	Sound	*Carious*	**Total**
Sound	a	c	a + c
Carious	b	d	b + d
**Total**	a + b	c + d	a + b + c + d

where:

P_0 = proportion of observed agreement (a + d);

P_e = proportion of agreement that could be expected by chance (a + c) × (a + b) for sound teeth, and (b + d) × (c + d) for carious teeth. Hence

$$P_e = \frac{(a+c)\times(a+b)+(b+d)\times(c+d)}{(a+b+c+d)^2} \qquad (2)$$

The Kappa statistic is interpreted as follows (*12*):

<0.20 poor agreement
0.21–0.40 fair agreement
0.41–0.60 moderate agreement
0.61–0.80 substantial agreement
0.81–1.00 almost perfect agreement

The principles of estimating the reproducibility of clinical recordings apply to the registration of dental caries, periodontal conditions, enamel fluorosis, dental erosion and oral mucosal conditions. Having images of conditions available may help in the clinical assessment. The level of agreement with regards to the recording of dental caries is relatively easy to calculate. However, due to the nature of periodontal disease, testing the reproducibility of periodontal scores is more complex. Thus the criteria for assessment should be thoroughly discussed as part of the calibration exercise.

1.4 Implementing the survey

1.4.1 General preparation

1.4.1.1 Contacts with persons in authority

The organization of a survey commences well before the intended start date of examinations. Persons in authority in the institutions or organizations where people will be examined must be contacted in advance. In schools, for example, the principal or head teacher should be consulted about school term dates and when the children would be available for examination, and whether there is a suitable area or room that could be used for the assessments. The principal may also be able to provide basic information about the socioeconomic level and nutritional status of the children, local water sources, seasonal accessibility of the school, and any health promotion or health education activities carried out in the school. If adults are included, there will be a need to coordinate their participation with their place of employment, or for older people, social services, nursing homes or other care institutions will need to be consulted.

1.4.1.2 Keeping a logbook

The organizer of the survey should maintain a logbook in which the location of each day's examinations, the number of persons examined, and information about each survey site is recorded. Occasionally, observations made and impressions formed at this time have an important bearing on later assessment of survey results. If these are not clearly described at the time of observation, they may be either forgotten or recalled inaccurately.

1.4.1.3 Preliminary exercise or pilot study

For investigators planning their first oral health survey, it is helpful to examine two classes of 12-year-old children in local schools, or a group of adults, as a preliminary exercise. This will give the survey personnel an opportunity to work together and to identify and discuss any organizational or technical

problems that may arise. Examiner calibration and training of recording clerks can be done at the same time. A more comprehensive activity simulating all aspects of the survey can be conducted in an urban or rural community. All members of the team who will be involved in the survey, including administrators, coordinators, examiners and recorders, should participate. Each member of the team should have the opportunity to exercise her or his assigned responsibility as a participant in the survey. All the contemplated activities should be developed according to the overall description of duties.

The principal officer in charge of the survey should ensure that all members of the team perform to the best of their ability. This will allow developing the survey in an efficient manner and obtaining information that is reliable and valid. A pilot study will save precious time, identify potential difficulties and prompt modifications that may be necessary before the actual survey is initiated.

1.4.1.4 Sources of fluoride

Fluoridated water is the main source of fluoride exposure, but fluoride may be present in food, drinks and other sources. Information should be gathered on sources of fluorides available to the survey population, including water, salt, milk and toothpastes, as well as on levels of usage of topical fluorides and other products likely to affect the development of dental caries.

In certain situations it is important to know whether population groups have been or are exposed to fluoride and such information can be gathered at the time of the oral health survey. A sample of drinking water may be collected at each examination site and sent to a laboratory for analysis of fluoride content. Clean polyethylene bottles or tubes of approximately 30–50 ml capacity should be used for this purpose. They should be rinsed in distilled water prior to rinsing twice with the water to be sampled. The bottles should then be filled, closed firmly and clearly labelled with a permanent marker, giving the date of collection, location and source of the water. In many countries, it is possible to obtain fluoride analysis services through public health water supply departments.

1.4.2 Personnel and organization

1.4.2.1 Recording clerk

Each examiner should be assisted by an alert and cooperative recording clerk who can follow instructions precisely and can neatly note down numbers and letters. The examiner should give the clerk clear instructions about recording the data on the assessment form. The meaning of the terms that will be used should be explained to the clerk and he or she should be instructed in

the coding systems so that, with practice, they will be able to recognize obvious mistakes or omissions made by the examiner. Before the survey begins, the clerk should practise by recording the findings of a few preliminary examinations. Special instructions must be given and additional practice undertaken if the clerk is not familiar with the alphabetical or numerical symbols used on the assessment form. Failure to ensure that the recording clerk is making clear entries may result in confusion between codes later on in the process.

When direct-entry computer systems are used, recording clerks should receive specific instructions and training in their use.

1.4.2.2 Organizing clerk

It is desirable to have an organizing clerk at each examination site to maintain a constant flow of subjects to the examiner(s) and to enter general descriptive information on the record forms. The organizing clerk should also check the finished records for accuracy and completeness, so that missing information may be obtained before the survey team moves to another location. This person should also be responsible for ensuring that the examiners have an adequate supply of sterile instruments.

1.4.2.3 Daily review of assessment forms

It is very important that every examiner reviews each day's assessment forms on the same day, for completeness and accuracy of recordings.

1.4.3 Instruments and supplies

The quantity and weight of instruments and supplies used in the survey should be kept to a minimum; however, sufficient numbers of instruments should be available to avoid the need to temporarily stop examinations while used ones are being sterilized. The following instruments and supplies are required for each examiner:

- instruments for oral examination: plane mouth mirrors; metallic periodontal probes (Community Periodontal Index (CPI) probe) that conform to WHO specifications, i.e. 0.5 mm ball tip; a black band between 3.5 and 5.5 mm and rings at 8.5 and 11.5 mm from the ball tip; and several pairs of tweezers;
- containers (one for used instruments and one for disinfecting or sterilizing instruments) and concentrated disinfecting solution in sufficient quantity;
- rubber gloves;
- wash basin for either water and soap or disinfectant solution;

- cloth or paper hand towels; and
- gauze.

Generally, a minimum of 30 mouth mirrors and 30 periodontal probes per examiner should be provided, as this will permit some instruments to be sterilized while the others are being used. Used instruments should be placed in disinfectant solution, then washed and drained well before sterilization.

1.4.4 Infection control

The survey coordinator and all personnel participating in the survey must be made aware of the possibility of cross-infection when conducting examinations or handling contaminated instruments (*15*). Current national recommendations and standards should be responsibly followed for both infection control and waste disposal.

Examiners are responsible for maintaining adequate infection control in survey procedures. During their training, it should be emphasized that all areas of the oral cavity can be fully examined with appropriate use of mouth mirrors and the periodontal probe, and without the need for digital manipulation of the oral tissues. This reduces the risk of cross-infection. Use of disposable masks and gloves and wearing of protective glasses are also recommended.

The survey team must have disinfecting solutions available in sufficient quantity. In the absence of an autoclave, domestic pressure cookers or pots can be used for sterilizing instruments (*16*). After putting a small amount of water in the cooker, the instruments are placed on a trivet so that they are above the surface of the water. Heat from any source is applied until steam starts to escape from the cooker's nozzle. Then the "regulator", which is a weight-"rocking device", is placed on the nozzle and the internal pressure allowed rising to reach approximately 1.0 bar (15 psi) following the pressure cooker manufacturer's instructions. The instruments are exposed to heat and pressure for 15–20 minutes. Then the cooker is removed from the heat source and the pressure is allowed to drop. The instruments are left to cool inside the pot.

1.4.5 Examination area

The area for conducting examinations should be planned and arranged for maximum efficiency and ease of operation. The exact arrangement will be determined by the physical condition of the site, but certain controllable features should be kept in mind. Examinations may be conducted in a dental office or in the field. The lack of a suitable building or a dental office does not preclude a survey from being performed. If necessary, examinations can be carried out outside (**Fig. 2**).

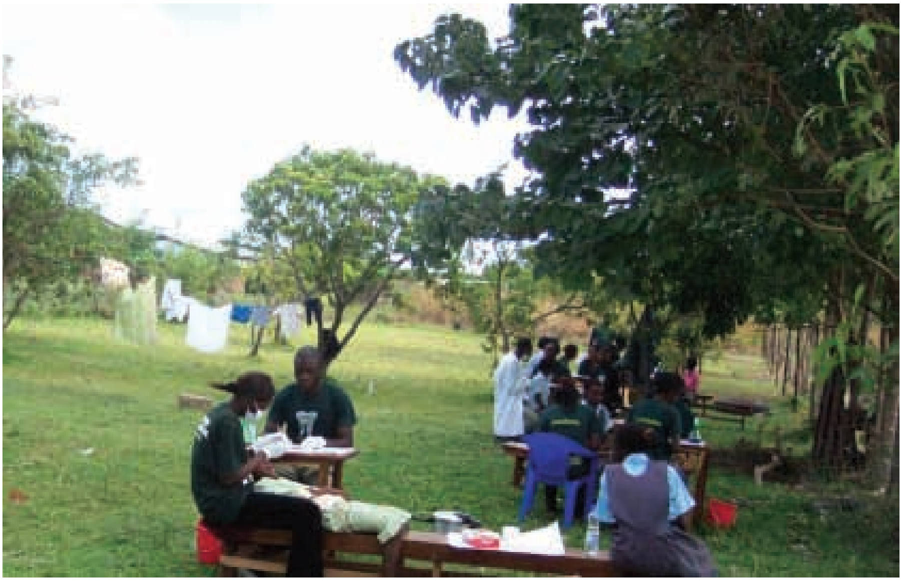

Fig. 2. Oral health examination being carried out in a school fi eld.

1.4.6 Examination position

The examination position of the subjects will depend on the furniture available. The most comfortable situation is for the subject to lie on a table or bench with the examiner seated behind the subject's head. Subjects can also be examined seated in a chair with a high backrest with the examiner standing behind or in front of the chair. If no furniture is available, subjects can be examined lying on a cloth on the ground with the examiner seated cross-legged behind the person's head.

1.4.7 Lighting

The lighting should be as consistent as possible throughout the survey. If electricity is available at all locations, a lightweight portable examination light (in the blue-white colour spectrum) should be used. Inflammatory and structural changes of the oral tissues are more difficult to detect under normal artificial light (yellow-red in colour) than under natural or corrected artificial light. If electricity or battery-operated lights are not available at some survey sites, natural light should be used at all locations.

If an artificial light source is used, the location of the electrical supply points will affect the positioning of the tables and chairs. The subject should

face away from any natural light source to avoid variation in illumination. However, if natural light alone is being used, the subject should be positioned so as to receive maximum illumination, while avoiding discomfort from direct sunlight to both the subject and the examiner. The chair or table should face the opening (e.g. window) through which the light is entering the room and be placed as close to it as possible.

1.4.8 Table or platform

A table or platform to hold the dental instruments and basins should be within easy reach of the examiner.

1.4.9 Seating of recording clerk

The recording clerk should sit close enough to the examiner for instructions and codes to be easily heard and for the examiner to check that findings are being recorded correctly. This arrangement also enables the recording clerk to keep a check that the score recorded relates to the region or tooth that has just been examined.

1.4.10 Supply of survey forms

An adequate supply of assessment forms, hardboard bases and clips, sharpened pencils, erasers and copies of the recording instructions, coding lists and measurement criteria should be readily available. Portable computers for direct digital entry of information may also be helpful. Care must be exercised to frequently back up data.

1.4.11 Avoidance of crowding

If possible, the examination area should be partitioned or arranged in such a way that subjects enter at one point and leave at another. Subjects should not be permitted to crowd around the examiner or recorder but should enter the examination area one at a time.

1.4.12 Avoidance of noise

High noise levels and loud conversations in the examining area can prevent the recording clerk from hearing scores called by the examiner and may distract the examiner and recorder, interrupting the "flow" of the examination.

1.5 Assessment of oral health status

1.5.1 Standard forms

Suitable standardized forms for recording clinical oral health assessments as described in this manual are provided in **Annex 1** (for adults) and **Annex 2** (for children). The assessment form for children only includes the variables relevant to young individuals. Survey planners needing to record the dentition status by tooth surface can use the special forms designed for this purpose (see **Annex 3** and **Annex 4**). Investigators should arrange to have copies reproduced locally.

A simplified assessment form for recording common oral manifestations of human immunodeficiency virus/acquired immunodeficiency syndrome (HIV/AIDS) is provided in **Annex 5**. The investigator is advised to consult the WHO publication *Guide for epidemiological studies of oral manifestations of HIV infection* (*17*), available at who.int/oral_health. The publication includes information on practical planning and implementation of surveys on oral lesions related to HIV/AIDS. The conditions considered essential to record are fungal infections, bacterial infections, viral infections and neoplasms.

Annex 6 illustrates the major oral conditions relevant to a standard oral health survey. The conditions shown in Plates 1–71 may be helpful in differential diagnosis of lesions, increasing accuracy of coding during the examination.

1.5.2 Standard codes

Standard codes must be used for all sections of the form(s). If this requirement is not observed, WHO will be unable to make recommendations for processing the data and summarizing the results. If some of the oral health assessments are not carried out, or are not applicable to the age group being examined, the unused sections of the form should be cancelled with a diagonal line, or by using code 9 in the appropriate box (= not recorded).

1234567890

A B C D E F G X

Fig. 3. Clear writing prevents confusion between numbers and alphabets that resemble each other (see text).

The forms are designed to facilitate computer processing of the observations. Each box is given an identification number (the small number in parentheses), which represents a location in a computer file. Recording codes are shown near the appropriate boxes. To minimize the number of errors, all entries must be clear and unambiguous. Confusing similarities in entries commonly occur while writing 1 and 7, 2 and 4, 6 and 0, and B and 8. To avoid confusion and erroneous entries that create problems for computing processing and consequently inaccurate results, numerals should be written out clearly (**Fig. 3**).

Again, clear enunciation is essential when calling out scores to recorders to differentiate unmistakably between similar sounding codes, e.g. eight and A. Examiners can dictate codes using common words in the local language, for example, in English, one can use Alpha, Beta, Coast, Day, Gamma, Fire and X-ray instead of A, B, C, D, G, F, X to facilitate clear understanding on the part of the recording clerk.

The two-digit numbers above or below some of the boxes indicate specific teeth according to the tooth notation system developed by WHO and FDI (previously called Fédération Dentaire Internationale, now World Dental Federation) (*18*). The first digit indicates the quadrant of the mouth the tooth is in and the second digit the actual tooth (**Fig. 4**). In designating a tooth, the examiner should call the quadrant number and then the tooth number, e.g. the upper right second incisor (12) is called out as "one-two" rather than "twelve"; the lower left third molar (38) should be called out as "three-eight" rather than "thirty-eight".

1.5.3 Oral health assessment form

The standard oral health assessment form for adults (**Annex 1**) includes the following sections:

- survey identification information
- general information
- extraoral conditions

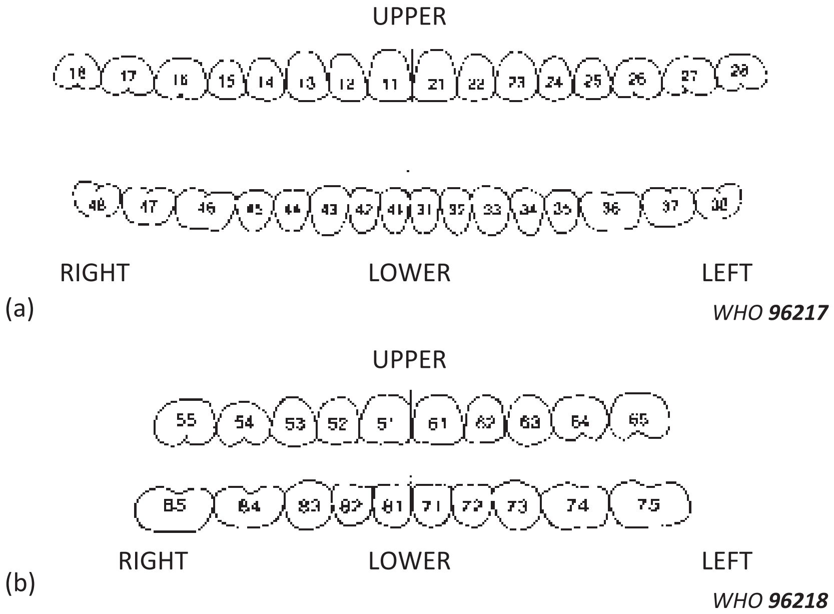

Fig. 4. WHO/FDI tooth notation used for coding of teeth: (a) permanent tooth notation and (b) primary tooth notation.

- dentition status (crown, root)
- periodontal status
- loss of attachment
- enamel fluorosis
- dental erosion
- dental trauma
- oral mucosal lesions
- denture status (fixed or removable dentures)
- intervention urgency and need for referral
- notes.

The corresponding standard oral health assessment form for children (**Annex 2**) includes the following sections:

survey identification information
general information
dentition status
gingival status
enamel fluorosis
dental erosion

dental trauma
oral mucosal lesions
intervention urgency.

With regard to oral mucosal lesions, if the sample consists only of children, a decision may be made to record only those lesions that are frequently observed in children rather than all the lesions that may occur in adults. It is strongly recommended that the appropriate form is used when surveying children or adults.

1.5.4 Identification and general information sections

During planning of the survey, a list of examination sites and a list of the examiners who will be involved in the study should be made, and a code assigned to each examiner. The coding list should also include the numeric codes to be used for other relevant information such as the fluoride content of drinking water or use of fluoride supplements. The investigator should write the name of the country in which the survey is being conducted in capital letters on the original assessment form before making additional copies. Boxes 1–4 on the form are reserved for the WHO code for the country in which the survey will be carried out and should not be filled in by the investigator.

Essential information includes year, month and day of examination (Boxes 5–10). The identification number is the unique identifier for the individual person under examination (Boxes 11–14); codes are also given to indicate whether the examination is the original or duplicate examination (Box 15) and by the individual examiner responsible for the examination (Boxes 16 and 17).

In the general information section, the following points are recorded: the name (write-in response if permissible to record), sex (Box 18), date of birth (Boxes 19–24), age (Boxes 25 and 26), ethnic group (Boxes 27 and 28), other group (Boxes 29 and 30), years attended school (Boxes 31 and 32), occupation (Box 33), geographical location/community (Boxes 34 and 35), type of location (Box 36), and other survey specific data (Boxes 37–42). Findings from the extra-oral examination are recorded in Boxes 43 and 44.

Date of examination (Boxes 5–10)

The year, month and day should be recorded on the form at the time of examination. Recording the day enables an investigator to refer back to examinations held on any particular day which may need to be reviewed or checked.

Identification number (Boxes 11–14)

Each subject examined should be given an identification number. This number should always have the same number of digits as the total number of subjects

to be examined. Thus, if it is intended to examine 1200 subjects, the first subject should be numbered 0001.

If possible, the identification numbers should be entered on the forms before commencing the examinations for the day, because it is important to ensure that each identification number is used only once. Cross-checking is necessary when more than one examiner is participating in a survey. If a total of 1200 subjects are to be surveyed by two examiners, examiner 1 should use the numbers 0001–0600 and examiner 2 should use 0601–1200.

Original or duplication examination (Box 15)

If the subject will be re-examined to assess reproducibility, the first (original) examination is scored “1” and any subsequent, duplication examinations are coded “2”, “3”, “4”, etc. in Box 15. For all subjects for whom duplicate examinations have been made, data from the first examination only are included in the survey analysis. To assess intra-examiner reproducibility, the first (original) examination is coded “1” and the second examination performed by the same examiner is coded “2”. When the findings of the survey are analysed, the level of intra-examiner reproducibility, denoted by the percentage of agreement and the kappa statistic, can be calculated and reported. It is important that the method selected is clearly explained to avoid misinterpretations in data entry.

Examiner (Boxes 16 and 17)

If more than one examiner is participating in the survey, each examiner should be assigned a specific code, which should be entered in Boxes 16 and 17. If a validating examiner is participating in the survey, he or she should also be assigned a specific code.

Name

The name of the subject may be written in block letters, beginning with the family name. It should be noted that, in some countries, identification of survey subjects by name is not permitted, in which case this space should be left blank.

Sex (Box 18)

This information should be recorded at the time of examination because it is not always possible to tell a person’s sex from the name alone (which may or may not be recorded). The relevant code (1 = male, 2 = female) is entered in Box 18.

Date of birth (Boxes 19–24)

Where possible, the year (Boxes 19 and 20), month (Boxes 21 and 22), and day of birth (Boxes 23 and 24) should be entered for cross-checking purposes.

Age (Boxes 25 and 26)

Age should be recorded as age at last birthday (e.g. a child in the thirteenth year of life is 12 years old). If the age is less than 10 years, enter "0" in Box 25 (e.g. 6 years is coded as "06"). In communities where age is expressed in different terms, a conversion must be made. If the age of the subject is not known, it may be necessary to make an estimate on the basis of, for instance, status of tooth eruption or, for adults, major life events or occasions in the community. Where age has been estimated, the manner of estimation should be reported.

Ethnic group (Boxes 27 and 28)

In different countries, ethnic and other groups are identified in different ways, by area or country of origin, race, colour, language, religion or tribal membership. Local health and education authorities should be consulted before making a decision about the relevant ethnic group classification/coding scheme to be used.

More than 10 ethnic groups may officially exist in a country; therefore a two-digit entry code should be used. Since it is often not possible to identify a person's ethnic origin from their name alone, ethnic group information must be recorded at the time of the examination from information provided by the subject/parent. In some countries, information on ethnicity may be obtained from government agencies or school administrative data at the time of sample selection.

Other group (Boxes 29 and 30)

Codes may be used to identify different subpopulation groups.

Number of years in school (Boxes 31 and 32)

This information is useful for assessment of the level of education, which is important factor in the analysis of oral health. In children, the boxes may be used for recording the school grade achieved by a child (**Annex 2**).

Occupation (Box 33)

A coding system should be devised according to local usage to identify different occupations and the appropriate code entered.

Community – geographical location (Boxes 34 and 35)

In these surveys, using codes to identify schools as part of the survey is important since the information obtained on oral health status can be used for planning strategies applicable to specific age groups in a particular school. Once the schools have been selected, the codes can be designated.

Boxes 34 and 35 should be used to record the site where the examination is being conducted. This allows geographical locations (villages etc.) to be identified (01–98). A list giving each location and its code number should be prepared. Usually just a few codes are needed. The code "99" should be entered if this information is not recorded.

Community information is very useful for health administrators for planning or revising programmes or strategies. If samples of drinking water are collected during the survey, the name of the community will be essential in mapping of fluoride in drinking water supplies and determining whether the concentration is below, at or above optimal levels.

Location type (Box 36)

The rationale for including these data is to obtain general information about local environmental conditions and the availability of services at each site. Three codes are used:

1. Urban site.
2. Periurban area: this has been included in order to indicate areas surrounding major towns that may have characteristics similar to those of rural areas, with very few health facilities of any kind and usually with no or limited access to oral health-care facilities.
3. Rural area or small village.

Other data (Boxes 37–42)

Fields are provided for entering a two-digit code for up to three conditions or data to be recorded. Conditions and codes must be agreed prior to commencing the survey. These fields have been provided for recording other information about the subjects examined or the survey location. Information such as exposure to a specific oral health programme, use of tobacco or chewing sticks, refugee status, socioeconomic status, physical environment, and level of fluoride in drinking water can be recorded here; if sugar intake was of interest, a coding system could be designed by the investigator whereby the amount and frequency of intake were given suitable codes.

Additionally, two boxes are reserved for recording any abnormality identified by the examiner during extra-oral examination of the oro-facial complex.

1.5.5 Clinical examination (Boxes 43 and 44)

The oral cavity is part of the orofacial complex and examiners should record any evident abnormality of the tissues of the face, nose, cheek or chin. The condition and its location are recorded using the following recommended codes:

Condition (Box 43)

0 = Normal
1 = Ulceration, sores
2 = Erosions
3 = Fissures
4 = Cancrum oris
5 = Enlarged lymph nodes
6 = Any other abnormalities
9 = Not recorded

Location (Box 44)

1 = Face
2 = Neck
3 = Nose
4 = Cheeks
5 = Chin
6 = Commissures
7 = Vermillion border
8 = Jaws

1.5.5.1 Dentition status

(Boxes 45–108 in the Oral Health Assessment Form for Adults and 45–72 in the Oral Health Assessment Form for Children)

The examination for dental caries should be conducted with a plane mouth mirror. The use of radiography for detection of approximal caries is not recommended because the equipment is impractical to utilize in most field situations. Likewise, the use of fibreoptics is not recommended. Although it is recognized that both these diagnostic aids reduce the underestimation of dental caries, logistical complications and frequent objections on the part of subjects to exposure to radiation outweigh any potential gains.

Examiners should adopt a systematic approach to the assessment of the dentition status, bearing the following points in mind:

- the examination should proceed in an orderly manner from one tooth or tooth space to the adjacent tooth or tooth space;

- a tooth should be considered present in the mouth when any part of it is visible
- if a permanent and primary tooth occupy the same tooth space, the status of the permanent tooth only should be recorded.

Permanent dentition status (crown and roots) is recorded using numbered scores and the primary dentition status is recorded using letter scores (**Table 2**) in the same boxes. Boxes 45–76 are used for upper teeth and Boxes 77–108 for lower teeth. The corresponding boxes for the Oral Health Assessment Form for Children are Boxes 45–58 and 59–72.

An entry must be made in every box pertaining to the coronal and root status of a tooth (**Annex 1**). In children, root status is not assessed; therefore the corresponding boxes have been omitted in the Oral Health Assessment Form for Children (**Annex 2**).

Considerable care should be taken to identify tooth-coloured fillings, which may be extremely difficult to detect.

Table 2. Coding the dentition status – primary and permanent teeth

Code			Condition/status
Primary teeth	Permanent teeth		
Crown	Crown	Root	
A	0	0	Sound
B	1	1	Caries
C	2	2	Filled, with caries
D	3	3	Filled, no caries
E	4	–	Missing due to caries
–	5	–	Missing for any other reason
F	6	–	Fissure sealant
G	7	7	Fixed dental prosthesis abutment, special crown or veneer/implant
–	8	8	Unerupted tooth (crown)/unexposed root
–	9	9	Not recorded

The criteria for diagnosing a tooth status and the coding are as follows (codes applied to primary teeth are given in parentheses):

0 (A) *Sound crown.* A crown is coded as sound if it shows no evidence of treated or untreated clinical caries (see Plate 1, code A, and Plate 6, code 0). The stages of caries that precede cavitation, as well as other conditions similar to the early stages of caries, are excluded because they cannot be reliably identified in most field conditions in which epidemiological surveys are conducted. Thus, a crown with the following defects, in the absence of other positive criteria, should be coded as sound (see Plates 7 and 8):

- white or chalky spots; discoloured or rough spots that are not soft to touch with a metal CPI probe;
- stained enamel pits or fissures that do not have visible cavitation or softening of the floor or walls detectable with a CPI probe;
- dark, shiny, hard, pitted areas of enamel in a tooth showing signs of moderate to severe enamel fluorosis;
- lesions that, on the basis of their distribution or history, or on examination, appear to be due to abrasion.

Sound root. A root is recorded as sound when it is exposed and shows no evidence of treated or untreated clinical caries (see Plate 9).

1 (B) *Carious crown.* Caries is recorded as present when a lesion in a pit or fissure, or on a smooth tooth surface, has an unmistakable cavity, undermined enamel, or a detectably softened floor or wall (see Plates 2–4 Primary dentition, and Plates 10–12 Permanent dentition). A tooth with a temporary filling, or one which is sealed but also decayed, should also be included in this category. In cases where the crown has been destroyed by caries and only the root is left, the caries is judged to have originated in the crown and is therefore scored as crown caries only. The CPI probe should be used to confirm visual evidence of caries on the tooth surface(s). Where any doubt exists, caries should not be recorded as present.

Carious root. Caries is recorded as present when a lesion feels soft or leathery on probing with the CPI probe. If the carious lesion on the root does not involve the crown, it should be recorded as root caries. For single carious lesions affecting both the crown and the root, the likely site of origin of the lesion should be recorded as the decayed site. When it is not possible to identify the site of origin, both the crown and the root should be coded as decayed. In general, root caries is not recorded for children and in youth or young adults.

2 (C) *Filled crown, with caries.* A crown is considered filled, with decay, when it has one or more permanent restorations and one or more areas that are decayed. No distinction is made between primary and secondary caries and the same code applies regardless of whether the carious lesions are in contact with the restoration(s) (see Plates 13 and 14).

Filled root, with caries. A root is considered filled, with caries, when it has one or more permanent restorations and one or more areas that are decayed. No distinction is made between primary and secondary caries. In the case of restorations involving both the crown and the root, identification of the site of origin is more difficult. For any restoration involving both the crown and the root with secondary caries, the most likely site of the primary carious lesion is recorded as filled, with decay. When it is not possible to identify the site of origin of the primary carious lesion, both the crown and the root should be coded as filled, with caries.

3 (D) *Filled crown, with no caries.* A crown is considered filled, without caries, when one or more permanent restorations are present and there is no caries anywhere on the crown (see Plates 5 and 15). A tooth that has been crowned because of previous decay is recorded in this category. A tooth that has been crowned for reasons other than caries by means of a fixed dental prosthesis abutment is coded 7 (G).

Filled root, with no caries. A root is considered filled, without caries, when one or more permanent restorations are present and there is no caries anywhere on the root. In the case of fillings involving both the crown and the root, identification of the site of origin is more difficult. For any restoration involving both the crown and the root, the most likely site of the primary carious lesion is recorded as filled. When it is not possible to identify the site of origin, both the crown and the root should be coded as filled.

4 (E) *Missing tooth, due to caries.* This code is used for permanent or primary teeth that have been extracted because of caries and are recorded under coronal status (see Plates 16 and 17). For missing primary teeth, this score should be used only if the subject is at an age when normal exfoliation would not be a sufficient explanation for absence.

Note: *The root status of a tooth that has been scored as missing because of caries should be coded "7" or "9".*

In some age groups, it may be difficult to distinguish between unerupted teeth (code 8) and missing teeth (codes 4 or 5). Basic knowledge of tooth eruption patterns, the appearance of the alveolar ridge in the area of the tooth

space in question, and the caries status of other teeth in the mouth may provide helpful clues in making in deciding whether a tooth is unerupted or has been extracted. Code 4 should not be used for teeth deemed to be missing for any reason other than caries. For convenience, in fully edentulous arches, a single "4" should be placed in Boxes 45 and 60; and 77–92, as appropriate, and the respective pairs of numbers linked with straight lines. Such procedure may also be applied where the record form for registration at tooth surface level is used (**Annex 3 and Annex 4**).

5 (–) *Permanent tooth missing due to any other reason.* This code is used for permanent teeth deemed to be absent congenitally, or extracted for orthodontic reasons or because of periodontal disease, trauma, etc. (see Plate 18). As for code 4, two entries of code 5 can be linked by a line in cases of fully edentulous arches.

Note: *The root status of a tooth scored 5 should be coded "7" or "9".*

6 (F) *Fissure sealant.* This code is used for teeth in which a fissure sealant has been placed on the occlusal surface, in pits or for teeth in which the occlusal fissure has been enlarged with a rounded or "flame-shaped" bur, and a composite material placed (see Plate 19). If a tooth with a sealant has caries, it should be coded as 1 or B.

7 (G) *Fixed dental prosthesis abutment, special crown or veneer.* This code is used under coronal status to indicate that a tooth forms part of a fixed bridge abutment. This code can also be used for crowns placed for reasons other than caries and for veneers or laminates covering the labial surface of a tooth, on which there is no evidence of caries or a restoration (see Plate 20).

Note: *Missing teeth replaced by fixed partial denture pontics are coded 4 or 5 under coronal status, while root status is scored 9.*

Implant. This code is used under root status to indicate that an implant has been placed as an abutment.

8 (–) *Unerupted tooth (crown).* This classification is restricted to permanent teeth and used only for a tooth space with an unerupted permanent tooth but no primary tooth. Teeth scored as unerupted are excluded from all calculations concerning dental caries (see Plate 1 (incisors) and Plate 21). This category does not include congenitally missing teeth, or teeth lost as a result of trauma etc. For differential diagnosis between missing and unerupted teeth, see code 5.

Unexposed root. This code indicates that the root surface is not exposed; there is no gingival recession beyond the cement-enamel junction (CEJ).

9 (–) *Not recorded.* This code is used for an erupted permanent tooth that cannot be examined for any reason such as orthodontic bands, severe hypoplasia, etc.

This code is used under root status to indicate either that the tooth has been extracted or that calculus is present to such an extent that root examination is not possible.

Dental caries indices: tooth (DMFT, dmft) and surface levels (DMFS, dmfs)

Information on the Decayed, Missing and Filled Teeth Index (DMFT) can be derived directly from the data in Boxes 45–76 and 77–108 (**Annex 1**). The D component includes all teeth with codes 1 or 2. The M component comprises teeth coded 4 in subjects under 30 years of age, and teeth coded 4 or 5 in subjects 30 years and older, i.e. missing due to caries or for any other reason. The F component includes teeth only with code 3. The basis for DMFT calculations is 32 teeth, i.e. all permanent teeth including wisdom teeth. Teeth coded 6 (fissure sealant) or 7 (fixed dental prosthesis/bridge abutment, special crown or veneer/implant) are not included in calculations of the DMFT index. In the case of the primary teeth, the calculation of the dmft index is similar, i.e. by deriving information from data codes A, B, C and D and E in the oral health assessment form (**Annex 2**).

When a survey is undertaken for a particular purpose, e.g. evaluation of a disease prevention programme, planners may wish to record dentition status by tooth surface and to calculate the DMFS and dmfs indices. Record forms for this purpose are available in **Annex 3** (for adults) and **Annex 4** (for children).

A DFT index applicable to roots can easily be calculated as data for each tooth are collected during examination; this index is especially relevant in older population groups.

1.5.5.2 Periodontal status: Community Periodontal Index (CPI) modified

Two indicators of periodontal status are used for this assessment: gingival bleeding and periodontal pockets. A specially designed, lightweight CPI metallic probe with a 0.5-mm ball tip is used, with a black band between 3.5 and 5.5 mm, and rings at 8.5 and 11.5 mm from the ball tip (**Fig. 5**) (*19*). All teeth present in the mouth are examined for absence or presence of gingival bleeding and absence or presence of periodontal pockets; pocket depth is measured with the WHO CPI periodontal probe.

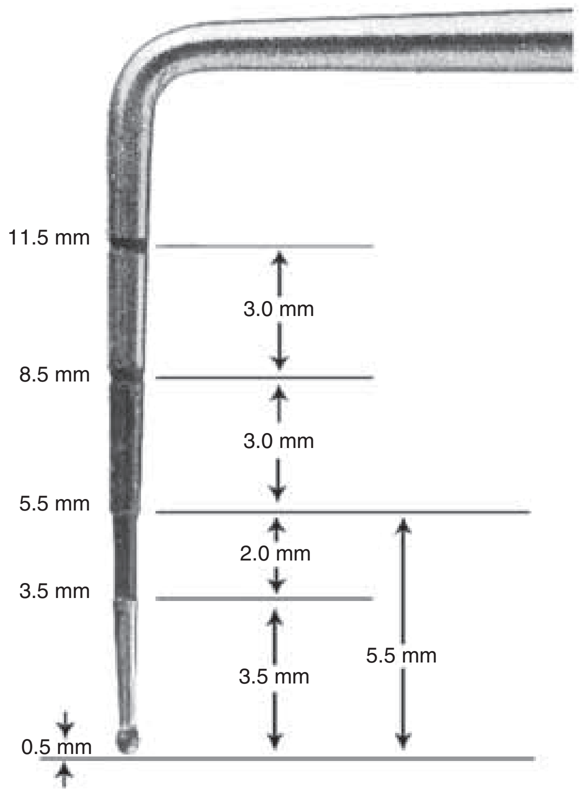

Fig. 5. The WHO Community Periodontal Index probe recommended for clinical examination.

Assessing for gingival bleeding and measuring periodontal pockets

Gingivae of all teeth present in the mouth should be examined by carefully inserting the tip of the WHO CPI probe between the gingiva and the tooth to assess absence or presence of bleeding response (**Fig. 6**). The sensing force used should be no more than 20 g. A practical test for establishing this force is to ask examiners to place the probe point under their thumbnail and press until blanching occurs. Alternatively, examiners can use a mirror and insert the probe into the gingival sulcus of their own anterior teeth using the lightest possible force that will allow movement of the probe ball tip along the tooth surface. These exercises should be conducted as part of the training when examiners are calibrated for reliability and consistency.

When the probe is inserted, the ball tip should follow the anatomical configuration of the surface of the tooth root. If the subject being examined feels pain during probing, this is indicative of the use of too much force. The probe

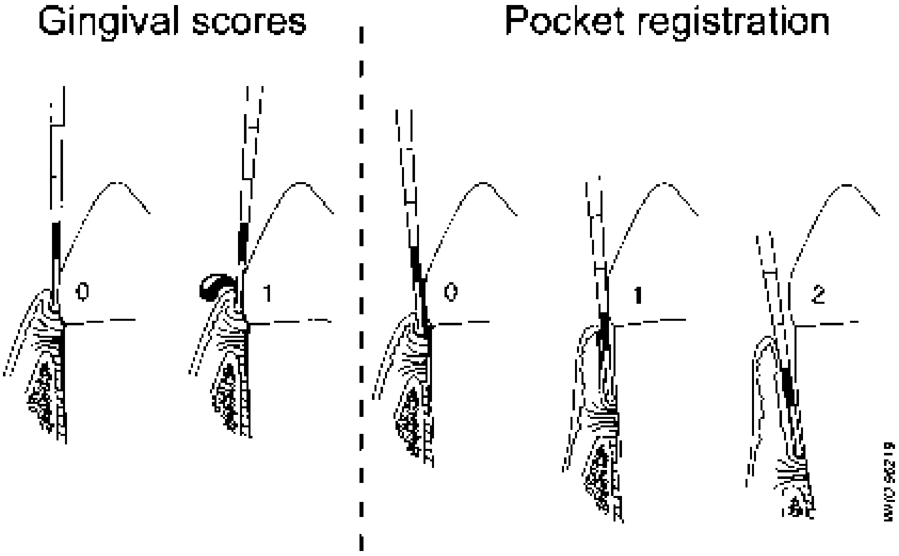

Fig. 6. Coding of periodontal status consistent to the modifi ed Community Periodontal Index (CPI modifi ed), showing the correct positioning of the WHO CPI probe.

tip should be inserted gently into the gingival sulcus or pocket and the full extent of the sulcus or pocket explored. For example, place the probe in the pocket at the distobuccal surface of the second molar, as close as possible to the contact point with the third molar, keeping the probe parallel to the long axis of the tooth. Move the probe gently, with short upward and downward movements, along the buccal sulcus or pocket, to the mesial surface of the second molar. A similar procedure is carried out for lingual surfaces, starting on the distolingual aspect of the second molar.

All teeth present should be probed and scored in the corresponding box. Periodontal pockets are not recorded in individuals younger than 15 years of age. The codes for scoring bleeding and pocketing are given below.

Gingival bleeding scores

(Boxes 109–124 and 141–156 in the Oral Health Assessment Form for Adults; Boxes 73–86 and 87–100 in the Oral Health Assessment Form for Children)

0 = Absence of condition (see Plate 22, adult).
1 = Presence of condition (see Plate 23 child; Plate 24 adolescent; Plate 25 adult).
9 = Tooth excluded (see Plate 28, Tooth 16).
X = Tooth not present (see Plates 16 and 17, posterior areas in adults).

Pocket scores

(Boxes 125–140 and 157–172)

0 = Absence of condition (see Plate 22).
1 = Pocket 4–5 mm (see Plate 26).
2 = Pocket 6 mm or more (see Plate 27).
9 = Tooth excluded (see Plate 28, Tooth 16).
X = Tooth not present (see Plates 16 and 17, posterior regions).

1.5.5.3 Loss of attachment (Boxes 173–178 Oral Health Assessment Form for Adults)

Information on loss of attachment may be collected from the index teeth (**Fig.** 7). The CPI system is designed to obtain an estimate of the lifetime accumulated destruction of the periodontal attachment and thereby permits comparisons between population groups. It is not designed to describe the full extent of loss of attachment in an individual. Loss of attachment is recorded by dividing the mouth in sextants, defined by tooth numbers: 18–14, 13–23, 24–28, 38–34, 33–43, and 44–48. The most reliable method of examination for loss of attachment in each sextant is to record this immediately after recording the gingival status and pocket scores. As mentioned above, loss of attachment should not be recorded for individuals under the age of 15.

Index teeth.

The index teeth, which are the teeth to be examined, are shown in **Figure** 7.

The two molars in each posterior sextant are paired for recording and, if one is missing, there is no replacement. If no index tooth is present in a sextant qualifying for examination, all the teeth that are present in that sextant are examined and the highest score is recorded as the score for the sextant.

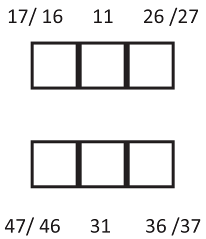

Fig. 7. The index teeth for recording loss of attachment in subjects aged 15 and over.

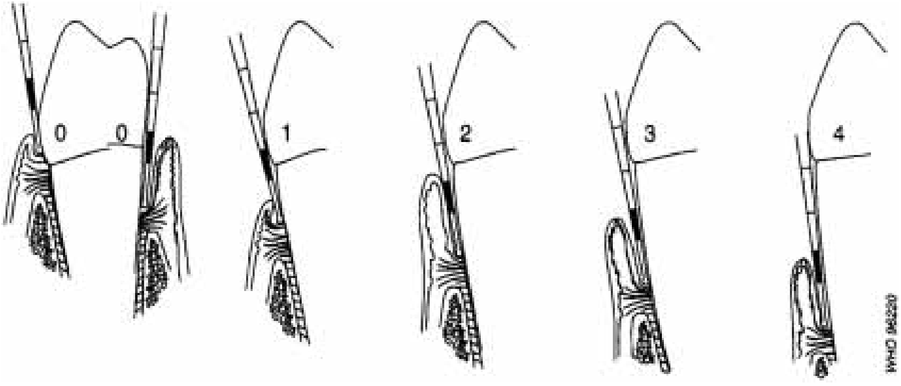

Fig. 8. Examples of coding for loss of attachment with a WHO CPI probe (*4*).

The extent of loss of attachment is recorded using the CPI probe and applying the following codes (**Fig. 8**):

0 = 0–3 mm
1 = 4–5 mm (CEJ within black band) (see Plate 26)
2 = 6–8 mm (CEJ between upper limit of black band and 8.5 mm ring)
3 = 9–11 mm (CEJ between 8.5 mm and 11.5 mm ring)
4 = 12 mm or more (CEJ beyond 11.5 mm ring) (see Plate 28, tooth 16)
X = Excluded sextant
9 = Not recorded

1.5.5.4 Enamel fluorosis

(Box 179 in the Oral Health Assessment Form for Adults; Box 101 in the Oral Health Assessment Form for Children)

Fluorotic lesions are usually bilaterally symmetrical and tend to show a horizontal striated pattern across the tooth. The premolars and second molars are most frequently affected, followed by the maxillary incisors. The mandibular incisors are least affected.

The examiner should note the distribution pattern of any defects, using Dean's index criteria (*20*), and make a decision as to whether they are typical of fluorosis. Defects falling into the "questionable" to "mild" categories – the conditions most likely to be encountered – may consist of fine white lines or patches and tend to fade into the surrounding enamel. To facilitate differentiating fluorosis lesions from other opacities not related to fluoride, it is important to remember that fluorosis lesions are usually observed near the edges of incisors or cusp tips; however, depending on severity, the lesions may

be readily apparent on other areas of the tooth and be readily visible in premolars and molars. Non-fluoride related opacities can be localized to the centre of the smooth surface, although they can affect the entire crown. Fluorosis lesions generally appear as fine lines, frosted in appearance and non-fluoride opacities appear round or oval in shape. Fluorosis lesions also can be more easily observed with the light directed in a tangential direction whereas non-fluoride opacities can be easily observed with the light directed perpendicularly to the tooth surface.

Coding is done on the basis of the two most severely affected teeth. If the two teeth are not equally severely affected, the score is based on the appearance of the less affected tooth. When the teeth are scored, the examiner should start at the higher end of the index, "severe", and eliminate each score until he or she arrives at the condition present. If there is any doubt, the lower score should be given.

The codes and criteria are as follows:

0 = *Normal.* Enamel surface is smooth, glossy and usually a pale creamy-white colour (see Plate 29)
1 = *Questionable.* The enamel shows slight aberrations in the translucent normal enamel and which may range from a few white flecks to occasional spots (see Plates 30–33)
2 = *Very mild.* Small, opaque, paper-white areas scattered irregularly over the tooth but involving less than 25% of the labial tooth surface (see Plates 34 and 35)
3 = *Mild.* White opacities of the enamel involving more than 25% (see Code 2) but less than 50% of the tooth surface (see Plates 36 and 37)
4 = *Moderate.* The enamel surfaces show marked wear, and brown staining is frequently a disfiguring feature (see Plates 38 and 39)
5 = *Severe.* The enamel surfaces are severely affected and the hypoplasia is so marked that the general form of the tooth may be affected. There are pitted or worn areas and brown stains are widespread; the teeth often have a corroded appearance (see Plates 40 and 41)
8 = *Excluded* (e.g. a crowned tooth)
9 = *Not recorded*

1.5.5.5 Dental erosion

(Boxes 180–182 in the Oral Health Assessment Form for Adults; Boxes 102–104 in the Oral Health Assessment Form for Children)

Data on prevalence, severity and number of teeth affected by dental erosion would assist public health administrators in estimating whether this condition is a public health problem. Dental erosion results from the progressive loss of calcified dental tissue by chemical processes not associated with

bacterial action. Enamel tissue is lost by exposure to acids which may come from dietary sources or may be intrinsic, i.e. in individuals suffering from bulimia, gastro-oesophageal reflux or heavy alcohol consumption and chronic vomit.

The following codes 1–3 are used where the crown of a tooth shows an erosion lesion at different levels:

0 = No sign of erosion (see Plate 42)
1 = Enamel lesion (see Plate 43)
2 = Dentinal lesion (see Plate 43, 44 and 46)
3 = Pulp involvement (see Plates 44–46)

Severity of dental erosion is recorded according to the tooth with the highest score of erosion. In addition, the number of teeth involved is recorded.

1.5.5.6 Traumatic dental injuries

(Boxes 183–185 in the Oral Health Assessment Form for Adults; Boxes 105–107 in the Oral Health Assessment form for Children)

Teeth affected by dental trauma are coded as follows:

0 = No sign of injury
1 = Treated injury
2 = Enamel fracture only (Plate 47)
3 = Enamel and dentine fracture (Plate 48)
4 = Pulp involvement (Plate 49)
5 = Missing tooth due to trauma (Plate 50)
6 = Other damage
9 = Excluded tooth

In addition to the degree/status of trauma, the severity of dental trauma can be measured in terms of the number of teeth involved.

1.5.5.7 Oral mucosal lesions

(Boxes 186–191 in the Oral Health Assessment Form for Adults; Boxes 108–113 in the Oral Health Assessment Form for Children)

The oral mucosa and soft tissues in and around the mouth should be examined in every subject. The examination should be thorough and systematic, and performed in the following sequence:

1. labial mucosa and labial sulci (upper and lower)
2. labial part of the commissures and buccal mucosa (right and left)
3. tongue (dorsal and ventral surfaces, margins)

4. floor of the mouth
5. hard and soft palate
6. alveolar ridges/gingiva (upper and lower).

Either two plane mouth mirrors or one mirror and the handle of the periodontal probe can be used to retract the tissues. Boxes 186–188 (108–110 in children) should be used to record the absence, presence, or *suspected* presence of the conditions coded 1–7. Examiners should be alert to, and can make a tentative diagnosis for, these conditions during clinical examination. Code 8 should be used to record a condition not mentioned in the list; for example, hairy leukoplakia or Kaposi sarcoma. Whenever possible, the tentative diagnosis should be specified in the space provided, for up to three conditions.

The following codes apply for adults:

0 = No abnormal condition
1 = Malignant tumour (oral cancer) (see Plate 51)
2 = Leukoplakia (see Plate 52)
3 = Lichen planus (see Plate 53)
4 = Ulceration (aphthous, herpetic, traumatic) (see Plates 54–57)
5 = Acute necrotizing ulcerative gingivitis (ANUG; see Plate 58)
6 = Candidiasis (see Plates 59 and 60)
7 = Abscess (see Plates 61 and 62).
8 = Other condition (specify if possible) (e.g. keratosis, see Plate 63; and Koplick spots, see Plate 64)
9 = Not recorded

Recording of leukoplakia and lichen planus is not considered important in children.

In addition, all the main locations of the oral mucosal lesion should be recorded in Boxes 189–191 for adults and Boxes 111–113 for children, as follows:

0 = Vermillion border
1 = Commissures
2 = Lips
3 = Sulci
4 = Buccal mucosa
5 = Floor of the mouth
6 = Tongue
7 = Hard and/or soft palate
8 = Alveolar ridges/gingiva
9 = Not recorded

For example, if an adult subject has leukoplakia on both the buccal mucosa and the commissures, the coding would be done as shown in **Figure 9**.

(186)	2	4	(189)
(187)	2	1	(190)
(188)			(191)

Fig. 9. Coding for leukoplakia in two main locations.

(186)	1	1	(189)
(187)	1	2	(190)
(188)	6	6	(191)

Fig. 10. Coding for more than two oral lesions.

Similarly, where an adult subject has oral cancer on the commissures and the lower lip, and candidiasis on the tongue, the coding should be done as shown in **Figure 10**.

1.5.5.8 Denture status

(Boxes 192 and 193 in the Oral Health Assessment Form for Adults)

The presence of removable dentures should be recorded for each jaw (Box 192, upper jaw; Box 193, lower jaw). The codes are as follows:

0 = No denture
1 = Partial denture
2 = Complete denture
9 = Not recorded

1.5.5.9 Intervention urgency

(Box 194 in the Oral Health Assessment Form for Adults; Box 114 in the Oral Health Assessment Form for Children)

It is the responsibility of the examiner or team leader to ensure that referral to an appropriate health-care facility is made, if needed. There is a need for immediate care if pain, infection or serious illness is present or is likely to occur unless treatment is provided within a certain period of time. This period may vary from a few days to a month, depending on the availability of oral

health services. Examples of conditions that require immediate attention are periapical abscess and ANUG. Advanced dental caries and chronic alveolar abscesses may also be recorded in this box. A life-threatening condition (oral cancer or precancerous lesions) or any other severe condition that is a clear oral manifestation of a systemic disease should have been recorded in the oral mucosa section and a code should also be entered in Box 194 in the form for adults or in Box 114 in the form for children.

The following intervention urgency codes are recommended:

0 = No treatment needed (see Plate 65)
1 = Preventive or routine treatment needed (see Plate 66)
2 = Prompt treatment including scaling needed (see Plate 67)
3 = Immediate (urgent) treatment needed due to pain or infection of dental and/or oral origin (see Plates 68–70)
4 = Referred for comprehensive evaluation or medical/dental treatment (systemic condition) (see Plate 71)

Section 2: Oral health self-assessment

2.1 Self-assessment of oral health and risks

2.1.1 Oral health information system

WHO recommends that countries should establish a complete oral health information system for monitoring and continued evaluation of national oral health programmes (*8*). The essential components of an oral health information system are depicted in **Figure 11**. Comprehensive information about oral health-care coverage, delivery of care, quality of care, and intermediate and ultimate outcomes of oral health intervention is important for ensuring an effective national oral health system. Population data on oral health status and prevalence of risk factors are relevant for surveillance of disease patterns and understanding trends over time; in addition, such information is instrumental for planning or adjustment of interventions by health authorities. The information recorded in the clinical record forms discussed in Section 1 of this manual allow for outcome evaluation of programmes; in the General Information section of the form, multiple coding is possible for recording of specific intervention(s) in relation to certain target groups and the facility has been provided for coding health administration procedures.

Systematic information on the prevalence of risk factors is important for planning community-oriented oral disease prevention and oral health promotion programmes. By merging clinical oral health data and risks factors data into a common database, the oral health effects of socio-behavioural factors could be estimated and relevant intervention strategies designed. Consequently, oral health programmes may be planned more effectively so they meet the needs of specific population groups. In addition to the above, information on self-assessed oral health is essential for identification of appropriate approaches in oral health promotion.

2.1.2 Self-assessment of oral health through use of questionnaires

According to the STEPS approach (**Fig. 1**), Step 1 represents collection of health data by means of questionnaires. When appropriately planned,

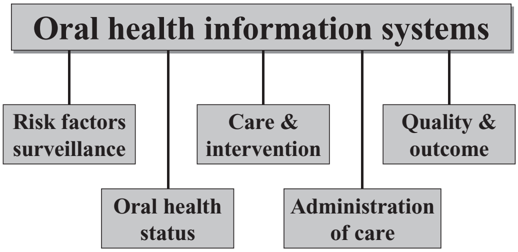

Fig. 11. Components of a comprehensive oral health information system (*5*).

questionnaires can be very useful in obtaining reliable information about health status and risks to health (*21, 22*). Importantly, data collection is less time-consuming and less costly for the country. Participants filling out a questionnaire survey must be appropriately informed about the objectives of the study; each participant must be ensured anonymity and informed that the data will be used for statistical purposes only. Thus, informed consent must be obtained. Participants are also entitled to know about any publication of preliminary results. Where questionnaires involve schoolchildren, the school principal or head teacher and the staff involved with the survey must be properly informed about the results.

Health questionnaires must be simple and include robust questions. A practical health questionnaire should need only 15–20 minutes to complete. Structured questionnaires imply that:

1. all questions have been formulated and the range of answers have been prepared in advance;
2. the sequence of responding to the questions is fixed;
3. filter questions need to be answered by some respondents only;
4. questions are formulated in a standardized manner: they are phrased using easily understood language; and
5. the list of response alternatives should be comprehensive and sufficient.

The questionnaire must be pretested to assess face validity and acceptability of questions. This procedure can help avoid potential misunderstanding of a poorly worded question by respondents and asking about two or more items in one question.

Self-assessment questionnaires can be prepared either for completion via an interview or for self-completion. The choice of method of data collection will depend on several factors such as the ability of participants to answer questions, practical circumstances, availability of staff, resources available for data collection, and the timetable for the survey. Data collection through

interviews may ensure that answers to the questions are given by the participants selected. Compared with questionnaires for self-completion, interviewing yields somewhat higher response rates. There is a higher degree of flexibility in responses whereas written questionnaire replies tend to be stringent. Interviewers are to pose questions exactly as written, and in the same sequence as they appear in the questionnaire; the interview, however, allows for further explanation of the meaning of a question.

As with clinical recordings, there are potential sources of variation in answers obtained through interviews. Variation can occur due to intra-interviewer variability or inter-interviewer variability. Intra-interviewer variability may arise if an interviewer is not consistent in the way he or she asks questions and/or records answers. When there is more than one interviewer, there may be inter-interviewer variability, whereby the various interviewers are not consistent in the ways they ask questions and/or record answers. The way an interviewer presents the questions may affect the answers given by respondents; thus potential reliability issues should be addressed during training.

In questionnaires for self-completion, the respondent reads all questions and the fixed response options her or himself. This type of questionnaire tends to be shorter to reduce the risk of respondent fatigue. A question can sometimes be constructed based on information specified by the categories of answers. However, compared with interviews, questionnaires for self-completion are practical, e.g. they can be posted to the respondent. Moreover, the external effect of an interviewer reading out the questions and answers is avoided and the respondent may have more time for giving correct answers to questions.

2.1.2.1 Training of interviewers and supervision

Interviewers need appropriate training to achieve consistency and a systematic approach to implementation of a health questionnaire survey. Interviewers must be familiar with the objectives of the questionnaire survey and the rationale behind the questions included.

Training in use of structured questionnaires should focus on the following areas:

- contacting prospective respondents and providing an introduction to the survey that explains its objectives and confirms that participation in the survey is anonymous;
- reading out questions as written and following the instructions in the interview schedule, for example in connection with filter questions;
- appropriate styles of inquiry and probing;
- recording exactly what is said;
- maintaining an interview style that does not bias the answers of respondents;

- maintaining an attitude of neutrality in order to avoid influencing the replies of the respondent; and
- field training and pretesting of the questionnaire.

Supervision of interviewers can be achieved in several ways:

- checking individual interviewer's response rates;
- audio-recording at least a sample of interviews;
- examining completed schedules to determine whether any questions are being omitted or if they are being completed properly; and
- call back a sample of respondents (usually around 10%) to confirm they were interviewed and to ask their opinion about the interviewer's conduct.

2.1.3 Oral health risk indicators within the frame of STEPS

WHO has proposed an operational model (**Fig. 12**) for use by investigators or public health administrators when considering an appropriate intervention (*5*). This model aims to guide the gathering of data by focusing on socio-environmental determinants and modifiable risk factors of oral health such as diet/nutrition, tobacco use and excessive alcohol consumption. In addition, information is required about environmental exposure to fluoride, oral hygiene practices and use of available oral health services. Quality of life, oral health and systemic health are considered important outcomes of the specified distal and proximal factors. The model thereby provides a conceptual and practical basis for linking oral health to relevant chronic disease assessment.

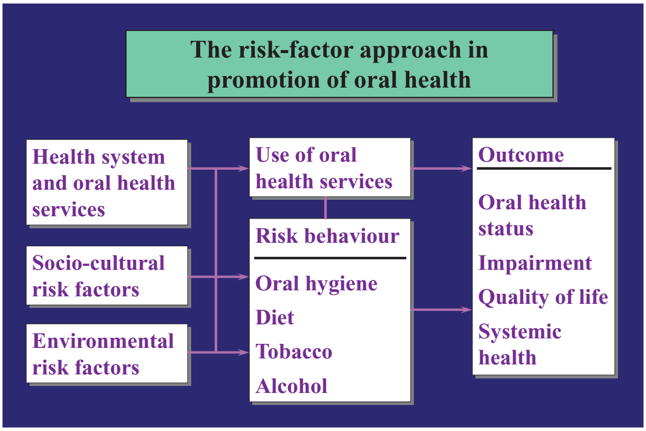

Fig. 12. Oral health risk factors relevant to planning and surveillance of oral health intervention programmes (*5*).

WHO recommends the use of simplified structured questionnaires for collection of self-assessed data on oral health and risk factors in adults (**Annex 7**) and in children or adolescents (**Annex 8**). Both questionnaires have been pilot-tested in a range of countries across the world. The simplified questionnaires include the core questions considered essential in national oral health surveillance, however, the questions and answers may be adapted to local or national settings. A country may wish to include additional questions and WHO can assist planners of a survey in their preparation of an expanded questionnaire to meet specific needs.

The variables included in the adult questionnaire are as follows:

Question 1 – general information (ID number, sex, location)
Question 2 – age
Question 3 – self-reported number of teeth present
Question 4 – experience of pain/discomfort from teeth and mouth
Question 5 – wearing of removable dentures
Question 6 – self-assessment of status of teeth and gums
Question 7 – frequency of tooth cleaning
Question 8 – use of aids for oral hygiene
Question 9 – use of toothpaste containing fluoride
Question 10 – dental visits
Question 11 – reason for dental visit
Question 4 and Question 12 – experience of reduced quality of life due to oral problems
Question 13 – consumption of sugary foods and drinks
Question 14 – use of tobacco: type and frequency
Question 15 – consumption of alcohol
Question 16 – level of education

The variables included in the child/adolescent questionnaire are as follows:

Question 1 – general information (ID, sex, location)
Question 2 – age
Question 3 – self-assessment of status of teeth and gums
Question 4 – experience of pain/discomfort related to teeth
Question 5 – dental visits
Question 6 – reason for dental visit
Question 7 – frequency of tooth cleaning
Question 8 – use of aids for oral hygiene
Question 9 – use of toothpaste containing fluoride
Question 4 and Question 10 – experience of reduced quality of life due to oral problems
Question 11 – consumption of sugary foods and drinks

Question 12 – use of tobacco: type and frequency
Question 13 and Question 14 – level of education of parents

A questionnaire survey of risks to oral health (Step 1) can be carried out as a stand-alone activity or in combination with a clinical oral health survey (Step 2). In a combined survey, the data may provide an opportunity for assessing the health impact of risk factors; this is possible only when identical ID codes are allocated in the both surveys and the questionnaire is administered just before the clinical data are collected.

Depending on the background of the respondents, the questionnaires provided in **Annex 7** and **Annex 8** can be filled either by an interviewer or they can be self-completed. In general, children aged 12 years and adolescents are considered capable of self-completion, whereas parents need to be involved in collection of data from younger children. As the oral health questionnaires include standardized questions, where a survey is to be carried out by interview, this may be undertaken by non-dental personnel. Interviewing requires appropriate field training of survey staff and a pilot study of 15–20 subjects should be carried out prior to the actual survey for ensuring face validity and reliability.

The WHO chronic disease surveillance programme (STEPS) incorporates a module on oral health of adults (*9*). However, the adult oral health questionnaire included in the standard version of STEPS does not focus on consumption of sugars, use of tobacco and alcohol, and education, as these items are already part of the general module. In addition, the main WHO STEPS questionnaire includes specific questions on general health and disease conditions which are relevant to oral health, e.g. diabetes, HIV/AIDS, nutrition status, and body mass index (BMI). WHO STEPS contains robust questions for measurement of diabetes from medical history or information about diagnosis, and Body Mass Index (BMI) defined as weight (kg) divided by the square of height (m) and the waist-hip ratio (WHR) defined as waist circumference (cm) divided by hip circumference (cm).

For planning and evaluation of school-based oral health programmes, a special oral health questionnaire is available from WHO. This questionnaire may be used for collection of oral health information from schoolteachers and covers oral health knowledge, attitudes and practices, sources of oral health information and teaching activities in classrooms.

Finally, separate WHO oral health questionnaires are available for people affected by HIV/AIDS, children with infections, and the role of schoolteachers. Such questionnaires may be relevant to carry out in conjunction with clinical examination of oral manifestations of HIV/AIDS.

2.2 From surveys to surveillance

Surveillance provides on going – continuous or periodic – collection, analysis and interpretation of population health data and the timely dissemination of such data to users (*8*). Properly conducted, surveillance ensures that decision-makers and public health administrators have the information they need to control disease now or plan strategies to prevent disease and adverse health events in the future. A systematic approach to data collection helps countries to observe and evaluate emerging disease patterns and trends. Operational indicators and targets are a prerequisite to policy formulation and surveillance. In sum, the goal of data collection is to assist governments, health authorities and health professionals in formulating policies, specifying standards and developing programmes to prevent disease, and to measure the progress, impact and efficacy of efforts to control diseases that are already affecting their populations.

Effective oral health surveillance requires well-defined oral health outcome indicators, indicators on self-assessment of oral health and care, important risks factors, and health systems response. Oral health indicators for use in public health need to meet certain criteria. That is, the information must cover the oral health conditions relevant to the public health burden of disease in a way that it is:

1. measurable and robust;
2. easy to understand;
3. relevant to quality of life;
4. clearly related to common modifiable risk factors; and
5. instrumental to oral disease prevention and promotion of oral health through health systems response.

The present oral health survey manual provides key instruments for assessment of the major components of the burden of oral disease and subsequent conditions. For example, tooth loss is the ultimate outcome of lifelong experience of severe dental caries and severe periodontal disease and greatly affects quality of life of people in terms of reduced functional capacity (e.g. chewing and biting), self-esteem and social relationships. A range of indicators may be

identified for policy formulation, setting of targets and surveillance at global, regional and national levels. Relevant indicators for use in oral health surveillance must be linked to standard age groups, such as:

- relative increase in the percentage of children aged 5 or 6 years old who are free of dental caries;
- relative reduction in the average DMFT among children aged 12 years;
- relative reduction in the percentage of older people (65–74 years) with complete loss of natural teeth;
- relative increase in the percentage of older people (65–74 years) who have a functional dentition (20 or more natural teeth).

Information on the number of natural teeth present in adults may also be collected through the questionnaire survey, if this is undertaken as a stand-alone survey.

Public health administrators may wish to formulate additional health indicators relevant to surveillance of community-specific oral health programmes, for example, indicators on periodontal disease (modified CPI) and incidence of oral cancer.

The STEPS questionnaire provides for specification of indicators for surveillance of self-assessed oral health, experience of oral health problems, self-care practices, quality of life, oral health risk factors such as dietary sugar intake and tobacco use, and the use of oral health services. Such indicators are important to countries in the evaluation of programmes focusing on the behavioural dimensions of oral health.

The general WHO STEPwise manual is designed to produce national surveillance data on main chronic diseases, self-assessment of health and common risk factors. Such data create the basis for global health surveillance and inter-country comparisons. WHO recommends collection of global surveillance data that may inform about people's experience of poor quality of life in relation to their oral health, whether primary oral health care is available and accessible, and whether health systems respond to severe oral conditions. This may be based on indicators such as:

- relative reduction in the percentage of children aged 5, 6 and 12 years and adolescents aged 15 years who report poor quality of life due to pain, discomfort or problems with mouth/teeth;
- relative increase in the percentage of children aged 5, 6 and 12 years and adolescents aged 15 years with access to primary oral health-care services;
- relative reduction in the percentage of adults (35–44 and 65–74 years) who report poor quality of life due to pain, discomfort or problems with mouth/teeth;

- relative increase in the percentage of adults (35–44 and 65–74 years) with access to primary oral health- care services;
- relative increase in the percentage of people in high risk groups (e.g. tobacco, betel quid users, and excessive alcohol users) screened for oral cancer at least once.

In general, surveillance of oral health is neglected in modern public health. However, oral health surveillance can successfully be incorporated into national health surveillance schemes as measurement of just a few, crucial indicators could provide sufficient information for valuable assessment of the appropriateness of public oral health intervention. This manual advocates close adherence to standardized approaches to oral health surveys, as this will aid WHO in storing consistently comparable, essential data in the Global Oral Health Data Bank.

Section 3: Obtaining assistance from WHO

3.1 Pre-survey assistance

WHO attaches great importance to basic oral health surveys for planning, evaluation and surveillance of oral health programmes and, whenever possible, the Organization offers assistance directly or through one of its collaborating centres or consultants.

WHO may assist with survey planning, including advice on the sampling plan, estimates of sample size and the appropriate use of standard assessment forms. The aims of such assistance are to foster the use of uniform survey methods and to help investigators develop objectives and survey plans to meet their specific needs. Prior to seeking assistance from WHO, investigators might find it helpful to discuss the survey and the proposed survey plan with experienced colleagues in the national health or education sector so that factors of importance and interest are not neglected or overlooked. When seeking pre-survey assistance from WHO, investigators are requested to provide the following information:

- name and address of the principal investigator;
- purpose and objectives of the planned survey;
- area(s) and region(s) to be surveyed;
- specifications of the target population, e.g. the number or percentage of the school-age population and the number or percentage of those who attend school;.
- age(s) of the population to be surveyed, e.g. children, adults, older people or all age groups;
- whether probability sampling is intended and, if so, the sampling frame from which the sample would be selected;
- whether a pathfinder approach will be used;
- estimates of the levels of dental caries, periodontal disease or other oral conditions for the ages under consideration (copies of reports from previous surveys should be provided if available);
- important subgroups within the population, e.g. ethnic groups, poor and disadvantaged groups, including the population ratio of such groups; and
- whether a risk factor questionnaire survey is planned, either as a stand-alone survey or in conjunction with clinical examination.

Assistance in training and calibration of potential oral examiners may, under certain circumstances, be available from WHO. Subject to prior agreement, an experienced epidemiologist who has been trained in the recommended methods for basic oral health surveys may be appointed to attend the training and calibration sessions as a trainer and validating examiner.

3.2 Post-survey assistance

Subject to prior agreement, WHO will assist, guide and facilitate use of a data entry programme in Epi Info or SPSS (Statistical Package for the Social Sciences) for analysis of data obtained through the procedures recommended in this manual, provided that the standardized format and coding have been used. The analysis may be carried out using a standard computer programme which will produce a standard set of tables (see **Annex 9** and **Annex 10**). The summarized data will be systematically included in the WHO Global Oral Health Data Bank.

For investigators who do not have access to computer facilities, WHO may be able to arrange assistance, for example, through a WHO Collaborating Centre.

Generated variables

As regards dental caries, the prevalence and severity of disease should be reported in terms of the standard epidemiological indicators:

- Age-specific proportion of persons with one or more untreated decayed lesions (D>0; d>0) and with caries experience (DMF>0; dmf>0). The DMF and dmf values of 0 are equivalent to a caries-free state. This is applicable to both the permanent and the primary dentitions.
- Age-specific means and measures of variation (e.g. standard deviation or standard error of means) for the following selected indicators of caries severity:
  - dt (number of decayed teeth in the primary dentition)
  - mt (number of teeth missing due to caries in the primary dentition)
  - ft (number of filled teeth in the primary dentition)
  - dft (number of decayed and filled teeth in the primary dentition)
  - dmft (number of decayed, missing due to caries and filled teeth in the primary dentition)

DT	(number of Decayed Teeth in the permanent dentition)
MT	(number of Missing Teeth due to caries in the permanent dentition)
FT	(number of Filled Teeth in the permanent dentition)
DFT	(number of Decayed and Filled Teeth in the permanent dentition)
DMFT	(number of Decayed, Missing due to caries, and Filled Teeth in the permanent dentition)

The DFT index can also be calculated for tooth roots since these data are obtained from each individual tooth; this is of particular importance in the age group of 65–74 years.

When the data have been collected by tooth surface (see **Annex 3** and **Annex 4**), a second set of tables with the corresponding indicators should be prepared.

- Age-specific contribution of each component to total caries index among individuals examined:
  - Per cent D/DMFT or d/dmft (per cent of decayed teeth within total caries experience index).
  - Per cent M/DMFT or m/dmft (per cent of teeth missing due to caries within total caries experience index).
  - Per cent F/DMFT or f/dmft (per cent of filled teeth within total caries experience index).
- The level of caries experience in the primary or permanent dentition may follow the WHO severity criteria (*5*). For the typical indicator age groups of children (12 years) and adults (35–44 years), the following population levels of DMFT may be considered for summarizing the degree of caries experience:

**Children 12 years of age (DMFT)**	**Adults 35–44 years of age (DMFT)**
Very low <1.2	Very low <5.0
Low 1.2–2.6	Low 5.0–8.9
Moderate 2.7–4.4	Moderate 9.0–13.9
High 4.5–6.5	High >13.9
Very high >6.5	

Likewise, the level of dental caries among 5/6-year-olds (dmft) and 65–74 year-olds (DMFT) may be described on the basis of categories of caries experience.

Summarizing the data on dental caries prevalence as indicated in this section should be carried out after stratification of the entire sample by:

- age group
- geographical sites/regions/units
- sex
- ethnic group.

If the study design requires recording dentition status by tooth surface, e.g. in evaluation of the effect of public health intervention programmes, this can be done using the forms provided in **Annex 3** and **Annex 4**. Corresponding categorization of the level of dental caries can then be derived.

Periodontal status (CPI) modified

The gingival health status by age group (children and adults) should be reported giving the number of individuals and percentage of individuals who had no bleeding on probing (score 0), and bleeding on probing (score 1). The gingival health status is described by the number and percentage of teeth present with bleeding (score 1), respectively without bleeding (score 0). In addition, the number and percentage of teeth excluded, and the number and percentage of teeth not present should be reported.

The prevalence of adults with specific pocket scores is reported by age group using the following variables:

- the number and percentage of individuals with absence of condition (score 0);
- the number and percentage of individuals with pockets 4–5 mm (score 1); and
- the number and percentage of individuals with pockets 6 mm or more (score 2).

Severity of periodontal disease is indicated by the following variables (*23*):

- the number and percentage of teeth present with absence of condition (score 0);
- the number and percentage of teeth with pockets 4–5 mm (score 1); and
- the number and percentage of teeth with pockets 6 mm or more (score 2).

In addition, the number and percentage of teeth excluded, and the number and percentage of teeth not present should be reported.

The CPI modified system includes recording of signs of periodontal disease in all the teeth that are present. However, if an investigator is interested in comparing his or her actual findings with those of surveys in which former versions of the CPI system were applied, the analysis may be restricted to indicator teeth only.

Questionnaire data analysis

Complete analysis of answers to the questions on oral health related quality of life (Q12), consumption of sugary foods and drinks (Q 13), consumption of tobacco (Q 14), and harmful use of alcohol (Q15) will require derivate variables. Q10, Q11, and Q12 are corresponding questions in the questionnaire to children. Behavioural indices may be generated from these questions by addition of the item scores chosen by the respondent (*22*). Cut-off points for categories (3 or 5) may then be determined from empirical distributions.

3.3 Preparation of survey reports

The report of the survey must contain the following information:

1. *Statement of the purposes of the survey*: This statement should include a succinct and clear description of the aims of the survey and the expected ways in which the results will be used.
2. *Materials and methods*: Under this heading, it is customary to include the following:
   - *Area and population*: This section will include a general description of the geographical region and the sample of people examined.
   - *The nature of the information collected and the methods used*: A description is required of the type of information collected and of the methods used to collect the data, e.g. by self-administered questionnaire, interview or clinical examination. It is also essential to indicate the year of data collection. If reference is made to methods outlined in this manual, it may not be necessary to describe the clinical examination in detail.
   - *Sampling method*: An explanation should be given of the method of sampling that was used, the size of the total sample and sub-samples, response rate, and the extent to which the sample is considered representative of the target population. The report should describe the criteria used to group the different elements into urban, periurban and rural clusters, and description of the process for selecting schools, classrooms, students, adults, older people, etc. The number and description of persons who were selected for the sample but were not examined, and any sampling problems encountered, should also be reported. If probability sampling was used, the report should explain how the final sample was attained, e.g. stratified sampling, systematic sampling or probability proportional to size. Also, it is important to indicate whether any weighting was included in the analysis of the data as well as the computer software used for this purpose, if any.

- *Personnel and physical arrangements*: It is advisable to give a brief account of the physical arrangements at the examination sites, the equipment used, and the organization, training and experience of the personnel employed in collecting, processing and tabulating the data. Arrangements made for standardization and calibration of examiners and interviewers and for checking the consistency of examiners during the course of the survey should be described.
- *Statistical analysis and computational procedure*: The statistical methods used in compiling the final summary tables from the raw data should be described briefly or appropriate references provided. Reference can be made, for example, to methods described in this manual, where relevant. If data analyses were conducted at the country level, information on computer software (e.g. Epi Info, SPSS) used for this purpose should be provided in the report.
- *Cost analysis*: Information on survey expenses and the length of time spent on data collection is of considerable interest. Reporting the costs of planning, training and calibration exercises, field work, supervision, statistical analysis, salaries and overheads facilitates the critical evaluation of survey methods and provides useful economic data.
- *Reliability and reproducibility of the results*: It is important to include data on inter-examiner and intra-examiner variability as revealed by pre-survey calibration examinations and duplicate examinations conducted during the course of the survey. This information gives the planner for the area and the reader of the report an indication of the degree of examiner error that may apply to any of the results. Oral health survey reports should include a description of the calibration activities, including how and when the calibration process was carried out, how many examiners and recorders were trained, the variables included in the calibration, and the individual and overall (all examiners) per cent agreements and corrected kappa statistics for intra- and inter-examiner reliability.

3. *Results*: Results may be presented in several ways. Brevity is important. The text should contain a short description of the more important results and summary tables. A few diagrams, graphs, histograms, bar charts or pie charts may be used to illustrate points that are neither easily explained in the text nor easily visualized from the tables. A cardinal rule for both figures and tables is that they should be clearly labelled so that they are comprehensible without reference to the text.

The basic summary tables provided in the WHO standard programme address two main areas: (a) oral health status and intervention urgency (**Annex**

9), and (b) self-assessed oral health and risks (**Annex 10**). A comprehensive report of the results should include self-explanatory and independent tables with totals at the bottom of the table.

Age-specific tables should be produced for other conditions included in the survey; for example, if the survey obtained data for prevalence of oral mucosal lesions, these variables need to be described for each group and then by geographical location, sex and ethnicity, if appropriate. Where categorical observations have been recorded, the table should include the number and the per cent for each category. This applies to several clinical conditions and responses in the questionnaires.

In general, questionnaire data generate categorical or ordinal variables. The number of responses to each individual question should be calculated and the percentages given in standard tables. In addition to simple univariate and bivariate distributions, the data set allows stratified and multivariate analyses based on frequency distributions; analysis requiring more complex associations will require use of regression analysis techniques, and these are included in standard statistical tools such as SPSS or SAS. The use of such tools is also pertinent where clinical and questionnaire data sets have been merged.

4. *Discussion and conclusions*: The results of the survey should be discussed systematically.
   - The *oral health status* of the population should be compared with data from previous surveys of a similar population if such data are available. In addition, comparison of the actual findings may be made with results of surveys of similar age groups in neighbouring settings or countries.
   - *Shortcomings of the survey* should describe the aspects of the survey that could have been done better.
   - The *implications of survey results* for oral health promotion and integrated disease prevention, development or adjustment of programmes should be clarified, and suggestions made for practical organization of programmes within the context of a national public health system.
   - *Intervention urgency* of the population examined should be reported together with a brief discussion of the different intervention categories for the age groups concerned.
5. *Summary or abstract*: A brief summary of the report is required, of a suitable length for use as an abstract (approximately 250 words). The objectives of the study and the number of people examined should be stated, and a few of the more important results given for dentition status and periodontal health or other oral conditions in two or three age groups and for the whole sample; for example, the proportion of subjects affected by caries and the proportion of teeth with gingival

bleeding and pocketing. The proportion of subjects in need of prompt and urgent intervention may be included. Any unusual or unexpected results should be noted. Depending on survey conditions, findings from the sample may be generalizable at the country level and public health recommendations should be made accordingly.

References

1. *Basic Oral Health Survey Methods*, 1st ed. Geneva, World Health Organization, 1971.
2. *Oral health surveys. Basic methods*, 2nd ed. Geneva, World Health Organization, 1977.
3. *Oral health surveys. Basic methods*, 3rd ed. Geneva, World Health Organization, 1987.
4. *Oral Health Survey. Basic methods*, 4th ed. Geneva, World Health Organization, 1997.
5. *The World Oral Health Report 2003: Continuous improvement of oral health in the 21st century – the approach of the WHO Global Oral Health Programme*. Geneva, World Health Organization, 2003.
6. Petersen PE. Oral health. In: Heggenhaugen K, Quah S, eds. *International encyclopedia of public health, Vol. 4*. San Diego, CA, Academic Press, 2008:677–685.
7. Petersen PE. Socio-behavioural risk factors in dental caries – international perspectives. *Community Dentistry and Oral Epidemiology*, 2005, 33:274–279.
8. Petersen PE et al. Oral health information systems – towards measuring progress in oral health promotion and disease prevention. *Bulletin of the World Health Organization*, 2005, 83:686–693.
9. *WHO STEPS Surveillance Manual: The WHO STEPwise approach to chronic disease risk factor surveillance*. Geneva, World Health Organization, 2005.
10. Armitage P, Berry G, Matthews JNS. *Statistical methods in medical research*, 4th ed. Oxford, Blackwell Science, 2002.
11. Lesaffre E et al. Statistical and methodological aspects of oral health research. Chichester, John Wiley & Sons Ltd, 2009.
12. Eklund S, Moller IJ, LeClercq MH. Calibration of examiners for oral epidemiological surveys. World Health Organization, 1993 (ORH/EIS/EPID.93.1).
13. Bulman JS, Osborne JF. Measuring diagnostic consistency. *British Dental Journal*, 1989, 166:377–381.
14. Landis JR, Kock GG. The measurement of observer agreement for categorical data. *Biometrics*, 1977, 33:159–174.

15. Cuny E et al. *Infection control in practice – oral health care*. WHO Collaborating Centre for Community Oral Health Programmes and Research; Organization for Safety and Asepsis Procedures (OSAP); University of Copenhagen, Faculty of Health Sciences, Copenhagen, 2010.

16. Baez RJ, Baez XL. Use of pressure cookers for sterilization of clinical instruments. *Journal of Dental Research*, 1998, 77:Abstract 1774.

17. Melnick SL et al. *A guide for epidemiological studies of oral manifestations of HIV infection*. Geneva, World Health Organization, 1993.

18. ISO TC106 - ISO 3950:2009 *Dentistry – Designation system for teeth and areas of the oral cavity*.

19. *WHO Expert Group on Equipment and Materials for Oral Care (EGEMOC). The periodontal probe for use with the Community Periodontal Index of Treatment Needs*. Geneva, World Health Organization, 1990 (unpublished document available on request from Oral Health, World Health Organization, 1211 Geneva 27, Switzerland).

20. Dean HT. The investigation of physiological effects by the epidemiological method. In: Moulton FR, ed. *Fluoride and dental health*. Washington, DC, American Association for the Advancement of Science, 1942, (Publication No. 19):23–31.

21. *Health Interview Surveys – Towards international harmonization of methods and instruments*. Copenhagen, WHO Regional Office for Europe, 1996.

22. Bryan A. *Social research methods*. Oxford, Oxford University Press, 2012 (Online Resource Centres).

23. Petersen PE, Ogawa H. The global burden of periodontal disease. *Periodontol 2000*, 2012, 60:15–39.

World Health Organization

Oral Health Assessment Form for Adults, 2013

Leave blank	Year	Month	Day	Identification No.	Orig/Dupl	Examiner
(1) (4)	(5)		(10)	(11) (14)	(15)	(16) (17)

General information: **Sex** 1=M, 2=F (18) **Date of birth** (19) (24) **Age in years** (25) (26)

(Name)

Ethnic group (27) (28) **Other group**(29) (30) **Years in school** (31) (32) **Occupation** (33)

Community (geographical location) (34) (35) **Location** Urban (1) Periurban (2) Rural (3) (36)

Other data (37) (38) **Other data** (39) (40)

Other data (41) (42) **Extra-oral examination** (43) (44)

Dentition status

		18	17	16	15	14	13	12	11	21	22	23	24	25	26	27	28	
Crown	(45)																	60)
Root	(61)																	(76)
Crown	(77)																	(92)
Root	(93)																	(108)
		48	47	46	45	44	43	42	41	31	32	33	34	35	36	37	38	

Permanent teeth

Status

- 0 = Sound
- 1 = Caries
- 2 = Filled w/caries
- 3 = Filled, no caries
- 4 = Missing due to caries
- 5 = Missing for any another reason
- 6 = Fissure sealant
- 7 = Fixed dental prosthesis/crown abutment, veneer, implant
- 8 = Unerupted
- 9 = Not recorded

Periodontal status (CPI Modified)

		18	17	16	15	14	13	12	11	21	22	23	24	25	26	27	28	
Bleeding	(109)																	(124)
Pocket	(125)																	(140)
Bleeding	(141)																	(156)
Pocket	(157)																	(172)
		48	47	46	45	44	43	42	41	31	32	33	34	35	36	37	38	

Gingival bleeding

Score

- 0 = Absence of condition
- 1 = Presence of condition
- 9 = Tooth excluded
- X = Tooth not present

Pocket

Score

- 0 = Absence of condition
- 1 = Pocket 4–5 mm
- 2 = Pocket 6 mm or more
- 9 = Tooth excluded
- X = Tooth not present

World Health Organization
Oral Health Assessment Form for Adults, 2013

Loss of attachment

Index teeth

(173) 17/16	11	26/27 (175)
(176) 47/46	31	36/37 (178)

Severity
0 = 0–3 mm
1 = 4–5 mm Cemento-enamel junction (CEJ) within black band
2 = 6–8 mm CEJ between upper limit of black band and 8.5 mm ring
3 = 9–11 mm CEJ between 8.5 mm and 11.5 mm ring
4 = 12 mm or more CEJ beyond 11.5 mm ring
X = Excluded sextant
9 = Not recorded

* Not recorded under 15 years of age

Enamel fluorosis (179)

Severity
0 = Normal
1 = Questionable
2 = Very mild
3 = Mild
4 = Moderate
5 = Severe
8 = Excluded (crown, restoration, "bracket")
9 = Not recorded (unerupted tooth)

Dental erosion

Severity (180)

0 = No sign of erosion
1 = Enamel lesion
2 = Dentinal lesion
3 = Pulp involvement

Number of teeth affected

(181) (182)

Dental trauma

Status (183)

0 = No sign of injury
1 = Treated injury
2 = Enamel fracture only
3 = Enamel and dentine fracture
4 = Pulp involvement
5 = Missing tooth due to trauma
6 = Other damage
9 = Excluded tooth

Number of teeth affected

(184) (185)

Oral mucosal lesions

(186) (189)
(187) (190)
(188) (191)

Condition

0 = No abnormal condition
1 = Malignant tumour (oral cancer)
2 = Leukoplakia
3 = Lichen planus
4 = Ulceration (aphthous, herpetic, traumatic)
5 = Acute necrotizing ulcerative gingivitis (ANUG)
6 = Candidiasis
7 = Abscess
8 = Other condition (specify if possible)
9 = Not recorded

Location

0 = Vermillion border
1 = Commissures
2 = Lips
3 = Sulci
4 = Buccal mucosa
5 = Floor of the mouth
6 = Tongue
7 = Hard and/or soft palate
8 = Alveolar ridges/gingiva
9 = Not recorded

Denture(s)

Upper (192) **Lower** (193)

Status

0 = No denture
1 = Partial denture
2 = Complete denture
9 = Not recorded

Intervention urgency (194)
0 = No treatment needed
1 = Preventive or routine treatment needed
2 = Prompt treatment (including scaling) needed
3 = Immediate (urgent) treatment needed due to pain or infection of dental and/or oral origin
4 = Referred for comprehensive evaluation or medical/dental treatment (systemic condition)

World Health Organization

World Health Organization
Oral Health Assessment Form for Children, 2013

Leave blank	Year	Month	Day	Identification No.	Orig/Dupl	Examiner
(1) ☐☐☐☐ (4)	(5) ☐☐	☐☐	☐☐ (10)	(11) ☐☐☐☐ (14)	☐ (15)	(16) ☐☐ (17)

General information: **Sex** 1=M, 2=F ☐ (18) **Date of birth** (19) ☐☐☐☐☐☐ (24) **Age in years** (25) ☐☐ (26)

____
(Name)

Ethnic group (27) ☐☐ (28) **Other group** (29) ☐☐ (30) **Years in school** (31) ☐☐ (32) **Occupation** ☐ (33)

Community (geographical location) (34) ☐☐ (35) **Location** Urban (1) Periurban (2) Rural (3) ☐ (36)

Other data ____________ (37) ☐☐ (38) **Other data** ____________ (39) ☐☐ (40)

Other data ____________ (41) ☐☐ (42) **Extra-oral examination** ______ (43) ☐☐ (44)

Dentition status

			55	54	53	52	51	61	62	63	64	65			
	17	16	15	14	13	12	11	21	22	23	24	25	26	27	
Crown (45)	☐	☐	☐	☐	☐	☐	☐	☐	☐	☐	☐	☐	☐	☐	(58)
Crown (59)	☐	☐	☐	☐	☐	☐	☐	☐	☐	☐	☐	☐	☐	☐	(72)
			85	84	83	82	81	71	72	73	74	75			
	47	46	45	44	43	42	41	31	32	33	34	35	36	37	

Primary teeth	Permanent teeth Status
A	0 = Sound
B	1 = Caries
C	2 = Filled w/caries
D	3 = Filled, no caries
E	4 = Missing due to caries
—	5 = Missing for any another reason
F	6 = Fissure sealant
G	7 = Fixed dental prosthesis/crown, abutment, veneer
—	8 = Unerupted
—	9 = Not recorded

Periodontal status

			55	54	53	52	51	61	62	63	64	65			
	17	16	15	14	13	12	11	21	22	23	24	25	26	27	
(73)	☐	☐	☐	☐	☐	☐	☐	☐	☐	☐	☐	☐	☐	☐	(86)
(87)	☐	☐	☐	☐	☐	☐	☐	☐	☐	☐	☐	☐	☐	☐	(100)
			85	84	83	82	81	71	72	73	74	75			
	47	46	45	44	43	42	41	31	32	33	34	35	36	37	

Gingival bleeding

Scores
0 = Absence of condition
1 = Presence of condition
9 = Tooth excluded
X = Tooth not present

Enamel fluorosis ☐ (101)

Status
0 = Normal
1 = Questionable
2 = Very mild
3 = Mild
4 = Moderate
5 = Severe
8 = Excluded (crown, restoration, "bracket")
9 = Not recorded (unerupted tooth)

Dental erosion

Severity

(102) ☐

0 = No sign of erosion
1= Enamel lesion
2 = Dentinal lesion
3 = Pulp involvement

No. of teeth
(103) ☐☐ (104)

Dental trauma

Status

(105) ☐

0 = No sign of injury
1 = Treated injury
2 = Enamel fracture only
3 = Enamel and dentine fracture
4 = Pulp involvement
5 = Missing tooth due to trauma
6 = Other damage
9 = Excluded tooth

No. of teeth
(106) ☐☐ (107)

Oral mucosal lesions

Condition
(108) ☐
(109) ☐
(110) ☐

Location
(111) ☐
(112) ☐
(113) ☐

0 = No abnormal condition
1 = Ulceration (aphthous, herpetic, traumatic)
2 = Acute necrotizing ulcerative gingivitis (ANUG)
3 = Candidiasis
4 = Abscess
8 = Other condition
9 = Not recorded

0 = Vermillion border
1 = Commissures
2 = Lips
3 = Sulci
4 = Buccal mucosa
5 = Floor of mouth
6 = Tongue
7 = Hard and/or soft palate
8 = Alveolar ridges/gingiva
9 = Not recorded

Intervention urgency ☐ (114)

0 = No treatment needed
1 = Preventive or routine treatment needed
2 = Prompt treatment (including scaling) needed
3 = Immediate (urgent) treatment needed due to pain or infection of dental and/or oral origin
4 = Referred for comprehensive evaluation or medical/dental treatment (systemic condition)

World Health Organization

Oral Health Assessment Form for Adults (by tooth surface), 2013

Leave blank (1) ____ (4) | Year Month Day (5) ____ (10) | Identification No. (11) ____ (14) | Orig/Dupl (15) | Examiner (16) ____ (17)

General information: **Sex** 1=M, 2=F (18) | **Date of birth** (19) ____ (24) | **Age in years** (25) ____ (26)

____
(Name)

Ethnic group (27) ____ (28) **Other group** (29) ____ (30) **Years in school** (31) ____ (32) **Occupation** ____ (33)

Community (geographical location) (34) ____ (35) **Location** Urban (1) Periurban (2) Rural (3) ____ (36)

Other data ____________________ (37) ____ (38) **Other data** ____________________ (39) ____ (40)

Other data ____________________ (41) ____ (42) **Extra-oral examination** __________ (43) ____ (44)

Dentition status by tooth surface

	18	17	16	15	14	13	12	11	21	22	23	24	25	26	27	28	
Occ																	(45–54)
Mes																	(55–70)
Buc																	(71–86)
Dis																	(87–102)
Oral																	(103–118)

	48	47	46	45	44	43	42	41	31	32	33	34	35	36	37	38	
Occ																	(119–128)
Mes																	(129–144)
Buc																	(145–160)
Dis																	(161–176)
Oral																	(177–192)

Permanent teeth

Status

0 = Healthy
1 = Caries
2 = Filled w/caries
3 = Filled no/caries
4 = Missing due to caries
5 = Missing for another reason
6 = Fissure sealant
7 = Fixed partial denture,/crown, abutment, veneer, implant
8 = Unerupted
9 = Not recorded

Periodontal status (CPI Modified)

	18	17	16	15	14	13	12	11	21	22	23	24	25	26	27	28	
Bleeding (193)																	(208)
Pocket (209)																	(224)
Bleeding (225)																	(240)
Pocket (241)																	(256)
	48	47	46	45	44	43	42	41	31	32	33	34	35	36	37	38	

Gingival bleeding

Score

0 = Absence of condition
1 = Presence of condition
9 = Tooth excluded
X = Tooth not present

Pocket

Score

0 = Absence of condition
1 = Pocket 4–5 mm
2 = Pocket 6 mm or more
9 = Tooth excluded
X = Tooth not present

World Health Organization

World Health Organization
Oral Health Assessment Form for Adults, 2013

Loss of attachment

Severity
0 = 0–3 mm
1 = 4–5 mm Cemento-enamel junction (CEJ) within black band
2 = 6–8 mm CEJ between upper limit of black band and 8.5 mm ring
3 = 9–11 mm CEJ between 8.5 mm and 11.5 mm ring
4 = 12 mm or more CEJ beyond 11.5 mm ring
X = Excluded sextant
9 = Not recorded

* Not recorded under 15 years of age

Index teeth

	17/16	11	26/27	
(257)				(259)
(260)				(262)
	47/46	31	36/37	

Enamel fluorosis (263)

Severity
0 = Normal
1 = Questionable
2 = Very mild
3 = Mild
4 = Moderate
5 = Severe
8 = Excluded (crown, restoration, "bracket")
9 = Not recorded (unerupted tooth)

Dental erosion

Severity (264)

0 = No sign of erosion
1 = Enamel lesion
2 = Dentinal lesion
3 = Pulp involvement

Number of teeth affected

(265) (266)

Dental trauma

Status (267)

0 = No sign of injury
1 = Treated injury
2 = Enamel fracture only
3 = Enamel and dentine fracture
4 = Pulp involvement
5 = Missing tooth due to trauma
6 = Other damage
9 = Excluded tooth

Number of teeth affected

(268) (269)

Oral mucosal lesions

(270)
(271)
(272)

(273)
(274)
(275)

Condition

0 = No abnormal condition
1 = Malignant tumour (oral cancer)
2 = Leukoplakia
3 = Lichen planus
4 = Ulceration (aphthous, herpetic, traumatic)
5 = Acute necrotizing ulcerative gingivitis (ANUG)
6 = Candidiasis
7 = Abscess
8 = Other condition (specify if possible)
9 = Not recorded

Location

0 = Vermillion border
1 = Commissures
2 = Lips
3 = Sulci
4 = Buccal mucosa
5 = Floor of the mouth
6 = Tongue
7 = Hard and/or soft palate
8 = Alveolar ridges/gingiva
9 = Not recorded

Denture(s)

Upper (276) **Lower** (277)

Status

0 = No denture
1 = Partial denture
2 = Complete denture
9 = Not recorded

Intervention urgency (278)

0 = No treatment needed
1 = Preventive or routine treatment needed
2 = Prompt treatment (including scaling) needed
3 = Immediate (urgent) treatment needed due to pain or infection of dental and/or oral origin
4 = Referred for comprehensive evaluation or medical treatment (systemic condition)

World Health Organization

Oral Health Assessment Form for Children (by tooth surface), 2013

Leave blank	Year	Month	Day	Identification No.	Orig/Dupl	Examiner
(1) ☐☐☐☐ (4)	(5) ☐☐	☐☐	☐☐ (10)	(11) ☐☐☐☐ (14)	☐ (15)	(16) ☐☐ (17)

General information:

Sex 1=M, 2=F ☐ (18)

Date of birth (19) ☐☐☐☐☐☐ (24)

Age in years (25) ☐☐ (26)

____
(Name)

Ethnic group (27) ☐☐ (28) **Other group** (29) ☐☐ (30) **Years in school** (31) ☐☐ (32) **Occupation** ☐ (33)

Community (geographical location) (34) ☐☐ (35)

Location Urban (1) Periurban (2) Rural (3) ☐ (36)

Other data ______________________ (37) ☐☐ (38)

Other data ______________________ (39) ☐☐ (40)

Other data ______________________ (41) ☐☐ (42)

Extra-oral examination _________ (43) ☐☐ (44)

Dentition status by tooth surface

			55	54	53	52	51	61	62	63	64	65			
	17	16	15	14	13	12	11	21	22	23	24	25	26	27	
Occ					X	X	X	X	X	X					(45–52)
Mes															(53–66)
Buc															(67–80)
Dis															(81–94)
Oral															(95–108)

			85	84	83	82	81	71	72	73	74	75			
	47	46	45	44	43	42	41	31	32	33	34	35	36	37	
Occ					X	X	X	X	X	X					(109–116)
Mes															(117 –130)
Buc															(131–144)
Dis															(145–158)
Oral															(159–172)

Primary teeth	Permanent teeth
	**Status**
A	0 = Sound
B	1 = Caries
C	2 = Filled w/caries
D	3 = Filled, no caries
E	4 = Missing due to caries
–	5 = Missing for another reason
F	6 = Fissure sealant
G	7 = Fix dental prosthesis/crown, abutment, veneer
–	8 = Unerupted
–	9 = Not recorded

World Health Organization

World Health Organization
Oral Health Assessment Form for Children, 2013

Periodontal status

			55	54	53	52	51	61	62	63	64	65			
	17	16	15	14	13	12	11	21	22	23	24	25	26	27	
(173)															(186)
(187)															(200)
			85	84	83	82	81	71	72	73	74	75			
	47	46	45	44	43	42	41	31	32	33	34	35	36	37	

Gingival bleeding
Score
(0) = Absence of condition
(1) = Presence of condition
(9) = Tooth excluded
(X) = Tooth not present

Enamel fluorosis (201)

Severity

0 = Normal
1 = Questionable
2 = Very mild
3 = Mild
4 = Moderate
5 = Severe

8 = Excluded (crown, restoration, "bracket")

9 = Not recorded (unerupted tooth)

Dental erosion

Severity

(202)

0 = No sign of erosion
1 = Enamel lesion
2 = Dentinal lesion
3 = Pulp involvement

No. of teeth

(203) (204)

Dental trauma

Status

(205)

0 = No sign of injury
1 = Treated injury
2 = Enamel fracture only
3 = Enamel and dentine fracture
4 = Pulp involvement
5 = Missing tooth due to trauma
6 = Other damage
9 = Excluded tooth

No. of teeth

(206) (207)

Oral mucosal lesions

Condition

(208)
(209)
(210)

Location

(211)
(212)
(213)

Condition:
0= No abnormal condition
1= Ulceration (aphthous herpetic, traumatic)
2= Acute necrotizing ulcerative gingivitis (ANUG)
3= Candidiasis
4= Abscess
8= Other condition
9= Not recorded

Location:
0 = Vermillion border
1 = Commissures
2 = Lips
3 = Sulci
4 = Buccal mucosa
5 = Floor of mouth
6 = Tongue
7 = Hard/soft palate
8 = Alveolar ridges/gingiva
9= Not recorded

Intervention URGENCY (214)

0 = No curative treatment needed

1 = Preventive or routine treatment needed

2 = Prompt treatment (including scaling) needed

3 = Immediate (urgent) treatment due to pain or infection of dental and/or oral origin

4 = Referred for comprehensive evaluation or medical treatment (systemic condition)

World Health Organization

World Health Organization

Record Form for Oral Manifestations in HIV/AIDS, 2013

Country: ____________________

Leave blank (1) [ ][ ][ ][ ] (4)   Year Month Day (5) [ ][ ][ ][ ][ ][ ] (10)   Identification No. (11) [ ][ ][ ][ ] (14)   Orig/Dupl [ ] (15)   Examiner (16) [ ][ ] (17)

General information: ____________________ (Name)

Sex 1=M, 2=F [ ] (18)   **Date of birth** (19) [ ][ ][ ][ ][ ][ ] (24)   **Age in years** (25) [ ][ ] (26)

Ethnic group (27) [ ][ ] (28)   **Other group** (29) [ ][ ] (30)   **Years in school** (31) [ ][ ] (32)   **Occupation** [ ] (33)

Community (geographical location) (34) [ ][ ] (35)   **Location** Urban (1) Periurban (2) Rural (3) [ ] (36)

Other data ____________ (37) [ ][ ] (38)   **Other data** ____________ (39) [ ][ ] (40)

Other data ____________ (41) [ ][ ] (42)   **Other data** ____________ (43) [ ][ ] (44)

Extra-oral examination ________ (45) [ ][ ] (46)   **Extra-oral examination** ________ (47) [ ][ ] (48)

Weight in kg [ ][ ] (49–50)

Height in cm [ ][ ][ ] (51–53)

Fever [ ] (54)

1 = Present 2 = Absent

Candidiasis

1 = Present 2 = Absent

Erythematous [ ] (55)   **Hyperplastic** [ ] (56)   **Pseudomembranous** [ ] (57)

Location of lesion

1 = Present 2 = Absent

[ ] (58) **Tongue**   [ ] (59) **Gingiva**   [ ] (60) **Lip/buccal mucosa**   [ ] (61) **Palate**   [ ] (62) **Pharynx**

World Health Organization
Record Form for Oral Manifestations in HIV/AIDS, 2013

	1 = Present ✓ tick	2 = Absent ✓ tick	
Angular cheilitis	☐	☐	(63)
Oral hairy leukoplakia	☐	☐	(64)
Necrotizing ulcerative gingivitis (NUG)	☐	☐	(65)
Necrotizing ulcerative periodontitis (NUP)	☐	☐	(66)
Necrotizing stomatitis	☐	☐	(67)
Herpetic stomatitis/gingivitis and/or labial	☐	☐	(68)
Herpes zoster	☐	☐	(69)
Molluscum contagiosum	☐	☐	(70)
Cytomegalovirus	☐	☐	(71)
Warty-like lesions/human papillomavirus	☐	☐	(72)
Kaposi sarcoma	☐	☐	(73)
Aphthous ulcers	☐	☐	(74)
Other ulcerations	☐	☐	(75)
Dry mouth due to decreased salivary flow	☐	☐	(76)
Unilateral or bilateral swelling of major salivary glands	☐	☐	(77)
Other(s)	☐	☐	(78)

Atlas of major oral diseases and conditions

The images included in this annex complement the criteria and recommended codes that should be used while recording oral lesions and conditions found in clinical examinations

Acknowledgements

Scully C, Welbury R, Flaitz C, Paes de Almeida O. *Orofacial health and disease in children and adolescents*, 2nd ed. London: Martin Dunitz, 2002 (Plates 45, 51, 57, 60, 61, 64).

Scully C, Flint SR, Porter SR, Moos KF. *Oral and maxillofacial diseases*, 3rd ed. London: Informa Healthcare, 2008 (Plates 52–56, 58, 59, 62, 63).

Dr M. Valderrama (Plates 9, 22, 26, 27).

Dr R. Baez (Plates 1, 3–8, 15, 17–21, 23–25, 28, 36–42, 46–50, 65–71).

Dr G. Menghini /Dr R. Baez (Plates 29–35).

Dr P.E. Petersen (Plates 2, 11, 12, 16, 43, 44)

Dentition status

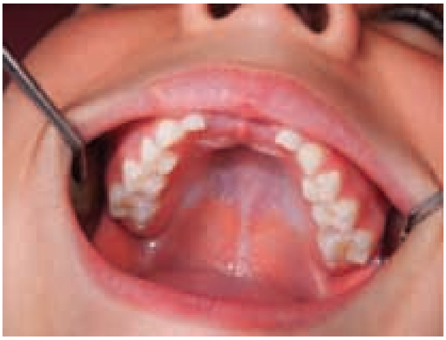

Plate 1

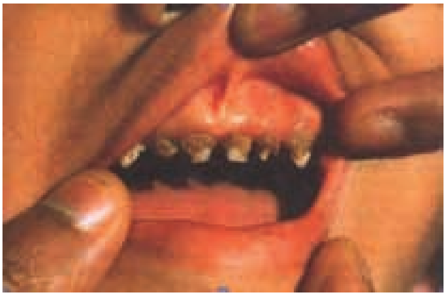

Plate 2

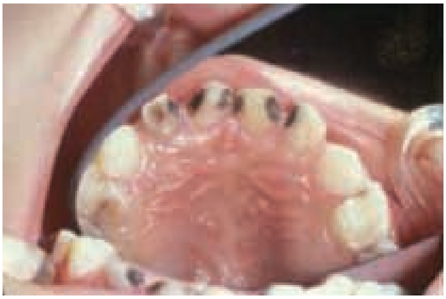

Plate 3

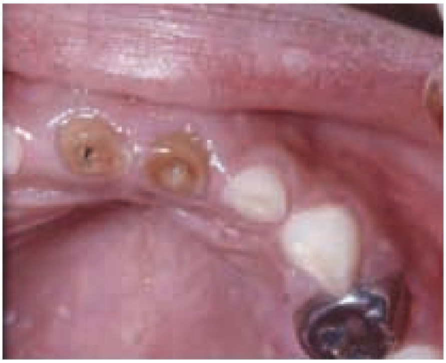

Plate 4

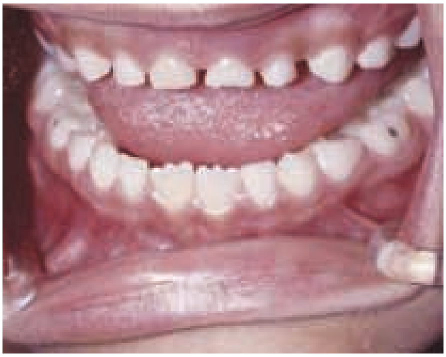

Plate 5

Explanatory notes

Plate 1 Code A: sound primary dentition; Code 8: unerupted maxillary incisors; and Code 0: sound first permanent molars

Plate 2 Code B: caries – primary dentition

Plate 3 Code B: caries – teeth 52, 51, 61 and 62

Plate 4 Code B: caries – teeth 51 and 61; code D: filled no caries – tooth 64

Plate 5 Code D: filled with no caries – teeth 75 and 85

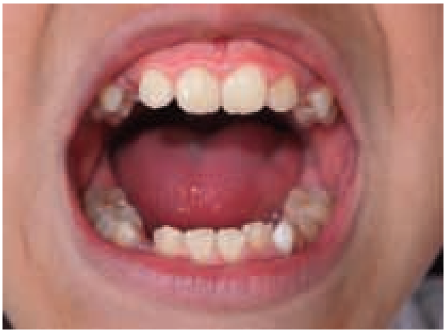

Plate 6

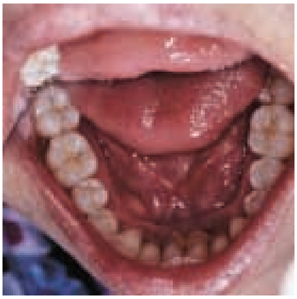

Plate 7

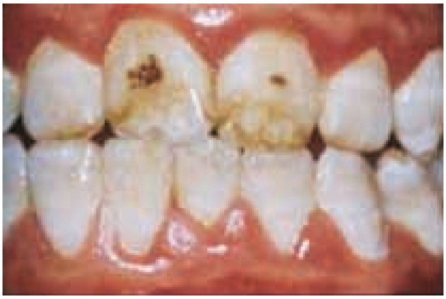

Plate 8

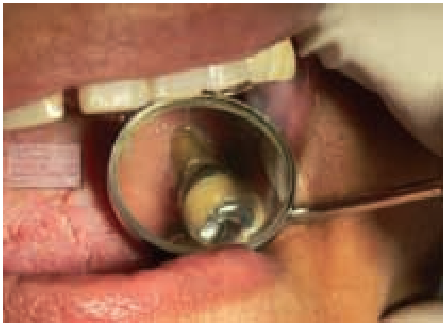

Plate 9

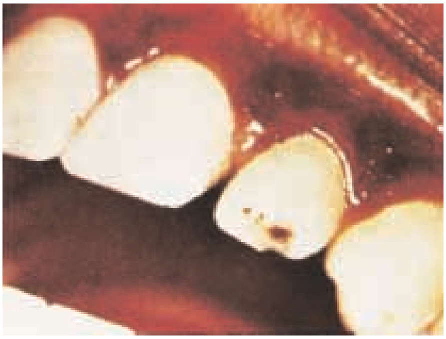

Plate 10

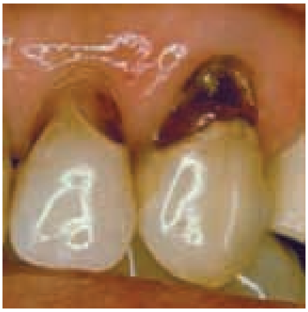

Plate 11

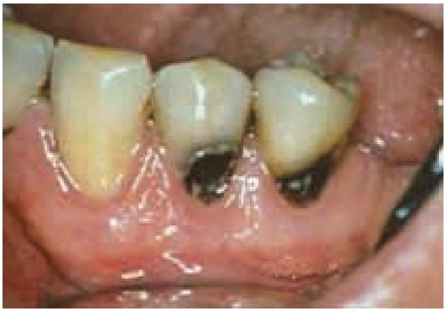

Plate 12

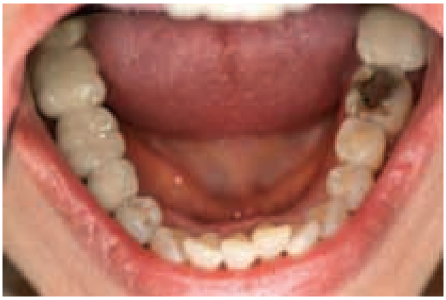

Plate 13

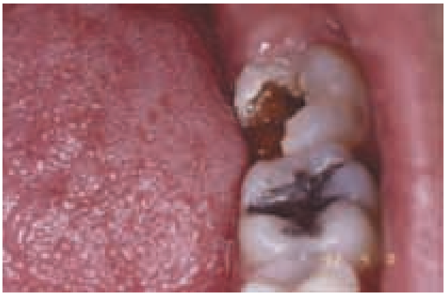

Plate 14

Explanatory notes

Plate 6 Code A and code 0: sound primary and permanent dentitions
Plate 7 Code 0: *Sound* stained pits or fissures in the enamel that do not have visual signs of undermined enamel
Plate 8 Code 0: sound crown with dark pitted areas/fluorosis
Plate 9 Code 0: sound root
Plate 10 Code 1: caries – permanent dentition
Plate 11 Code 1: caries – teeth 22 and 23
Plate 12 Code 1: caries – teeth 34 and 35
Plate 13 Code 2: filled with caries – tooth 36
Plate 14 Code 1: *Caries* Tooth 37; Code 3: *Filled with no decay* Tooth 36

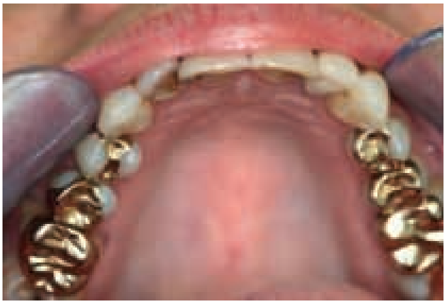

Plate 15

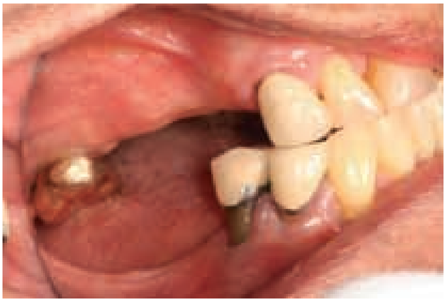

Plate 16

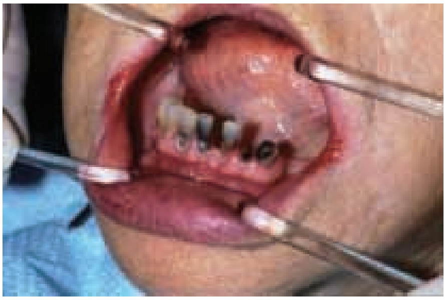

Plate 17

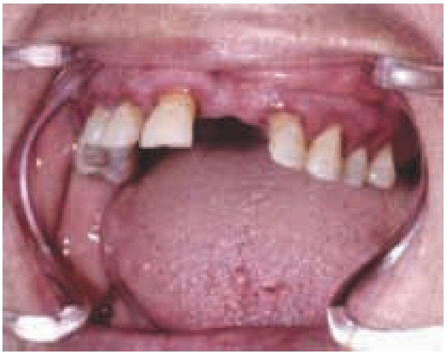

Plate 18

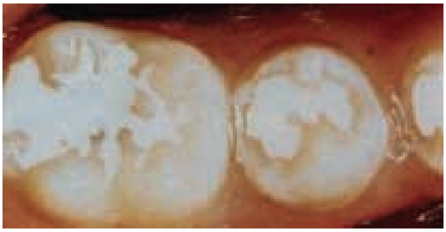

Plate 19

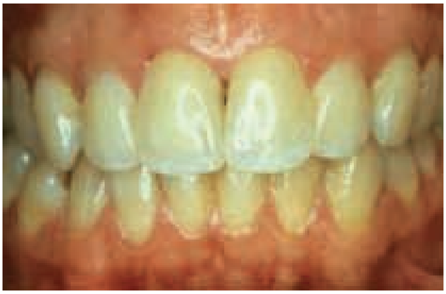

Plate 20

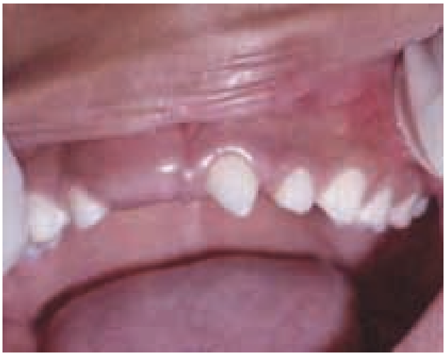

Plate 21

Explanatory notes

Plate 15 Code 3: filled with no caries – permanent dentition
Plate 16 Code 4: missing due to caries
Plate 17 Code 1: caries; code 4 – missing due to dental caries
Plate 18 Code 5: missing due to any other reason
Plate 19 Code 6: fissure sealant
Plate 20 Code 7: special crowns – teeth 11 and 21
Plate 21 Code 8: unerupted tooth

Periodontal status

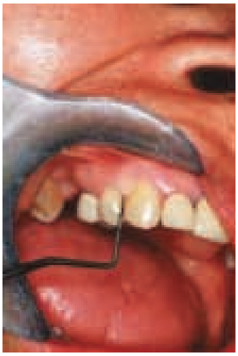

Plate 22

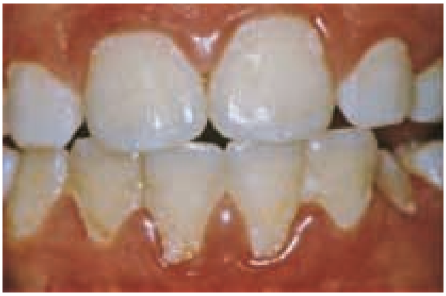

Plate 23

Explanatory notes

Plate 22 Gingival bleeding score 0: absence of condition
Plate 23 Gingival bleeding score 1: presence of condition (child)

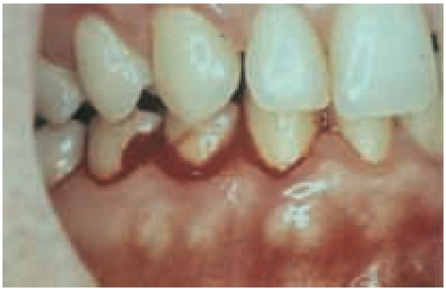

Plate 24

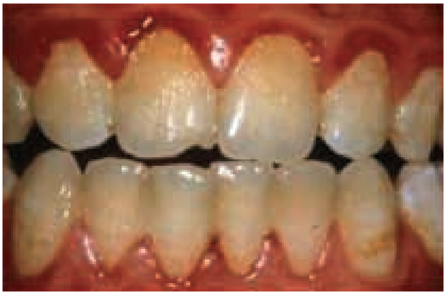

Plate 25

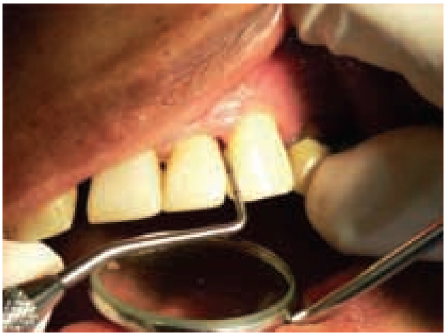

Plate 26

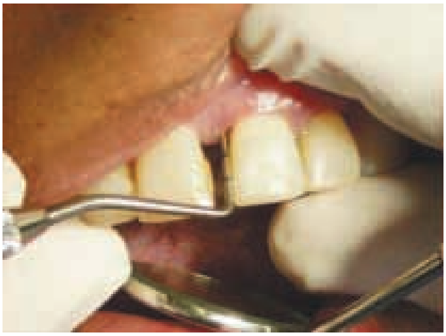

Plate 27

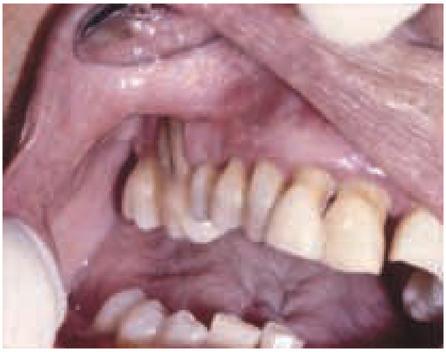

Plate 28

Plate 24 Gingival bleeding score 1: presence of condition (adolescent)
Plate 25 Gingival bleeding score 1: presence of condition (adult)
Plate 26 Pocket depth score 1: 4–5 mm
Plate 27 Pocket depth score 2: 6 mm or more
Plate 28 Gingival bleeding score 9: excluded – tooth 16

Enamel fluorosis

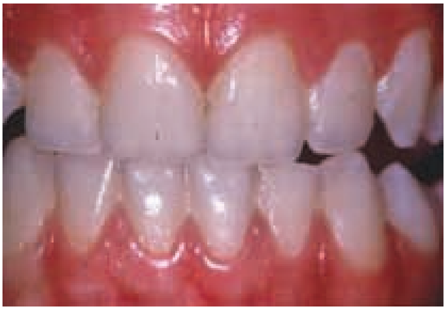

Plate 29

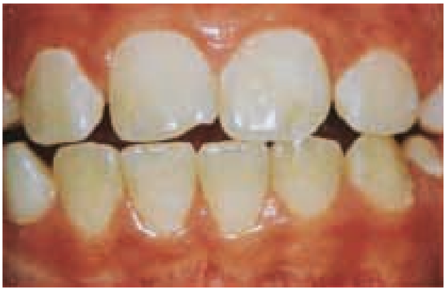

Plate 30

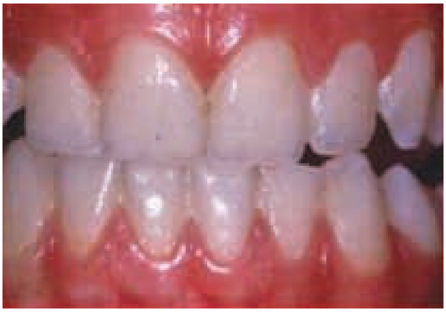

Plate 31

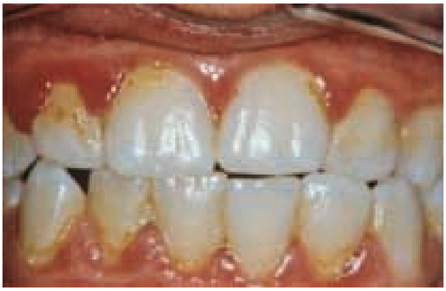

Plate 32

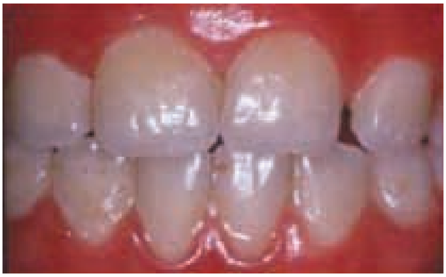

Plate 33

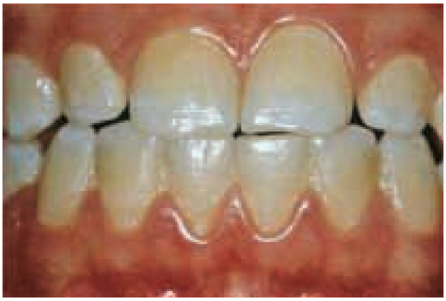

Plate 34

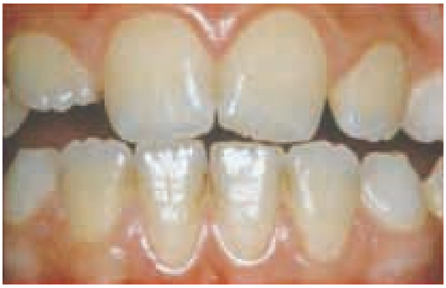

Plate 35

Explanatory notes

Plate 29 Code 0: normal/no sign of fluorosis
Plate 30 Score 1: questionable fluorosis
Plate 31 Score 1: questionable fluorosis
Plate 32 Score 1: questionable fluorosis
Plate 33 Score 1: questionable fluorosis
Plate 34 Score 2: very mild fluorosis
Plate 35 Score 2: very mild fluorosis

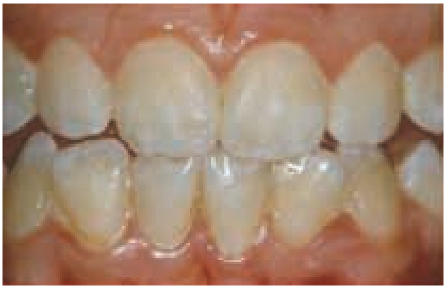

Plate 36

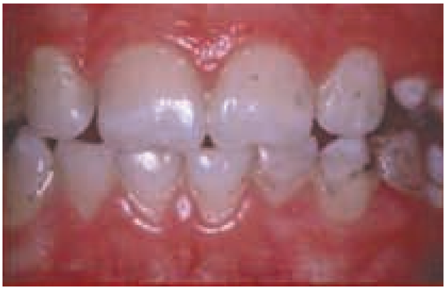

Plate 37

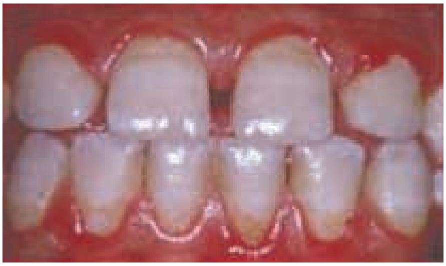

Plate 38

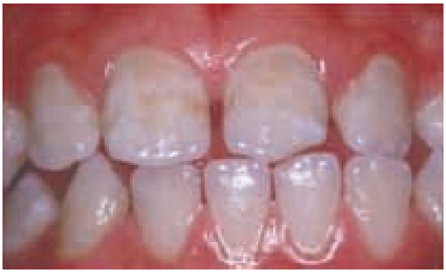

Plate 39

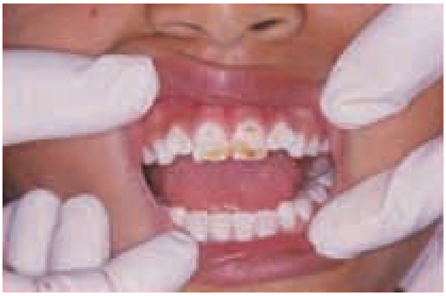

Plate 40

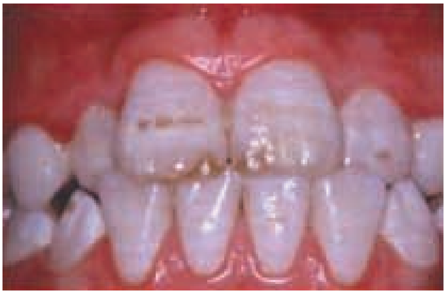

Plate 41

Explanatory notes

Plate 36 Score 3: mild fluorosis
Plate 37 Score 3: mild fluorosis
Plate 38 Score 4: moderate fluorosis
Plate 39 Score 4: moderate fluorosis
Plate 40 Score 5: severe fluorosis
Plate 41 Score 5: severe fluorosis

Dental erosion

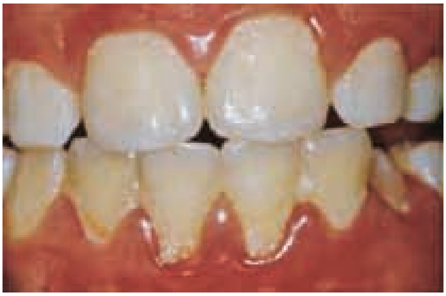

Plate 42

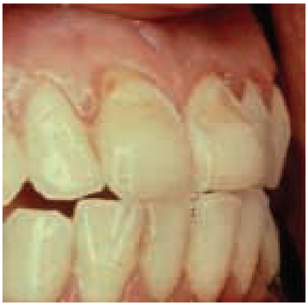

Plate 43

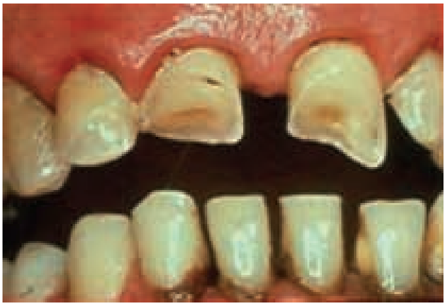

Plate 44

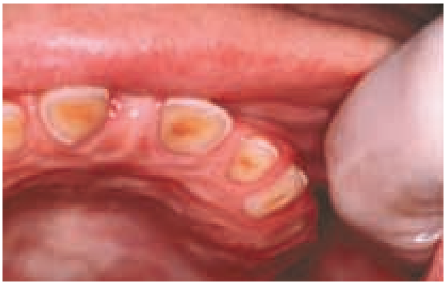

Plate 45

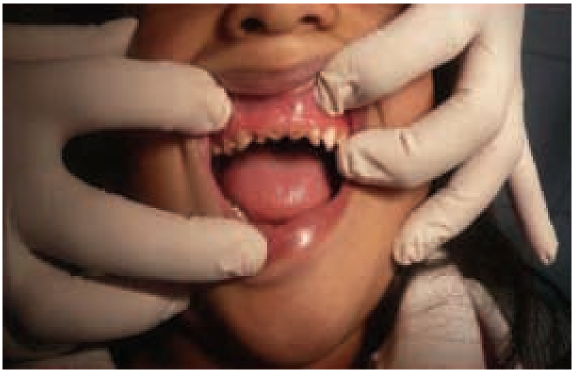

Plate 46

Explanatory notes

Plate 42 Code 0: no sign of erosion
Plate 43 Code 1: enamel lesion – tooth 11; score 2: dentinal lesion – tooth 21
Plate 44 Code 3: pulp involvement – teeth 11 and 21
Plate 45 Code 3: pulp involvement – teeth 11, 21, 22 and 23
Plate 46 Code 2: dentinal lesion – teeth 51 and 52; code 3: pulp involvement – tooth 61

Traumatic dental injuries

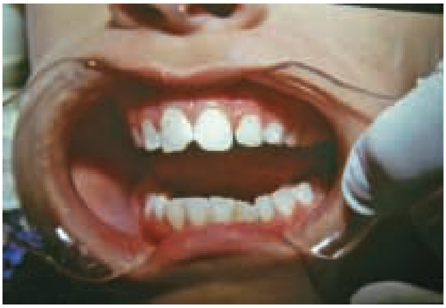

Plate 47

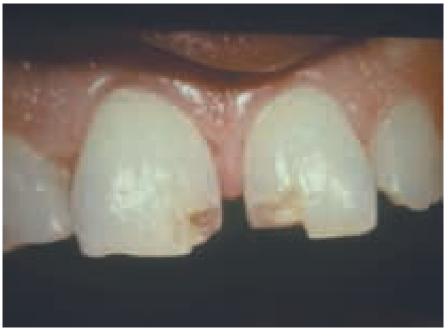

Plate 48

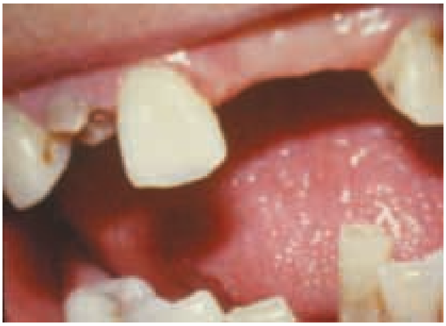

Plate 49

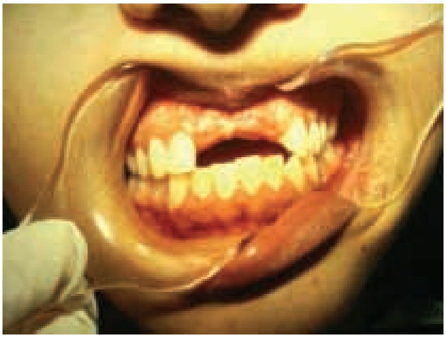

Plate 50

Explanatory notes

Plate 47 Code 2: enamel fracture only – teeth 11 and 21
Plate 48 Code 3: enamel and dentine fracture – teeth 11 and 21
Plate 49 Code 4: pulp involvement – tooth 12
Plate 50 Code 5: missing due to trauma – teeth 11, 21 and 22

Oral mucosal lesions

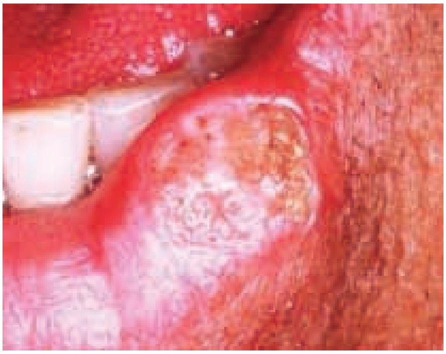

Plate 51

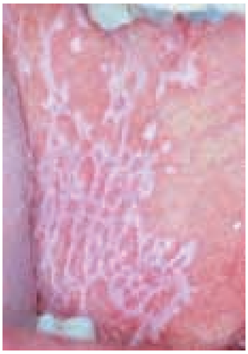

Plate 52

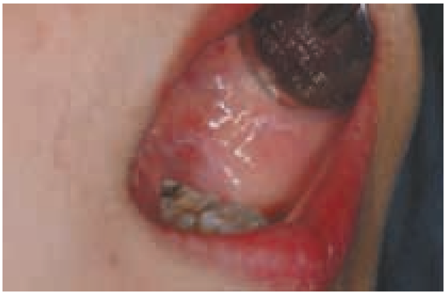

Plate 53

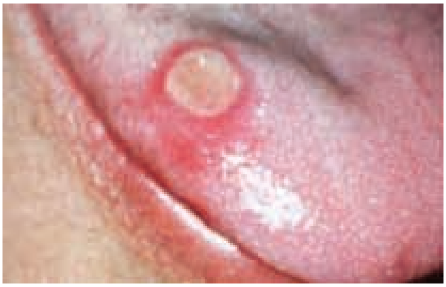

Plate 54

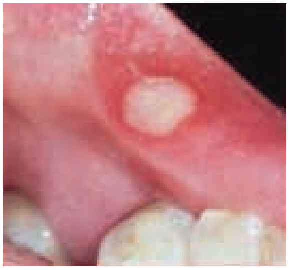

Plate 55

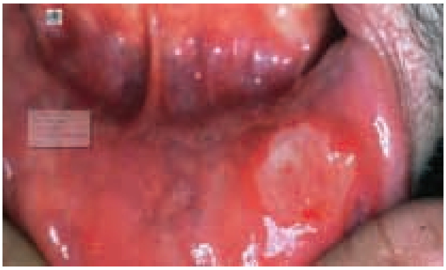

Plate 56

Explanatory notes

The first code is for the condition and the second for the location.

Plate 51 Code 1 and code 2: carcinoma of the lip
Plate 52 Code 2 and code 6: leukoplakia
Plate 53 Code 3 and code 4: lichen planus
Plate 54 Code 4 and code 6: aphthae on the tongue
Plate 55 Code 4 and code 1: recurrent aphthous ulceration
Plate 56 Code 4 and code 5: recurrent aphthous ulceration

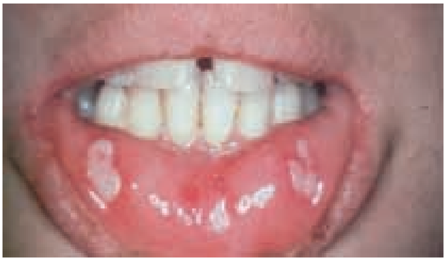

Plate 57

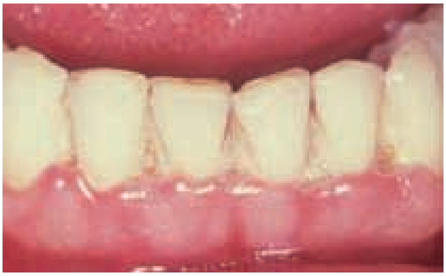

Plate 58

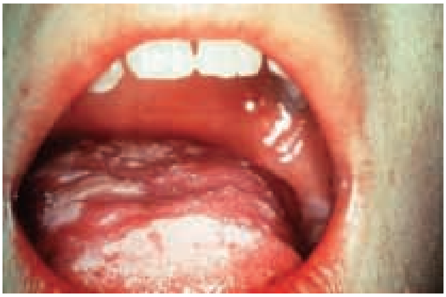

Plate 59

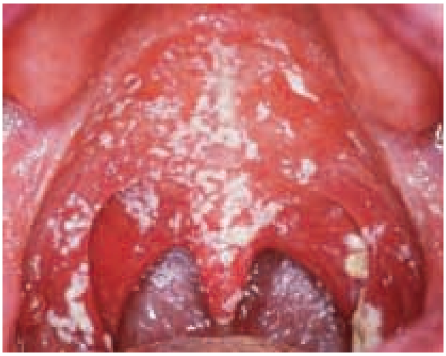

Plate 60

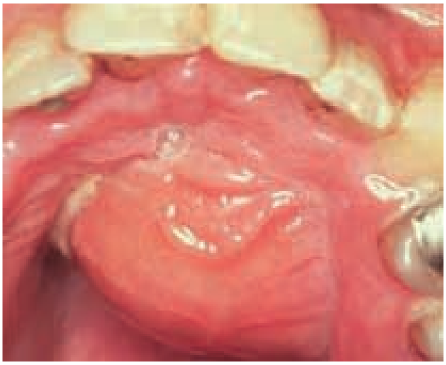

Plate 61

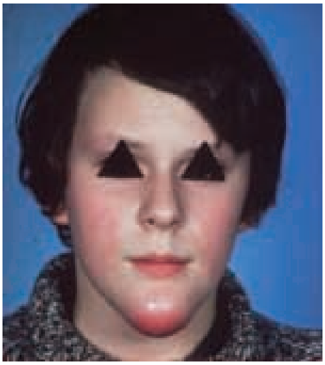

Plate 62

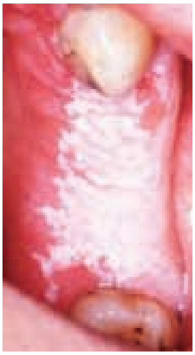

Plate 63

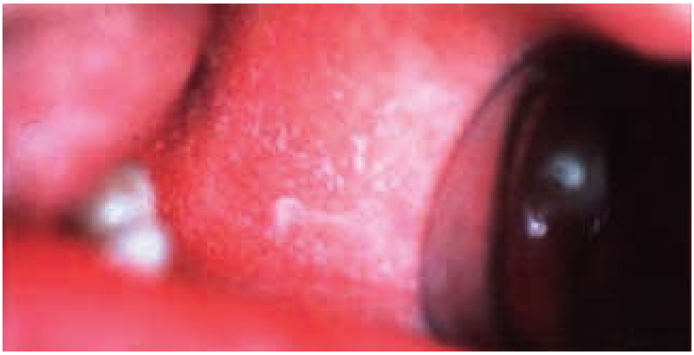

Plate 64

Explanatory notes

The first code is for the condition and the second for the location.

Plate 57 Code 4 and code 2: herpetic ulceration
Plate 58 Code 5 and code 8: acute necrotizing ulcerative gingivitis (ANUG)
Plate 59 Code 6 and code 6: candidiasis
Plate 60 Code 6 and code 7: candidiasis (thrush)
Plate 61 Code 7 and code 7: abscess
Plate 62 Code 7 and code 9: abscess
Plate 63 Code 8 and code 8: keratosis
Plate 64 Code 8 and code 6: Koplick spots

Intervention urgency

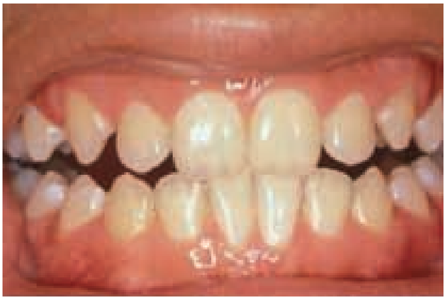

Plate 65

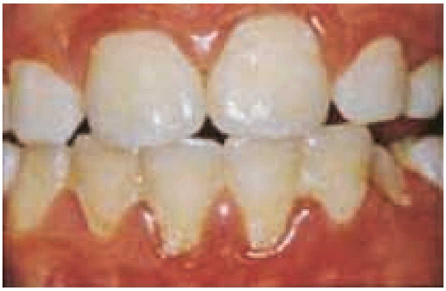

Plate 66

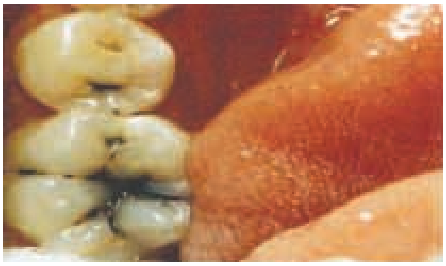

Plate 67

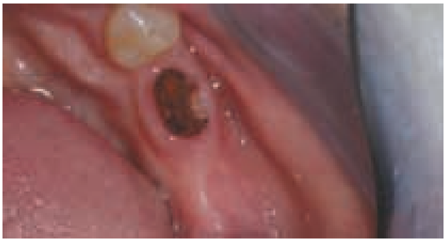

Plate 68

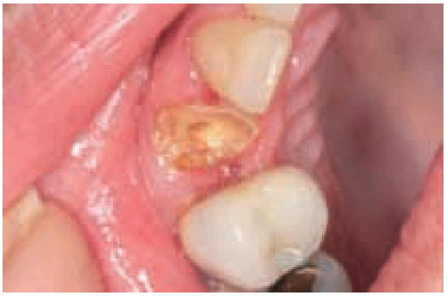

Plate 69

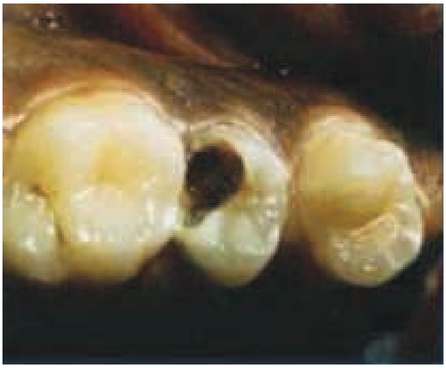

Plate 70

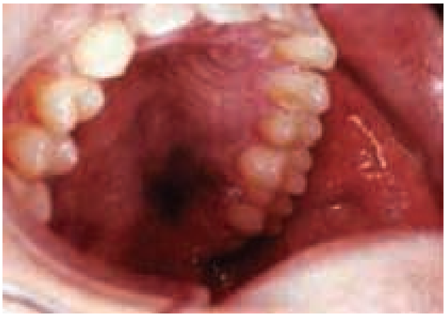

Plate 71

Explanatory notes

Plate 65 Code 0: no treatment needed
Plate 66 Code 1: preventive or routine treatment needed (scaling)
Plate 67 Code 2: prompt treatment needed
Plate 68 Code 3: immediate (urgent) treatment needed
Plate 69 Code 3: immediate (urgent) treatment needed
Plate 70 Code 3: immediate (urgent) treatment needed
Plate 71 Code 4: referred for comprehensive evaluation

World Health Organization

Oral Health Questionnaire for Adults

Identification number	Sex		Location		
	Male	Female	Urban	Periurban	Rural
1. ☐☐☐☐	☐	☐	☐	☐	☐
1 4	1	2	1	2	3

2. **How old are you today?** ______________________
(Years)

3. **How many natural teeth do you have?**

No natural teeth .... ☐ 0
1–9 teeth .... ☐ 1
10–19 teeth .... ☐ 2
20 teeth or more.............................. ☐ 3

4. **During the past 12 months, did your teeth or mouth cause any pain or discomfort?**

Yes .... ☐ 1
No .... ☐ 2
Don’t know .... ☐ 9
No answer .... ☐ 0

5. **Do you have any removable dentures?**

	Yes<br>1	No<br>2
A partial denture?..............................	☐	☐
A full upper denture?..............................	☐	☐
A full lower denture?	☐	☐

6. **How would you describe the state of your teeth and gums? Is it “excellent”, “very good”, “good”, “average”, “poor”, or “very poor”?**

	**Teeth**	**Gums**
Excellent	☐ 1	☐ 1
Very good..............................	☐ 2	☐ 2
Good	☐ 3	☐ 3
Average	☐ 4	☐ 4
Poor..............................	☐ 5	☐ 5
Very poor	☐ 6	☐ 6
Don’t know	☐ 9	☐ 9

7. How often do you clean your teeth?

- Never .... ☐ 1
- Once a month .... ☐ 2
- 2–3 times a month .... ☐ 3
- Once a week .... ☐ 4
- 2–6 times a week .... ☐ 5
- Once a day .... ☐ 6
- Twice or more a day .... ☐ 7

8. Do you use any of the following to clean your teeth?
(Read each item)

	Yes 1	No 2
Toothbrush	☐	☐
Wooden toothpicks	☐	☐
Plastic toothpicks?	☐	☐
Thread (dental floss)	☐	☐
Charcoal	☐	☐
Chewstick/miswak	☐	☐
Other	☐	☐
Please specify	☐	☐

9.

	Yes	No
**a) Do you use toothpaste to clean your teeth**	☐ 1	☐ 2
**b) Do you use a toothpaste that contains fluoride?**	☐ 1	☐ 2
Don't know	☐ 9	

10. How long is it since you last saw a dentist?

- Less than 6 months .... ☐ 1
- 6–12 months .... ☐ 2
- More than 1 year but less than 2 years .... ☐ 3
- 2 years or more but less than 5 years .... ☐ 4
- 5 years or more .... ☐ 5
- Never received dental care .... ☐ 6

11. What was the reason of your last visit to the dentist?

- Consultation/advise .... ☐ 1
- Pain or trouble with teeth, gums or mouth .... ☐ 2
- Treatment/ follow-up treatment .... ☐ 3
- Routine check-up/treatment .... ☐ 4
- Don't know/don't remember .... ☐ 5

12. **Because of the state of your teeth or mouth, how often have you experienced any of the following problems during the past 12 months?**

	Very often 4	Fairly often 3	Some-times 2	No 1	Don't know 0
(a) Difficulty in biting foods	☐	☐	☐	☐	☐
(b) Difficulty chewing foods............	☐	☐	☐	☐	☐
(c) Difficulty with speech/trouble pronouncing words	☐	☐	☐	☐	☐
(d) Dry mouth...............................	☐	☐	☐	☐	☐
(e) Felt embarrassed due to appearance of teeth................	☐	☐	☐	☐	☐
(f) Felt tense because of problems with teeth or mouth	☐	☐	☐	☐	☐
(g) Have avoided smiling because of teeth.....................	☐	☐	☐	☐	☐
(h) Had sleep that is often interrupted	☐	☐	☐	☐	☐
(i) Have taken days off work	☐	☐	☐	☐	☐
(j) Difficulty doing usual activities..	☐	☐	☐	☐	☐
(k) Felt less tolerant of spouse or people who are close to you..................................	☐	☐	☐	☐	☐
(l) Have reduced participation in social activities..................	☐	☐	☐	☐	☐

13. **How often do you eat or drink any of the following foods, even in small quantities?**
(Read each item)

	Several times a day 6	Every day 5	Several times a week 4	Once a week 3	Several times a month 2	Seldom /never 1
Fresh fruit..................	☐	☐	☐	☐	☐	☐
Biscuits, cakes, cream cakes	☐	☐	☐	☐	☐	☐
Sweet pies, buns..........	☐	☐	☐	☐	☐	☐
Jam or honey	☐	☐	☐	☐	☐	☐
Chewing gum containing sugar	☐	☐	☐	☐	☐	☐
Sweets/candy..............	☐	☐	☐	☐	☐	☐

	Every day	Several times a week	Once a week	Several times a month	Seldom	Never
Lemonade, Coca Cola or other soft drinks ..	☐	☐	☐	☐	☐	☐
Tea with sugar	☐	☐	☐	☐	☐	☐
Coffee with sugar	☐	☐	☐	☐	☐	☐

(Insert country-specific items)

14. **How often do you use any of the following types of tobacco?** (Read each item)

	Every day	Several times a week	Once a week	Several times a month	Seldom	Never
	6	5	4	3	2	1
Cigarettes	☐	☐	☐	☐	☐	☐
Cigars	☐	☐	☐	☐	☐	☐
A pipe	☐	☐	☐	☐	☐	☐
Chewing tobacco	☐	☐	☐	☐	☐	☐
Use snuff....................	☐	☐	☐	☐	☐	☐
Other	☐	☐	☐	☐	☐	☐

Please specify ______________________________

15. **During the past 30 days, on the days you drank alcohol, how many drinks did you *usually drink per day*?**

Less than 1 drink .... ☐ 0
1 drink.. ☐ 1
2 drinks .... ☐ 2
3 drinks .... ☐ 3
4 drinks .... ☐ 4
5 or more drinks .... ☐ 5
Did not drink alcohol during the past 30 days .... ☐ 9

16. **What level of education have you completed?**

No formal schooling.. ☐ 1
Less than primary school... ☐ 2
Primary school completed .... ☐ 3
Secondary school completed.. ☐ 4
High school completed.. ☐ 5
College/university completed .... ☐ 6
Postgraduate degree .... ☐ 7
(Insert country-specific categories)

That completes our questionnaire
Thank you very much for your cooperation!

Year	Month	Day	Interviewer	District	Country
☐☐	☐☐	☐☐	☐☐	☐☐	☐☐☐☐

World Health Organization

Oral Health Questionnaire for Children

First, we would like you to answer some questions concerning yourself and your teeth

**Identification number**	**Sex**		**Location**		
	Boy	Girl	Urban	Periurban	Rural
1. ☐☐☐☐	☐	☐	☐	☐	☐
1 4	1	2	1	2	3

2. **How old are you today?** ______________________
(Years)

3. **How would you describe the health of your teeth and gums?**
(Read each item)

	**Teeth**	**Gums**
Excellent	☐ 1	☐ 1
Very good	☐ 2	☐ 2
Good	☐ 3	☐ 3
Average	☐ 4	☐ 4
Poor	☐ 5	☐ 5
Very poor	☐ 6	☐ 6
Don't know	☐ 9	☐ 9

4. **How often during the past 12 months did you have toothache or feel discomfort due to your teeth?**

Often	☐ 1
Occasionally	☐ 2
Rarely	☐ 3
Never	☐ 4
Don't know	☐ 9

Now please answer some questions about the care of your teeth

5. **How often did you go to the dentist during the past 12 months?**
(Put a tick/cross in one only)

Once	☐ 1
Twice	☐ 2
Three times	☐ 3
Four times	☐ 4

More than four times .... ☐ 5
I had no visit to dentist during the past 12 months.................. ☐ 6
I have never received dental care/visited a dentist................... ☐ 7
I don't know/don't remember .... ☐ 9

If you did not see a dentist during the last 12 months, go on to question 7

6. **What was the reason for your last visit to the dentist?**
(Put a tick/cross in one box only)
Pain or trouble with teeth, gums or mouth .... ☐ 1
Treatment/follow-up treatment .... ☐ 2
Routine check-up of teeth/treatment...................................... ☐ 3
I don't know/don't remember .... ☐ 9

7. **How often do you clean your teeth?**
(Put a tick/cross in one box only)
Never.. ☐ 1
Several times a month (2–3 times).. ☐ 2
Once a week .... ☐ 3
Several times a week (2–6 times) .... ☐ 4
Once a day.. ☐ 5
2 or more times a day .... ☐ 6

8. **Do you use any of the following to clean your teeth or gums?**
(Read each item)

	Yes 1	No 2
Toothbrush..	☐	☐
Wooden toothpicks	☐	☐
Plastic toothpicks..	☐	☐
Thread (dental floss)	☐	☐
Charcoal	☐	☐
Chewstick/miswak...	☐	☐
Other	☐	☐

Please specify____

9.

	Yes	No
a) **Do you use toothpaste to clean your teeth**	☐ 1	☐ 2
b) **Do you use toothpaste that contains fluoride?**....	☐ 1	☐ 2
Don't know...........	☐ 9	

10. **Because of the state of your teeth and mouth, have you experienced any of the following problems during the past year?**

	Yes 1	No 2	Don't know 0
(a) I am not satisfied with the appearance of my teeth	☐	☐	☐
(b) I often avoid smiling and laughing because of my teeth	☐	☐	☐
(c) Other children make fun of my teeth	☐	☐	☐
(d) Toothache or discomfort caused by my teeth forced me to miss classes at school or miss school for whole days	☐	☐	☐
(e) I have difficulty biting hard foods	☐	☐	☐
(f) I have difficulty in chewing	☐	☐	☐

11. **How often do you eat or drink any of the following foods, even in small quantities?**
(Read each item)

	Several times a day 6	Every day 5	Several times a week 4	Once a week 3	Several times a month 2	Never 1
Fresh fruit	☐	☐	☐	☐	☐	☐
Biscuits, cakes, cream cakes, sweet pies, buns etc	☐	☐	☐	☐	☐	☐
Lemonade, Coca Cola or other soft drinks	☐	☐	☐	☐	☐	☐
Jam/honey	☐	☐	☐	☐	☐	☐
Chewing gum containing sugar	☐	☐	☐	☐	☐	☐
Sweets/candy	☐	☐	☐	☐	☐	☐
Milk with sugar	☐	☐	☐	☐	☐	☐
Tea with sugar	☐	☐	☐	☐	☐	☐
Coffee with sugar	☐	☐	☐	☐	☐	☐

(Insert country-specific items)

12. **How often do you use any of the following types of tobacco?** (Read each item)

	Every day	Several times a week	Once a week	Several times a month	Seldom	Never
	6	5	4	3	2	1
Cigarettes, pipe or cigars ...	☐	☐	☐	☐	☐	☐
Chewing tobacco or snuff ..	☐	☐	☐	☐	☐	☐

13. **What level of education has your father completed (or your stepfather, guardian or other male adult living with you)?**

No formal schooling.. ☐ 1
Less than primary school.. ☐ 2
Primary school completed .... ☐ 3
Secondary school completed.. ☐ 4
High school completed .... ☐ 5
College/university completed .... ☐ 6
No male adult in household .... ☐ 7
Don't know.. ☐ 9

14. **What level of education has your mother completed?**

No formal schooling.. ☐ 1
Less than primary school.. ☐ 2
Primary school completed .... ☐ 3
Secondary school completed.. ☐ 4
High school completed .... ☐ 5
College/university completed .... ☐ 6
No female adult in household.. ☐ 7
Don't know.. ☐ 9
(Insert country-specific categories)

That completes our questionnaire
Thank you very much for your cooperation!

Year	Month	Day	Interviewer	District	Country
☐☐	☐☐	☐☐	☐☐	☐☐	☐☐☐☐

Standard tables generated from clinical survey data

The following descriptive tables can be produced by a standard computer programme (Epi Info, SPSS) from the clinical data collected in a basic oral health survey. Standard results are produced for the total sample and for each pertinent subgroup, i.e. by the following indicator age groups: 5–6, 12, 15–19, 35–44 and 65–74 years. Tabular data are disaggregated by age group and sex, and may be further stratified by ethnic group, education, occupation, geographical location and type.

General information

Table 1 Distribution of total sample by sex and age or age group
Table 2 Distribution of total sample by ethnic group
Table 3 Distribution of total sample by occupation
Table 4 Distribution of total sample by geographical location
Table 5 Distribution of total sample by type of location
Table 6 Other data – number of subjects by code (Boxes 37–42)
Table 7 Distribution of total sample by extra-oral conditions

Clinical assessment

Table 8 Mean number of primary teeth present per person.

Table 9 Number and percentage of subjects with caries of the primary dentition; number and percentage of subjects with untreated caries of the primary teeth; number and percentage of subjects with specific value of dmft (decayed, missing due to caries and filled primary teeth).

Table 10 Mean number of decayed primary teeth (dt) per person; mean number of filled primary teeth (ft) per person; mean number of missing primary teeth (mt) per person; mean number of primary teeth with caries experience (dmft) per person.

Table 11 Mean number of permanent teeth present per person.

Table 12 Number and percentage of subjects who have or have had caries of the permanent dentition (DMFT); number and percentage of subjects with untreated caries (DT); number and percentage of subjects with specific value of DMFT.

Table 13 Mean number of decayed permanent teeth (DT) per person; mean number of filled permanent teeth (FT) per person; mean number of permanent teeth missing due to caries (MT) per person; mean number of permanent teeth with caries experience (DMFT) per person.

Table 14 Number and percentage of subjects with and without natural teeth.

Table 15 Number and percentage of persons with fissure sealants.

Table 16 Number and percentage of adult subjects with root caries.

Table 17 Mean number of teeth per adult person with root caries.

Table 18 Number and percentage of adult subjects with coronal and/or root caries.

Table 19 Mean number of teeth per adult person with coronal and/or root caries.

Table 20 Number and percentage of subjects with healthy periodontal conditions.

Table 21 Number and percentage of subjects with gingival bleeding.

Table 22 Based on highest score, number and percentage of subjects with shallow pockets; number and percentage of subjects with deep pockets (≥6 mm).

Table 23 Mean number and mean percentage of teeth present with gingival bleeding.

Table 24 Mean number and mean percentage of teeth present with shallow pockets (4–5 mm).

Table 25 Mean number and mean percentage of teeth present with deep pockets (≥6 mm).

Table 26 Number and percentage of subjects with loss of attachment, by highest score.

Table 27 Mean number of sextants with loss of attachment, by score; mean number of sextants excluded from examination; mean number of sextants not recorded.

Table 28 Number and percentage of subjects with enamel fluorosis, by level of severity.

Table 29 Children Age Specific Community Fluorosis (CFI) Index: ages 5–6 (if any), 12 and 15, by region.

Table 30 Number and percentage of persons with dental erosion, by severity; mean number and percentage of teeth affected by dental erosion per person.

Table 31 Number and percentage of persons affected with traumatic injuries; mean number and percentage of teeth with treated injuries; mean number and percentage of enamel fractures; mean number and percentage of enamel and dentine fractures; mean number and percentage of fractures involving the pulp; mean number and percentage of teeth missing due to trauma; mean number and

percentage of other damage caused by trauma; mean number and percentage of teeth affected by trauma per person.

Table 32 Number and percentage of subjects with no abnormalities of the oral mucosa; number and percentage of subjects with oral mucosal lesions: malignant tumour (oral cancer), leukoplakia, lichen planus, ulceration (aphthous, herpetic, traumatic), acute necrotizing gingivitis, candidiasis, abscess, or other condition.

Table 33 Number and percentage of subjects with removable denture(s) in the upper or lower jaws.

Table 34 Number and percentage of subjects in urgent need of a different intervention.

Standard tables generated from STEPS questionnaire data

The following tables are suggested as standard tables that should be generated from an oral health survey, including the questionnaire component. The questionnaire may have been implemented as a stand-alone activity. Tables should include data disaggregated by WHO indicator age groups and sex and also standard background variables, e.g. ethnic group, geographical location, type of location, occupation, and education.

General information is described according to the guidelines for the clinical oral health survey.

Adult questionnaire

Table 1 Percentage of respondents who have no natural teeth, 1–9 natural teeth, 10–19 natural teeth, or 20 or more natural teeth.

Table 2 Percentage of respondents with pain or discomfort caused by their teeth or mouth during the past 12 months.

Table 3 Percentage of respondents who have removable dentures.

Table 4 Percentage of respondents who have a maxillary denture, a mandibular denture, or a maxillary and a mandibular denture.

Table 5 Percentage of respondents having poor or very poor state of teeth among those having natural teeth

Table 6 Percentage of respondents having poor or very poor state of gums among those having natural teeth.

Table 7 Percentage of respondents by frequency of tooth cleaning.

Table 8 Percentage of respondents who never clean their teeth.

Table 9 Percentage of respondents who use a toothbrush, wooden toothpicks, plastic toothpicks, thread (dental floss), charcoal, chewstick/miswak or something else to clean their teeth.

Table 10 Percentage of respondents using toothpaste among those cleaning their teeth.

Table 11 Percentage of respondents using toothpaste containing fluoride among those using toothpaste.

Table 12 Percentage of respondents who have never received dental care.

Table 13 Percentage of respondents by time since last visit to a dentist.

Table 14 Main reason for last visit to the dentist among those who have ever visited a dentist.

Table 15 Percentage of respondents experiencing difficulty biting/chewing foods during the past 12 months.
Table 16 Percentage of respondents having difficulty with speech/enunciation during the past 12 months.
Table 17 Percentage of respondents feeling embarrassed about the appearance of their teeth during the past 12 months.
Table 18 Percentage of respondents who felt tense because of problems with their teeth or mouth during the past 12 months.
Table 19 Percentage of respondents who avoid smiling because of their teeth during the past 12 months.
Table 20 Percentage of respondents whose sleep was often interrupted during the past 12 months.
Table 21 Percentage of respondents with days not at work because of their teeth or mouth during the past 12 months.
Table 22 Percentage of respondents experiencing difficulty undertaking their usual activities during the past 12 months.
Table 23 Percentage of respondents less tolerant of spouse or people close to them during the past 12 months.
Table 24 Percentage of respondents with diminished participation in social activities during the past 12 months.
Table 25 Percentage of respondents by frequency of consumption of various types of foods/drinks.
Table 26 Percentage of respondents by frequency of consumption of various types of tobacco.
Table 27 Percentage of respondents by daily consumption of alcoholic drinks.
Table 28 Percentage of respondents by level of education completed.

Questionnaire for children

Table 1 Percentage of children having poor or very poor state of teeth.
Table 2 Percentage of children having poor or very poor state of gums.
Table 3 Percentage of children having experience of pain or discomfort in their teeth or mouth during the past 12 months.
Table 4 Percentage of children by frequency of visit to a dentist during the past 12 months.
Table 5 Percentage of children with no visit to a dentist during the past 12 months.
Table 6 Percentage of children who have never been to a dentist.
Table 7 Percentage of children by main reason of last visit to a dentist.
Table 8 Percentage of children by frequency of tooth cleaning.
Table 9 Percentage of children who never clean their teeth.

Table 10 Percentage of children who use a toothbrush, wooden toothpick, plastic toothpick, thread/dental floss, charcoal, or chewstick/miswak for cleaning their teeth.
Table 11 Percentage of children who use toothpaste for cleaning their teeth.
Table 12 Percentage of children who use fluoridated toothpaste for cleaning their teeth.
Table 13 Percentage of children not feeling satisfied with appearance of their teeth.
Table 14 Percentage of children who often avoid smiling and laughing because of their teeth.
Table 15 Percentage of children reporting that other children make fun of their teeth.
Table 16 Percentage of children who miss classes at school because of pain or discomfort from teeth.
Table 17 Percentage of children with difficulty biting hard foods.
Table 18 Percentage of children with difficulty chewing foods.
Table 19 Percentage of children by frequency of consumption of various foods/drinks.
Table 20 Percentage of children by frequency of consumption of tobacco.
Table 21 Percentage of children by level of education of father.
Table 22 Percentage of children by level of education of mother.

Dépôt légal : novembre 2013 N° imprimeur : 111346598
Imprimé en France par Présence Graphique - Monts
