# Supplementary figures and images for: Assessment of the Oral Health Perceptions and Behaviours of Adolescents in Bosnia and Herzegovina: A Cross Sectional Study
Source: Healthcare (Basel). 2025 Jun 5;13(11):1347. doi: 10.3390/healthcare13111347 (PMC12155286; doi:10.3390/healthcare13111347)

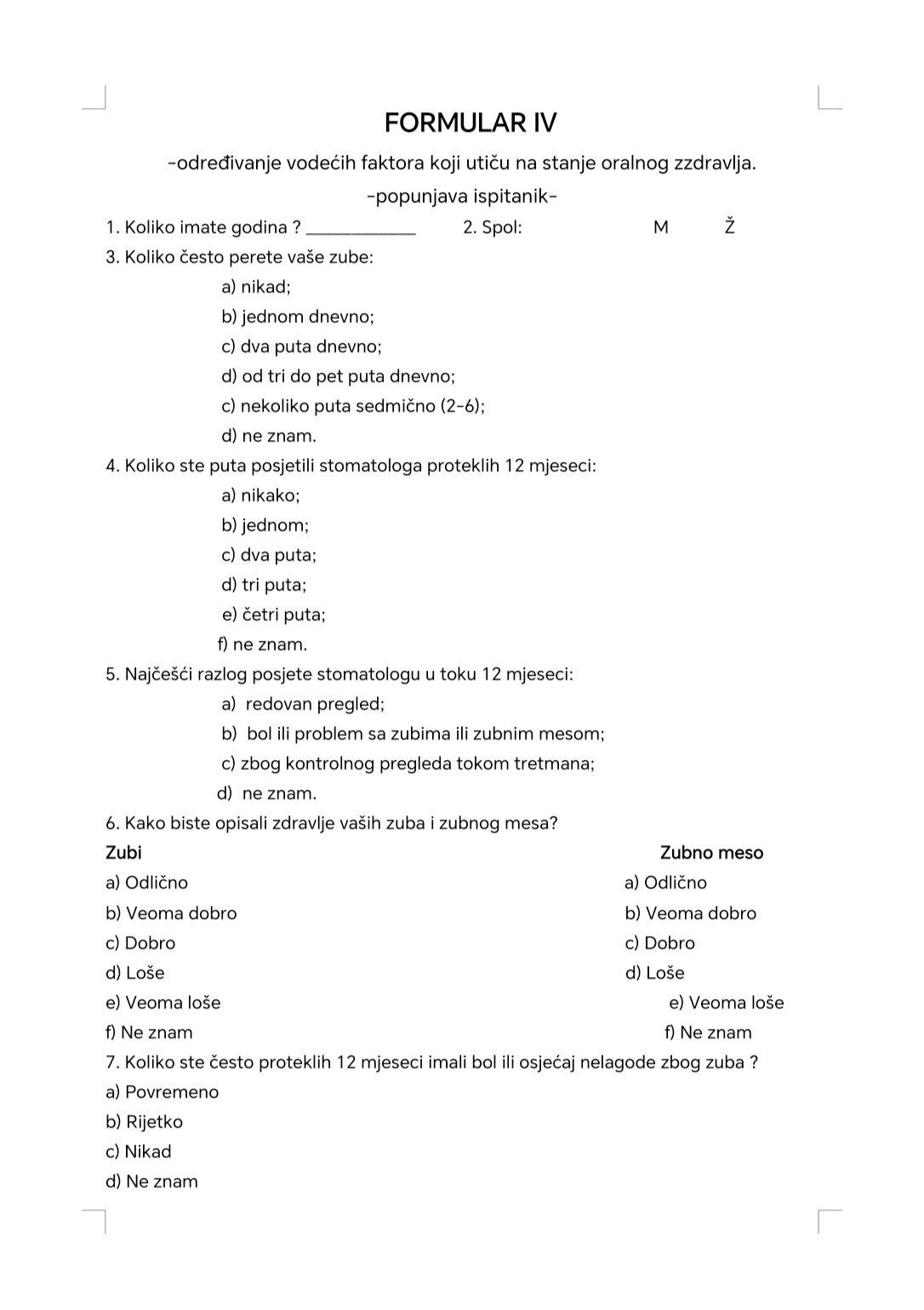

Supplement: Supplementary file 1 [file healthcare-13-01347-s001.zip › Questionnaire IV - 1.jpeg]

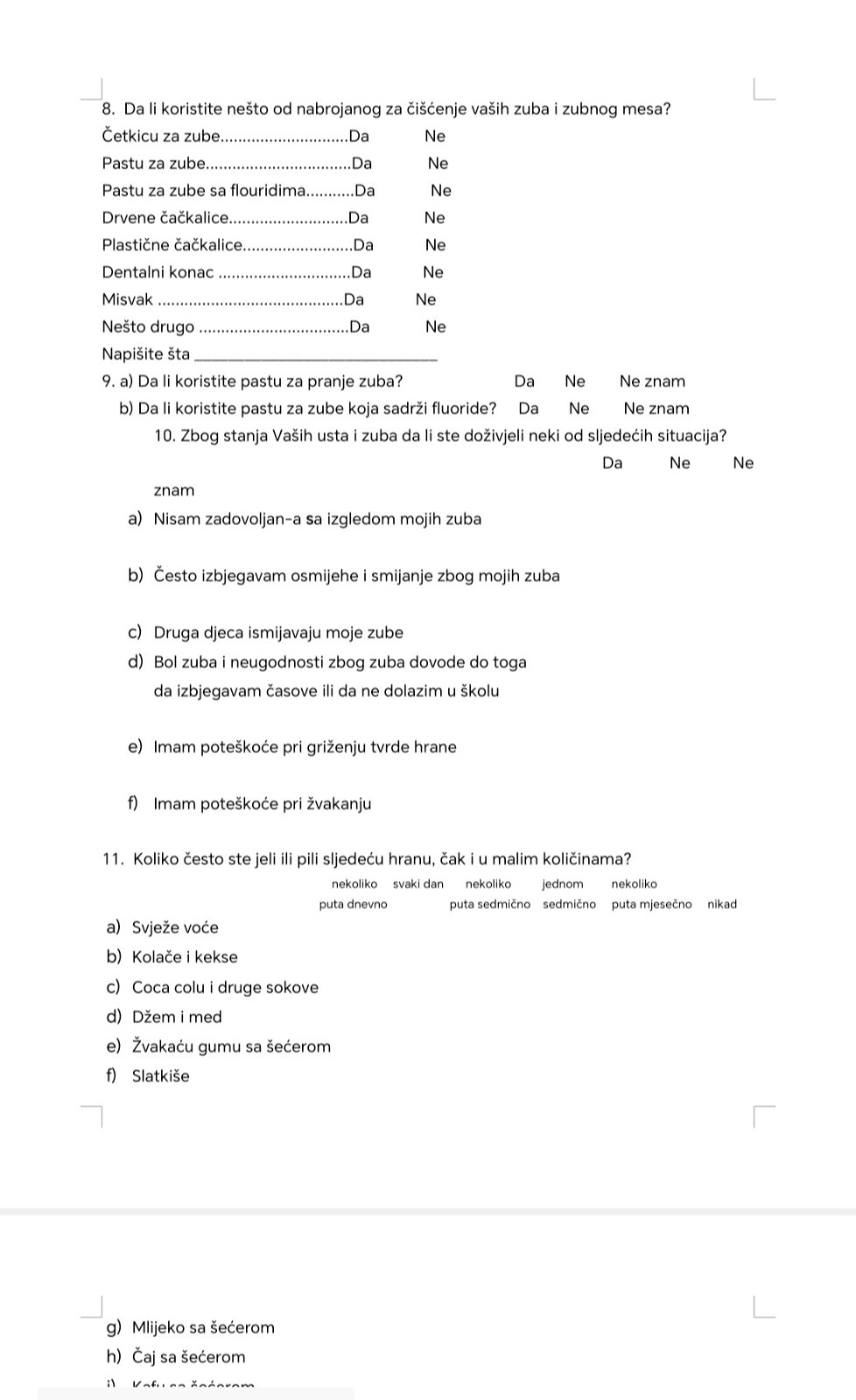

Supplement: Supplementary file 1 [file healthcare-13-01347-s001.zip › Questionnaire IV - 2.jpeg]

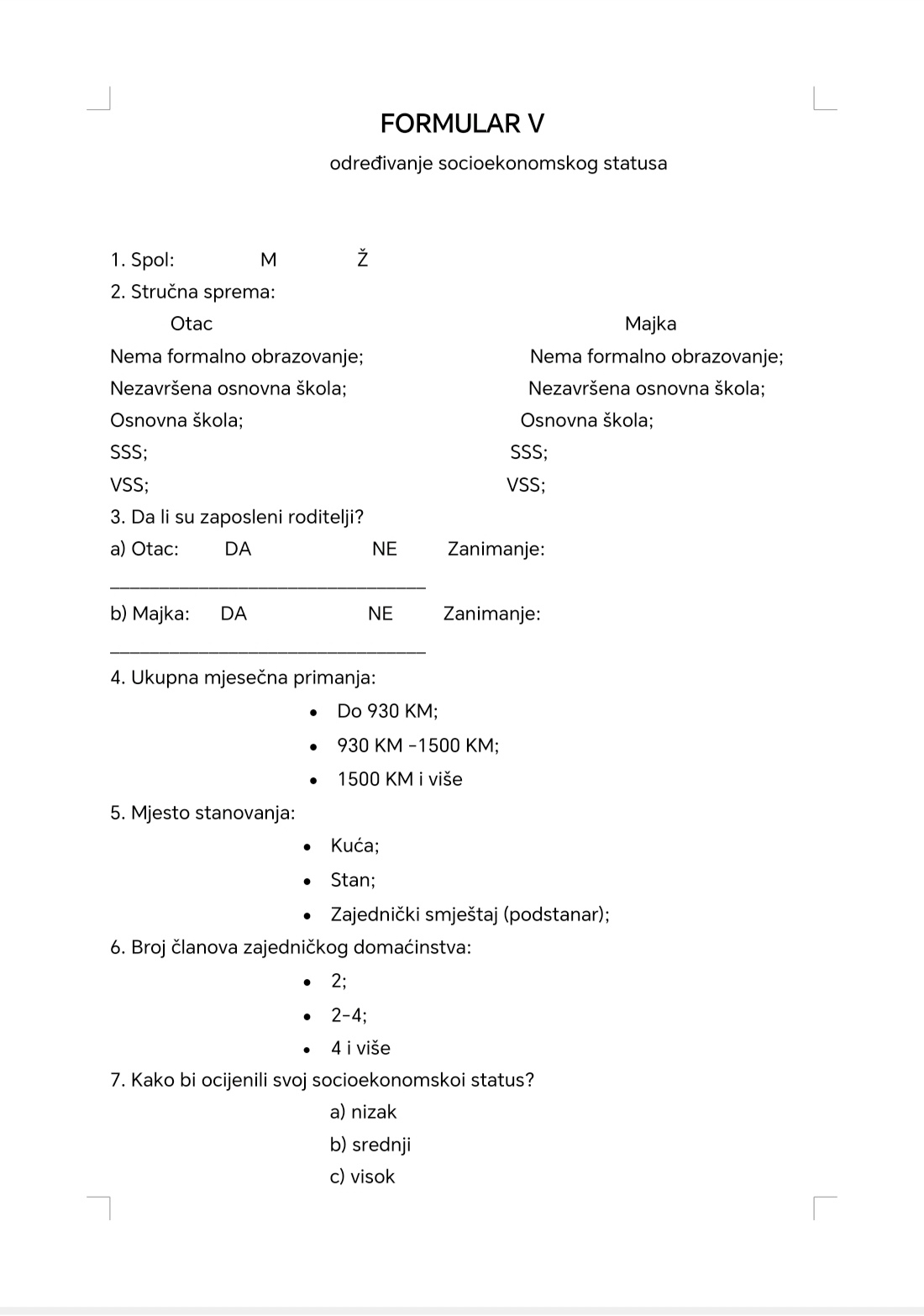

Supplement: Supplementary file 1 [file healthcare-13-01347-s001.zip › Questionnaire V.jpeg]
